# Polycationic Monomeric and Homodimeric Asymmetric Monomethine Cyanine Dyes with Hydroxypropyl Functionality—Strong Affinity Nucleic Acids Binders

**DOI:** 10.3390/biom11081075

**Published:** 2021-07-21

**Authors:** Ivana Mikulin, Ivana Ljubić, Ivo Piantanida, Aleksey Vasilev, Mihail Mondeshki, Meglena Kandinska, Lidija Uzelac, Irena Martin-Kleiner, Marijeta Kralj, Lidija-Marija Tumir

**Affiliations:** 1 Laboratory for Biomolecular Interactions and Spectroscopy, Division of Organic Chemistry and Biochemistry, Ruđer Bošković Institute, P.O. Box 180, 10002 Zagreb, Croatia; ivana.mikulin@skole.hr (I.M.); ivana.soric@gmail.com (I.L.); pianta@irb.hr (I.P.); 2 Faculty of Chemistry and Pharmacy, Department of Pharmaceutical and Applied Organic Chemistry, Sofia University “St. Kliment Ohridski”, 1000 Sofia, Bulgaria; meglenakandinska@gmail.com; 3 Department of Chemistry, Johannes Gutenberg Universität Mainz, Duesbergweg 10–14, 55128 Mainz, Germany; mondeshk@uni-mainz.de; 4 Laboratory of Experimental Therapy, Division of Molecular Medicine, Ruđer Bošković Institute, P.O. Box 180, 10002 Zagreb, Croatia; Lidija.Uzelac@irb.hr (L.U.); Irena.Martin-Kleiner@irb.hr (I.M.-K.); Marijeta.Kralj@irb.hr (M.K.)

**Keywords:** cyanine dye, DNA binding, RNA binding, fluorescence, circular dichroism, antiproliferative activity

## Abstract

New analogs of the commercial asymmetric monomethine cyanine dyes thiazole orange (TO) and thiazole orange homodimer (TOTO) with hydroxypropyl functionality were synthesized and their properties in the presence of different nucleic acids were studied. The novel compounds showed strong, micromolar and submicromolar affinities to all examined DNA ds-polynucleotides and poly rA–poly rU. The compounds studied showed selectivity towards GC-DNA base pairs over AT-DNA, which included both binding affinity and a strong fluorescence response. CD titrations showed aggregation along the polynucleotide with well-defined supramolecular chirality. The single dipyridinium-bridged dimer showed intercalation at low dye-DNA/RNA ratios. All new cyanine dyes showed potent micromolar antiproliferative activity against cancer cell lines, making them promising theranostic agents.

## 1. Introduction

The binding of small molecules to DNA/RNA was of scientific interest for decades because of the enormous biological significance of polynucleotides. Formation of the supramolecular complex of small molecule–DNA/RNA could influence processes in the living cell, like DNA replication, transcription to RNA and consequently protein synthesis [1,2,3]. Thus, many drugs owe their activity to a non-covalent interaction with DNA/RNA [4,5].

Usually, a small molecule is bound to a double-stranded (ds) polynucleotide by intercalation between base pairs, minor or major groove binding and/or external electrostatic binding [5]. There is a challenging task to design and synthesize a small molecule capable of strong binding combined with specific recognition of a certain structure type (e.g., AT-rich sequence or GC-rich sequence, DNA or RNA, single-stranded or double-stranded helix, a different type of helix, like A, B, Z, etc.) and also a specific binding response [6,7]. A combination of different functionalities and structural building blocks within the same ligand molecule could result in an enhanced number of non-covalent interactions, increased affinity and selective recognition of specific DNA/RNA structure. The change in the strength of these non-covalent interactions with the correspondent bioobjects can cause dramatic shifts in the photophysical properties of the fluorochromes. If the aggregation of the dyes on the surface of the target biomacromolecules produce the appearance of new absorption and/or new fluorescent signals or shifts of the existing ones, the commented phenomena are defined as biochromism and can be used as an indicator for the presence of definite bioanalytes.

The cyanine dyes are well known DNA/RNA fluorescent probes, with various bioanalytical applications, due to their strong enhancement of fluorescence upon binding to biomacromolecules [8]. Among cyanines, **TOTO** dye families showed high sensitivity comparable to radioactive probes [9]. Structural modifications and the introduction of different substituents take advantage to modify binding modes and affinities and signal upon complex formation. These modifications represent a model for studying the structure–function relationship [8].

In our previous work, we investigated the influence of steric restriction on cyanine dyes binding. It was noticed that dyes with bulky substituents on their long axis preferred minor groove binding instead of intercalation [10]. Asymmetric cyanines with large phosphonium substituents showed kinetic differentiation between homo and alternating AT-DNA sequences upon forming dimers within the DNA minor groove. They also discriminated homo and alternating GC-DNA sequences by the selectively induced CD signal [11]. Additional methylation of dyes yielded by the absence of kinetic AT selectivity, but also the appearance of the AT selective fluorescence response [12]. Chloro substitution of the oxazole yellow homodimer (YOYO) resulted in a specific fluorimetric response of ds-RNA compared to ds-DNA, while the chlorinated TOTO analogue revealed specific CD recognition of alternating-DNA sequences concerning homo-DNA and homo-RNA sequences [13]. Monomethine cyanine dyes based on thiazolo [4,5-b]pyridinium and quinolinium chromophores revealed higher sensitivity to DNA when compared to commercial dimeric cyanine dye TOTO-1 [14]. Further, we prepared a new series of monomeric and homodimeric cyanine dyes that represented structural modifications of TO (thiazole orange) and TOTO (thiazole orange homodimer) dyes: thioacetyl substituents and 2-hydroxypropyl substituents [15,16].

**Scheme 1:**
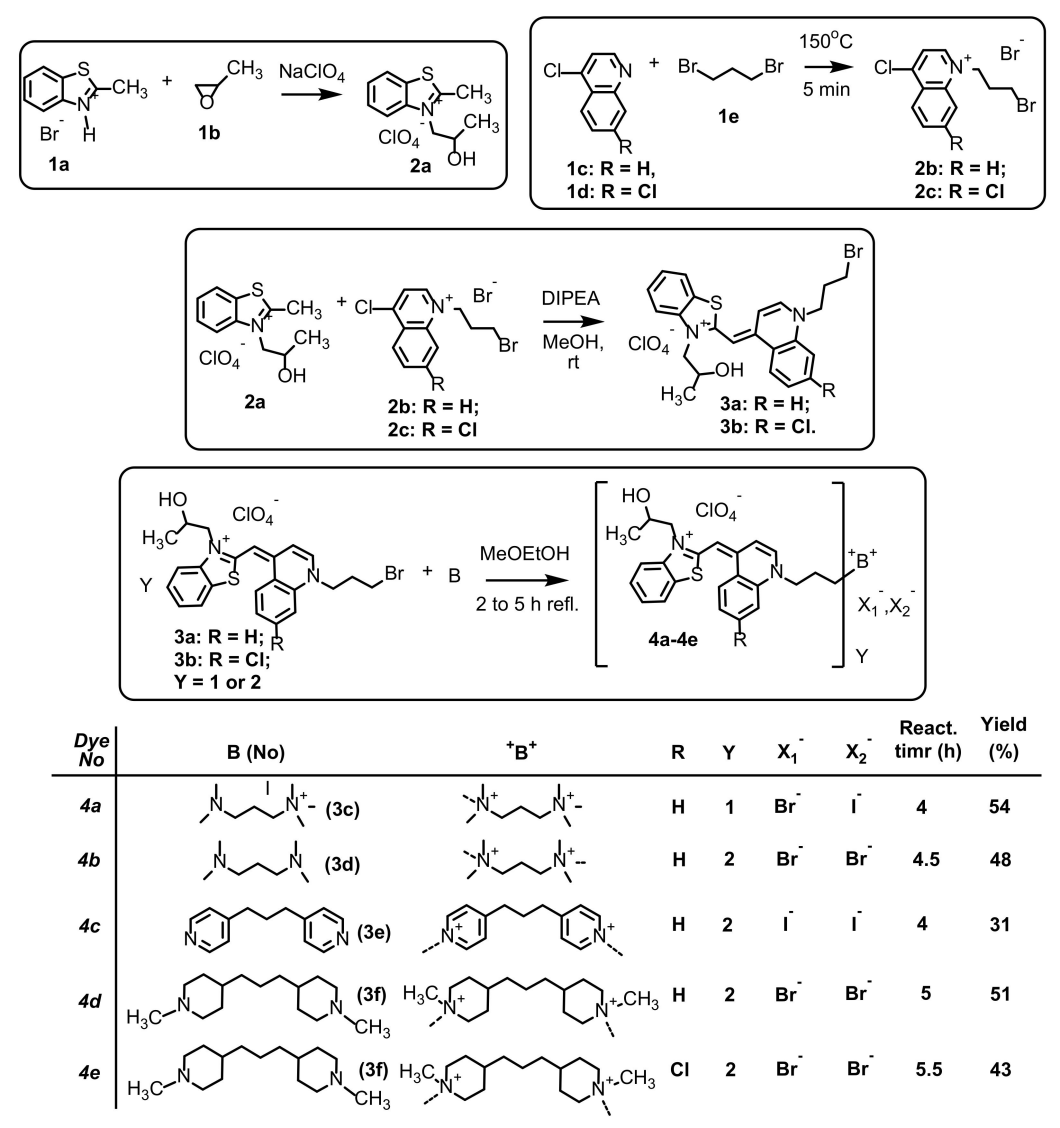
Synthetic conditions, chemical structures and reaction yields of dyes **4a**–**4e**.

In this paper, we present new analogues of monomer TO and homodimer TOTO dyes (Scheme 1, Scheme S1). All new compounds were designed with a bulky 2-hydroxypropyl substituent connected to the benzothiazole nitrogen instead of the more compact methyl group in the molecules of TO and TOTO. The hydroxyl group could enable hydrogen bond formation with the phosphate groups in the nucleic acids chains ensuring various photophysical evinces. Symmetric homodimeric dyes were designed to study the effect of linker flexibility on binding, so three different positively charged linkers between the chromophore units were used: the most flexible trimethylenediammonium (**4b**), the more rigid aromatic trimethylenediammonium bridge (**4c**) and the bulky trimethylenediammonium bridge (**4d** and **4e**) to explore the effect of linker flexibility on the binding. Additionally, **4e** was more hydrophobic because of the chlorine atom on the 7-th position in the quinoline part.

## 2. Materials and Methods

### 2.1. Synthesis

All solvents used in the present work were commercially available (Sigma, HPLC grade). The starting materials **1b** and **1c**–**1e** were commercially available and were used as supplied. The intermediates **1a**, **2a**–**2c**, **3a** and **3b** were prepared by our previously published procedure [15,17]. Melting points were determined on a Kofler apparatus and are uncorrected. NMR spectra were obtained on a Bruker Avance III 500 DRX 600 MHz (Bruker Biospin GmbH, Rheinstetten, Germany) spectrometer in DMSO-*d*6.

#### 2.1.1. NMR Spectroscopy

Prior to the NMR experiments the samples were dissolved in a deuterated DMSO. The characterization of the TO derivatives was conducted on a DRX 400 Avance Bruker NMR spectrometer (Bruker Biospin GmbH, Rheinstetten, Germany) operating at the ^1^H frequency of 400.31 MHz and a ^13^C frequency of 100.66 MHz using a commercial Bruker 5 mm BBO dual channel probe head equipped with z-gradients. A Eurotherm 847 temperature controller with a ± 0.1° precision controlled the temperature. Variable temperature (VT) ^1^H experiments were conducted in the temperature range 25–60 °C with 2 min in each temperature step for equilibration in order to reduce aggregation, resp. increase the spectral resolution. All ^1^H NMR spectra were recorded using a 30° excitation pulse averaging 32 scans with a 2 s recycle delay. The ^13^C NMR spectra were acquired applying power gated decoupling scheme and 1k scans. ^13^C DEPT 135 (distortionless enhancement by polarization transfer) was recorded with 2k scans and 2 s repetition time. ^13^C APT (attached proton test) recorded with 10k scans and 2 s recycle delay experiments was used in some cases to help assign the ^13^C NMR resonances. The sequence was optimized on long-range couplings. The spectrometer was operated by Topspin 1.3 patchlevel 8 (Bruker Biospin GmbH, Rheinstetten, Germany). The ^1^H and ^13^C NMR spectra were referenced to external TMS at 0 ppm. All spectra were evaluated using Mestrec.

#### 2.1.2. Synthesis of Dyes 4a–4e

In a 50 mL round bottom flask equipped with a reflux condenser and an electromagnetic stirrer were added 1 molar equivalent of the starting dye **3a** (or **3b**) and 1 molar equivalent for dye **4a** or 2.2 molar equivalents for the dyes **4b**–**4e** the appropriate basic reagent (B, Scheme 1). The progress of the quaternization (in case of dye **4a**) or bisquaternization in case of dyes **4b**–**4e** was monitored by TLC.

**4a**: Dark red amorphous solid, Mp > 300 °C. Yield 54%. ^1^H-NMR (δ(ppm), 50 °C, 2 min): 1.34 d (3H, CH_3_, ^3^J_HH_ = 6.2 Hz), 2.19–2.22 m (2H, CH_2_), 2.28-2.34 m (2H, CH_2_), 3.09 s (6H, N^+^(CH_3_)_2_), 3.12 s (9H, N^+^(CH_3_)_3_), 3.29 t (2H, N^+^CH_2_, ^3^J_HH_ = 8.3 Hz), 3.54 t (4H, N^+^CH_2_, ^3^J_HH_ = 7.9 Hz), 4.15–4.29 brs (2H, N^+^CH_2_), 4.50 d (1H, N^+^CH, ^3^J_HH_ = 7.9 Hz), 4.60 d (1H, N^+^CH, ^3^J_HH_ = 7.8 Hz), 4.78–4.81 m (1H, CH), 5.19 brs (2H, N^+^CH_2_,) 7.06 s (1H, CH), 7.14 s (1H, CH), 7.77 dd (1H, CH, ^3^J_HH_ = 7.2 Hz), 7.82 dd (1H, CH, ^3^J_HH_ = 8.5 Hz), 8.06 d (1H, ^3^J_HH_ = 7.8 Hz), 8.19 d (1H, ^3^J_HH_ = 8.7 Hz), 8.56 d (1H, ^3^J_HH_ = 6.0 Hz), 8.68 d (1H, ^3^J_HH_ = 8.5 Hz). ^13^C-NMR: 16.65, 21.04, 22.36, 22.39, 50.38, 50.44, 50.70, 51.48, 52.54, 53.19, 53.22, 59.87, 60.52, 60.76, 61.76, 61.79, 64.40, 64.45, 89.27, 89.28, 107.74, 114.11, 117.87, 122.71, 123.86, 123.97, 124.18, 124.54, 124.69, 125.71, 126.85, 128.03, 133.31, 137.06, 140.75, 144.02, 148.28, 148.43, 161.17. ^13^C-NMR DEPT 135 (CH, CH_3_ positive, CH_2_—negative): 16.40 CH_2_, 20.79 CH_3_, 22.14 CH_2_, 50.19 CH_3_, 50.44 CH_2_, 52.29 CH_3_, 52.97 CH_2_, 52.62 CH_2_, 62.27 CH_2_, 61.50 CH_2_, 64.20 CH, 89.03 CH, 107.48 CH, 113.85 CH, 117.62 CH, 122.45 CH, 124.44 CH, 125.46 CH, 126.59 CH, 127.78 CH, 143.77 CH. Mw = 828.04. Chemical Formula: C_31_H_45_BrClIN_4_O_5_S. Elemental analysis: calc.; C 44.97; H 5.48; N 6.77; found: C 44.21; H 5.40; N 6.74.

**4b**: Dark red amorphous solid, Mp > 300 °C. Yield 48%. ^1^H-NMR (δ(ppm), 50 °C, 2 min): 1.34 d (6H, CH_3_, ^3^J_HH_ = 6.2 Hz), 1.66–1.70 m (6H, CH_2_), 1.93 brs (4H, N^+^CH_2_), 3.12 brs (6H, N^+^CH_3_), 3.16 brs (6H, N^+^CH_3_), 3.34 brs (4H, N^+^CH_2_), 4.24 brs (4H, N^+^CH_2_), 4.66 t (4H, N^+^CH_2_, ^3^J_HH_ = 6.9 Hz), 4.78 d (2H, N^+^CH, ^3^J_HH_ = 7.0 Hz), 5.17 d (2H, N^+^CH, ^3^J_HH_ = 7.1 Hz), 5.41–5.62 m (2H, CH), 7.06 s (2H, CH), 7.11 d (2H, CH, ^3^J_HH_ = 9.0 Hz), 7.41 d (2H, CH, ^3^J_HH_ = 7.04 Hz), 7.44 s (2H, CH, ^3^J_HH_ = 7.29 Hz), 7.51 dd (2H, CH, ^3^J_HH_ = 7.04 Hz), 7.60 d (2H, CH, ^3^J_HH_ = 8.5 Hz), 7.62 d (2H, CH, ^3^J_HH_ = 8.0 Hz), 7.67 d (2H, CH, ^3^J_HH_ = 9.0 Hz), 7.71 dd (2H, CH, ^3^J_HH_ = 7.1 Hz), 7.96 d (2H, ^3^J_HH_ = 7.5 Hz), 8.01 dd (2H, ^3^J_HH_ = 8.3 Hz), 8.57 d (2H, CH, ^3^J_HH_ = 7.0 Hz), 8.63 dd (2H, CH, ^3^J_HH_ = 8.8 Hz). ^13^C-NMR: 19.37, 20.44, 20.83, 21.05, 41.53, 50.56, 51.60, 52.25, 53.18, 56.62, 57.44, 64.37, 73.07, 79.83, 107.77, 108.23, 113,86, 114.06, 117.26, 117.32, 117.92, 117.98, 121.38, 122.66, 122.69, 123.73, 123.82, 123.93, 124.17, 124.28, 124.41, 124.65, 125.68, 126.68, 128.01, 129.25, 129.28, 129.35, 129.41, 131.24, 133.01, 133.16, 133.34, 133.53, 136.06, 136.11, 136.32, 140.72, 141.81, 144.07, 148.39, 165.34, 165.37. ^13^C-NMR DEPT 135 (CH, CH_3_ positive, CH_2_—negative): 19.11 CH_3_, 22.12 CH_2_, 28.07 CH_2_, 51.80 N^+^CH_2_, 52.00 N^+^CH_2_, 54.96 N^+^CH_2_, 56.40 N^+^CH_2_, 64.17 CHOH, 79.62 CH, 107.50 CH, 108.00 CH, 113.70 CH, 117.42 CH, 124.37 CH, 126.46 CH, 128.90 CH, 130.37 CH, 132.90 CH, 141.56 CH. Mw = 1241.97. Chemical Formula: C_53_H_66_Br_2_Cl_2_N_6_O_10_S_2_. Elemental analysis: calc.; C 51.26; H 5.36; N 6.77; found: C 49.01; H 5.33; N 6.73.

**4c**: Dark red amorphous solid, Mp > 300 °C. Yield 31%. ^1^H-NMR (δ(ppm), 50 °C, 2 min): 1.32 d (6H, CH_3_, ^3^J_HH_ = 6.2 Hz), 2.04–2.08 m (2H, CH_2_), 2.57 t (2H, N^+^CH_2_, ^3^J_HH_ = 6.7 Hz), 2.93 t (2H, N^+^CH_2_, ^3^J_HH_ = 7.4 Hz), 4.21–4.25 m (4H, CH), 4.54 d (2H, ^3^J_HH_ = 5.8 Hz), 4.66-4.75 m (4H, N^+^CH_2_), 7.08 s (2H, CH), 7.39–7.48 m (2H, CH), 7.58-7.61 m (2H, CH), 7.75-7.77 m (2H, CH), 7.96–7.99 m (2H, CH), 8.47 d (2H, CH, ^3^J_HH_ = 7.2 Hz), 8.60 d (2H, CH, ^3^J_HH_ = 8.3 Hz), 8.89 d (2H, CH, ^3^J_HH_ = 8.3 Hz). ^13^C-NMR: 21.04, 22.39, 50.44, 52.54, 53.22, 59.87, 60.52, 60.76, 61.79, 64.45, 89.28, 107.74, 114.11, 117.87, 122.71, 123.86, 123.97, 124.18, 124.54, 124.69, 125.71, 126.85, 128.03, 133.31, 137.06, 140.75, 144.02, 148.28, 148.43, 161.17. ^13^C-NMR DEPT 135 (CH, CH_3_ positive, CH_2_—negative): 20.73 CH_3_, 29.81 CH_2_, 33.65 CH_2_, 50.66 N^+^CH_2_, 52.87 CH_2_, 57.04 N^+^CH_2_, 64.20 N^+^CH_2_, 88.85 CH, 107.56 CH, 113.68 CH, 117.56 CH, 122.38 CH, 124.44 CH, 125.29 CH, 126.63 CH, 127.42 CH, 127.82 CH, 133.04 CH, 143.58 CH, 143.68 CH. Mw = 1404.01. Chemical Formula: C_59_H_62_Cl_2_I_2_N_6_O_10_S_2_. Elemental analysis: calc.; C 50.47; H 4.45; N 5.99; found: C 50.49; H 4.31; N 5.72.

**4d**: Dark red amorphous solid, Mp > 300 °C. Yield 51%. ^1^H-NMR (δ(ppm), 50 °C, 2 min): 1.30–1.31 m (2H, CH_2_), 1.34 d (6H, CH_3_, ^3^J_HH_ = 6.0 Hz), 1.60-1.65 m (4H, CH_2_), 1.66–1.71 m (4H, CH_2_), 2.06–2.13 m (2H, CH_2_), 3.10–3.28 m (4H, CH_2_), 4.22–4.28 m (4H, CH_2_), 4.59–4.70 m (4H, N^+^CH_2_), 4.87–4.97 m (4H, N^+^CH_2_), 5.18 d (2H, N^+^CH, ^3^J_HH_ = 6.0 Hz), 5.39–5.41 m (2H, CH), 5.48–5.51 m (2H, N^+^CH), 6.71 s (2H, CH), 7.43–7.44 m (2H, CH), 7.60–7.66 m (2H, CH), 7.97–8.01 m (2H, CH), 8.27–8.38 m (2H, CH), 8.56–8.61 m (2H, CH). ^13^C-NMR: 19.78, 20.92, 21.54, 25.88, 27.91, 28.58, 51.27, 53.36, 55.68, 60.56, 64.92, 73.44, 79.94, 80.37, 89.45, 109.82, 114.44, 118.29, 123.12, 125.19, 126.19, 127.33, 129.34, 135.47, 135.88, 137.54, 141.22, 144.51, 151.87, 161.26, 166.10. Mw = 1350.16. Chemical Formula: C_61_H_78_Br_2_Cl_2_N_6_O_10_S_2_. Elemental analysis: calc. C 54.27; H 5.82; N 6.22; found C 53.29; H 5.38; N 5.95.

**4e**: Dark red amorphous solid, Mp > 300 °C. Yield 51%. ^1^H-NMR (δ(ppm), 50 °C, 2 min): 1.22–1.31 m (8H, 6CH_3_ + 2CH_2_), 1.61–1.68 m (4H, CH_2_), 2.28–2.31 brs (2H, CH_2_), 3.20–3.24 m (8H, CH_2_), 4.16–4.19 brs (4H, N^+^CH_2_), 4.50 t (4H, N^+^CH_2_, ^3^J_HH_ = 5.2 Hz), 4.51–4.58 m (4H, N^+^CH_2_), 4.73 t (4H, N^+^CH_2_, ^3^J_HH_ = 6.3 Hz), 5.22–5.43 brs (4H, N^+^CH_2_), 5.41–5.44 m (1H, CH), 5.48–5.52 m (1H, CH), 7.26–7.28 m (4H, CH), 7.55–7.61 m (2H, CH), 7.71 brs (2H, CH), 7.99 dd (2H, CH, ^3^J_HH_ = 7.0 Hz), 8.05 dd (2H, CH, ^3^J_HH_ = 8.3 Hz), 8.25 s (2H, CH), 8.43 dd (2H, CH, ^3^J_HH_ = 5.5 Hz), 8.48 d (2H, CH, ^3^J_HH_ = 7.1 Hz), 8.58 d (2H, CH, ^3^J_HH_ = 7.1 Hz). ^13^C-NMR: 21.48, 51.85, 53.60, 53.70, 64.94, 90.08, 90.13, 108.42, 114.71, 117.62, 120.52, 123.12, 123.21, 123.31, 123.58, 124.52, 125.31, 127.32, 127.67, 128.02, 128.12, 128.17, 128.55, 130.07, 138.46, 138.69, 141.19, 148.07, 148.14, 148.23, 161.94. ^13^C-NMR DEPT: 16.57 CH_2_, 21.49 CH_3_, 27.61 CH_2_, 27.79 CH_2_, 28.26 CH_2_, 39.82 CH_2_, 51.41 N^+^CH_2_, 51.85 N^+^CH_2_, 53.58 N^+^CH_3_, 53.62 N^+^CH_3_, 64.92 N^+^CH_3_, 90.67 CH, 108.28 CH, 114.69 CH, 117.61 CH, 120.46 CH, 123.01 CH, 125.29 CH, 127.29 CH, 128.013 CH, 128.51 CH, 130.04 CH. Mw = 1419.04. Chemical Formula: C_61_H_76_Br_2_Cl_2_N_6_O_10_S_2_. Elemental analysis: calc.; C 51.63; H 5.40; N 5.92; found: C 51.55; H 5.42; N 5.93.

### 2.2. Study of DNA/RNA Interactions

#### 2.2.1. General Procedures

The electronic absorption spectra of newly prepared compounds, UV–Vis titration and thermal melting experiments were measured on a Varian Cary 100 Bio spectrometer (Agilent, Santa Clara, CA, United States). Fluorescence spectra were recorded on a Varian Cary Eclipse fluorimeter (Agilent, Santa Clara, CA, United States). CD spectra were recorded on a JASCO J815 spectrophotometer (ABL&E Handels GmbH, Wien, Austria). UV–Vis, fluorescence and CD spectra were recorded using the appropriate 1 cm path quartz cuvettes. Polynucleotides were purchased as noted: calf thymus (*ct*)-DNA, poly dAdT–poly dAdT, poly dGdC–poly dGdC, poly dA–poly dT, poly dG–poly dC, poly rA–poly rU and poly rG–poly rC (Sigma) and dissolved in sodium cacodylate buffer, I = 0.05 mol dm^−3^, pH=7.0. The calf thymus (*ct*-) DNA was additionally sonicated and filtered through a 0.45 µm filter [18]. The polynucleotide concentration was determined spectroscopically and expressed as the concentration of phosphates [19,20,21]. Stock solutions of **4a**–**4e** compounds were prepared by dissolving compounds in H_2_O or DMSO; total DMSO content was below 1% in UV–Vis and below 0.1% in fluorimetric measurements. All measurements were performed in sodium cacodylate buffer, I = 0.05 mol dm^−3^, pH = 7.0. **4a**–**4e** concentrations below 2 × 10^−5^ M were used for the UV–Vis absorbance measurement to avoid intermolecular association.

#### 2.2.2. UV/Vis, CD and Fluorescence Titrations

UV–Vis and fluorimetric titrations were performed by adding portions of polynucleotide solution into the solution of the studied compound. After mixing polynucleotides with studied compounds it was observed that equilibrium was reached in less than 120 s. UV–Vis titrations were measured in wavelengths between 400 and 600 nm. In fluorimetric titrations, the concentration of studied **4a**–**4e** compounds was 1–2 × 10^−7^ M. Although **4a**–**4e** compounds do not show intrinsic fluorescence, they show a significant fluorescence increase upon binding to polynucleotides. The excitation wavelength of λ_exc_ > 400 nm was used to avoid absorption of the excitation light caused by increasing absorbance of the polynucleotide. Emission was collected in the range λ_em_ = 550–650 nm. UV–Vis and fluorescence spectra were collected at r < 0.3 (r = [compound]/[polynucleotide]) to assure one dominant binding mode. Titration data obtained for ds-DNA and ds-RNA were processed using the Scatchard equation [22,23]. Calculations mostly gave values of ratio *n* = 0.2 ± 0.05, but for easier comparison, all Ks values were recalculated for fixed *n* = 0.2. Values for Ks have satisfactory correlation coefficients (>0.98). In the Scatchard equation [22,23] values of apparent stability constant (Ks) and ratio (*n* = [bound compound]/[polynucleotide]) are highly mutually dependent and a similar quality of fitting calculated to experimental data is obtained for ± 20% variation for Ks and *n*; this variation can be considered as an estimation of the errors for the given apparent binding constants. CD experiments were performed by adding portions of **4a**–**4e** compound stock solution into the solution of the polynucleotide (c = 1–2 × 10^−5^ mol dm^−3^). Examined **4a**–**4e** compounds were achiral and therefore do not possess intrinsic CD spectra. CD spectra were recorded with a scanning speed of 200 nm/min. Buffer background was subtracted from each spectrum, while each spectra was the result of five accumulations.

#### 2.2.3. Thermal Melting Experiments

Thermal melting curves for ds-DNA, ds-RNA and their complexes with studied compounds were determined by following the absorption change at 260 nm as a function of temperature. The absorbance scale was normalized. Tm values are the midpoints of the transition curves determined from the maximum of the first derivative and checked graphically by the tangent method. The ΔTm values were calculated by subtracting Tm of the free nucleic acid from Tm of the complex. Every ΔTm value reported here was the average of at least two measurements. The error in ΔTm was ± 0.5 °C.

### 2.3. Evaluation of the Antiproliferative Effect

The growth inhibition activity was assessed according to the slightly modified procedure performed at the National Cancer Institute, Developmental Therapeutics Program [24].

#### 2.3.1. Cell Lines and Culturing

Examined **4a**–**4e** compounds were dissolved in DMSO (1 × 10^−2^ mol dm^−3^). The experiments were carried out on 4 human cell lines. The cell lines purchased from the American Type Culture Collection (ATCC, Poland) were used: H460 (lung carcinoma, large cell lung cancer (ATCC^®^HTB-177™)), MCF-7 (breast adenocarcinoma, ATCC^®^ HTB-22™), HCT116 (colorectal carcinoma ATCC^®^ CCL-247™) and HEK 293 (embryonic kidney cells); ATCC^®^ CRL-1573™).

Cells were cultured as monolayers and maintained in Dulbecco’s modified Eagle medium (DMEM), supplemented with 10% fetal bovine serum (FBS), 2 mM of L-glutamine, 100 U/mL of penicillin and 100 μg/mL of streptomycin in a humidified atmosphere with 5% CO_2_ at 37 °C.

#### 2.3.2. Proliferation Assays

The panel cell lines were inoculated onto a series of standard 96-well microtiter plates on day 0, at 1 × 10^4^ to 3 × 10^4^ cells/mL, depending on the doubling times of a specific cell line. Test agents were then added in five 10-fold dilutions (10^−8^ to 10^−4^ M) and incubated for a further 72 h. Working dilutions were freshly prepared on the day of testing. The solvent was also tested for eventual inhibitory activity by adjusting its concentration to be the same as in working concentrations. After 72 h of incubation, the cell growth rate was evaluated by performing the MTT assay, which detects dehydrogenase activity in viable cells [25]. The MTT cell proliferation assay is a colorimetric assay system, which measures the reduction of a tetrazolium component (MTT) into an insoluble formazan product by the mitochondria of viable cells. For this purpose, the substance treated medium was discarded and MTT was added to each well at a concentration of 20 μg/40 μL. After four hours of incubation, the precipitates were dissolved in 160 μL of dimethyl-sulfoxide (DMSO). The absorbance (OD, optical density) was measured on a microplate reader at 570 nm. The absorbance is directly proportional to the cell viability. The percentage of growth (PG) of the cell lines was calculated according to one or the other of the following two expressions:

If (mean OD_test_ − mean OD_tzero_) ≥ 0 then

PG = 100 × (mean OD_test_ − mean OD_tzero_)/(mean OD_ctrl_ − meanOD_tzero_).

If (mean OD_test_ − mean OD_tzero_) < 0 then:

PG = 100 × (mean OD_test_ − mean OD_tzero_)/OD_tzero_.

where:

Mean OD_tzero_ = the average of optical density measurements before exposure of cells to the test compound.

Mean OD_test_ = the average of optical density measurements after the desired period.

Mean OD_ctrl_ = the average of optical density measurements after the desired period with no exposure of cells to the test compound.

Each test point was performed in quadruplicate in two individual experiments. The results were expressed as GI_50_, a concentration necessary for 50% inhibition. Each result was a mean value from at least two separate experiments. The GI_50_ values for each compound were calculated from dose–response curves using the linear regression analysis by fitting the test concentrations that give PG values above and below the respective reference value (e.g., 50 for GI_50_). Therefore, a “real” value for any of the response parameters is obtained only if at least one of the tested drug concentrations falls above, and likewise at least one falls below the respective reference value. If however, for a given cell line all of the tested concentrations produce PGs exceeding the respective reference level of effect (e.g., PG value of 50), then the highest tested concentration is assigned as the default value. In the screening data report, that default value is preceded by a “>” sign.

#### 2.3.3. Confocal Microscopy

H460 cells were treated with 1 µM of 4c compound for 60 min at 37 °C, 5% CO_2_ and incubated with 100 nM MitoTracker^®^ (Thermo Fisher Scientific Inc.) and 20 mM Hoechst 33342 solution for 20 min at 37 °C (Thermo Fisher Scientific Inc.). Cells were analyzed by confocal microscopy (Leica SP8 X confocal microscope, Wetzlar, Germany). Excitation and emission wavelengths were as followed: λ_exc_ = 484 nm and λ_em_ = 538 nm for 4c; λ_exc_ = 644 nm and λ_em_ = 665 nm for MitoTracker^®^ Deep Red; λ_exc_ = 361 nm and λ_em_ = 497 nm for Hoechst 33342.

## 3. Results and Discussion

### 3.1. Synthesis

The starting compounds 2-methylbenzo [*d*]thiazol-3-ium bromide **1a**, 3-(2-hydroxypropyl)-2-methylbenzo [*d*]thiazol-3-ium perchlorate **2a**, 1-(3-bromopropyl)-4-chloroquinolin-1-ium bromide **2b** and 1-(3-bromopropyl)-4,7-dichloroquinolin-1-ium bromide **2c** and the initial dyes (*E*)-2-((1-(3-bromopropyl)quinolin-4(1*H*)-ylidene)methyl)-3-(2-hydroxypropyl)benzo [*d*]thiazol-3-ium perchlorate **3a** and (*E*)-2-((1-(3-bromopropyl)-7-chloroquinolin-4(1*H*)-ylidene)methyl)-3-(2-hydroxypropyl)benzo [*d*]thiazol-3-ium perchlorate 3b were prepared according to the procedures previously published by our group (Scheme 1) [15,16,17].

The quaternization of 2-((1-(3-bromopropyl)quinolin-4(1H)-ylidene)methyl)-3-(2-hydroxypropyl)benzo [d]thiazol-3-ium perchlorate **3a** with 3-(dimethylamino)-N,N,N-trimethylpropan-1-aminium iodide **3c** for the preparation of dye 2-((1-(3-(dimethyl(3-(trimethylammonio)propyl)ammonio)propyl)quinolin-4(1H)-ylidene)methyl)-3-(2-hydroxypropyl)benzo [d]thiazol-3-ium diiodide perchlorate **4a** was performed in 2-methoxyethanol. Similarly, the bisquaternization of **3a** and **3b** (Scheme 1) with N,N,N^’^,N^’^-tetramethyl-1,3-propanediamine **3d**, 1,3-di(4-pyridyl)propane **3e** and 4,4′-Trimethylenebis(1-methylpiperidine) **3f** were carried out in the same solvent and the reaction progress was followed by TLC monitoring. The reaction yields are from moderate to relatively low probably because of reactions of the Hoffmann elimination illustrated as mobile red spots on the TLC chromatogram. The chemical structures were proved by ^1^H-NMR, ^13^C-NMR and DEPT at different temperatures because of the solubility issues and elemental analysis. In the ^1^H-NMR spectra of the final dyes, the most characteristic are the ABX-protons from the 2-hydroxypropyl fragment (N^+^CH_2_CHOH) appearing as multiplets in the range 4.5–5.7 ppm (Materials and Methods). The NMR spectra were measured at different temperatures to increase the solubility of the dyes and avoiding aggregate formation. The best resolution was obtained at 50 °C.

### 3.2. Characterization of the Photophysical Properties of Dyes **4a**–**4e** in the Aqueous Medium

The compounds **4a**–**4c** were moderately soluble in water (about *c* = 1 × 10^−4^ mol dm^−3^), while **4d** and **4e** stock solutions were prepared in DMSO (about *c* = 5 × 10^−3^ mol dm^−3^). Thiazole orange (TO) and its homodimer TOTO had a strong tendency to aggregate in an aqueous solution and also on biological surfaces [26,27,28]. Oppositely, absorbencies of aqueous solutions of all studied compounds were proportional to their concentrations up to *c* = 2–4 × 10^−5^ mol dm^−3^, indicating that there was no significant intermolecular stacking, which would result in hypochromic effects. UV–Vis spectra of aqueous solutions of all examined compounds did not change significantly during heating up to 90 °C; all minor changes were reversible after cooling back to room temperature. Absorption maxima and corresponding molar extinction coefficients (ε) were given in the Supplementary Materials, Figure S1. TOTO (thiazole orange homodimer) showed a strong absorption peak at 480 nm and a somewhat weaker absorption peak at 512 nm. [9] Many monomethine cyanines manifested multiple bands, which were attributed to different vibronic bands of the same electronic transition [29]. New homodimer compounds **4b**–**4d**, analogues of TOTO, showed very similar absorption spectra: the strong signal at 480 ± 4 nm and the weaker signal at 515 ± 5 nm (Table S1, Figure S1) indicating the formation of a stable intramolecular H-aggregate. Aqueous solutions of compounds **4a**–**4e** at concentrations c = 1 × 10^−6^ mol dm^−3^ did not show any fluorescence even at the highest sensitivity of the instrument.

### 3.3. Interactions of **4a**–**4e** with ds-Polynucleotides in an Aqueous Medium

Synthetic ds-polynucleotides with different base compositions and/or types of helical structures were chosen for the assay to examine the binding of dyes to different types of DNA/RNA binding sites (intercalation or groove binding). For example, poly dAdT–poly dAdT represented the typical B-helical structure (having an ideal minor groove for small molecule binding), while poly rA–poly rU was a classic A-helical structure with a major groove more suited for small molecules (Table S2).

#### 3.3.1. Thermal Melting Studies

Non-covalent binding of small molecules to ds-polynucleotides usually has a certain effect on the thermal stability of helices thus giving different T_m_ values (temperature of dissociation of the double-stranded helix into two single-stranded polynucleotides) [30]. Thermal denaturation experiments (Table 1, Figures S2–S9) revealed that all examined compounds stabilized the double helices of the examined DNA polynucleotides.

Monomer compound **4a** showed strong stabilization of both DNA and RNA double helixes. It also demonstrated the strong nonlinear dependence of ΔT_m_ values on the ratio *r* ([compound]/[polynucleotide]), suggesting saturation of binding sites at *r* ≈ 0.2 for DNA and ***r*** ≈ 0.15 for RNA (Figures S2–S9).

Surprisingly, the foreseen synergistic effect in dimer failed, so dimer compounds **4b, 4d** and **4e** showed significantly weaker stabilization of DNA polynucleotides than monomer **4a** and negligible/no stabilization of RNA. The weaker stabilization of ds-polynucleotides by **4d** and **4e** was probably the consequence of their binding/aggregation within polynucleotide hydrophobic grooves with a weaker impact on double helix stability. Additionally, no difference in ΔT_m_ values for **4d** and **4e** was observed, indicating no influence of the chloro-substituent to double helix stabilization. Considering the A-helix structure of poly rA–poly rU, the lack of RNA stabilization suggested no intercalation or only partial intercalation of examined dyes.

Among all dimer compounds, **4c** with an aromatic di-pyridinium linker between dye units showed the strongest ds-DNA stabilization, which was similar as obtained for the monomer in the case of *ct*-DNA and even stronger in the case of poly dAdT–poly dAdT (Table 1). Surprisingly, **4c** stabilized poly rA–poly rU at lower r ratios (r < 0.2) but strongly destabilized it at higher ratios (r ≥ 0.2). High stabilizations observed for poly rA–poly rU caused by **4c** could indicate intercalation and possible bisintercalation as a main binding mode. **4c** might have stabilized polynucleotide by intercalation according to the neighbor exclusion principle [31,32] at lower *r* ratios (r < 0.2). At higher ratios, all intercalation binding sites were occupied, while the RNA groove was not suitable for binding. Consequently, ds-polynucleotide was destabilized. This destabilization could cause RNA uncoiling or formation of the colloid particles upon complex aggregation followed by absorbance increase.

#### 3.3.2. Spectrophotometric Titrations

**Figure 1:**
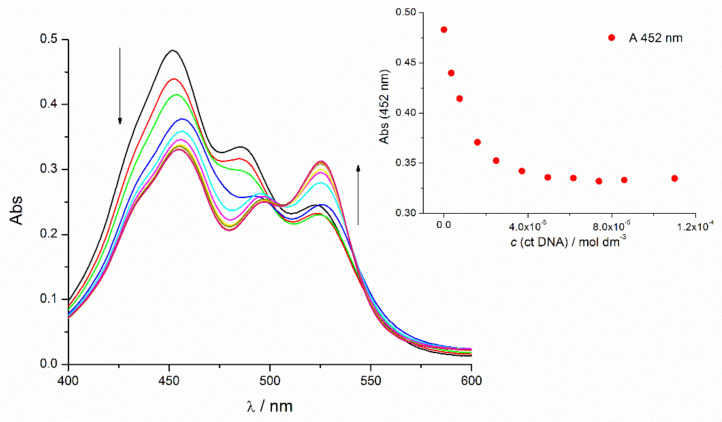
UV/Vis titration of **4e** (*c* = 9 × 10^−6^ mol dm^−3^, sodium cacodylate buffer, pH = 7, *I* = 0.05 mol dm^−3^) with ct-DNA; inset: changes in the UV/Vis spectra of **4e** at λ_max_ = 452 nm.

The addition of *ct*-DNA to the solution of **4a** caused the hypochromic effect typical for the monomeric **TO** and bathochromic shift of the compound’s UV–Vis spectra. As a result of the titration of dimer compounds **4b**–**4e** with *ct*-DNA, the hypochromic effect and the small bathochromic shift occurred for the signal at 480 ± 4 nm indicating an interaction of the *ct-*DNA and intramolecular H-aggregate. The absorption peak at 515 ± 5 nm increased significantly, with a bathochromic shift combined with a hyperchromic effect typical for **TO** monomer intercalation (Figure 1, Figure S10, Table S3). The longer wavelength absorption maximum at 525 nm appeared increasing ratiometrically with an increase of *ct-*DNA concentration. This systematic deviation of the isosbestic points in the Vis region (e.g., about 500 nm, Figure 1) and pointed out more spectroscopically active species and more than one dominant binding mode: probably J-aggregation on the *ct*-DNA surface.

**Figure 2:**
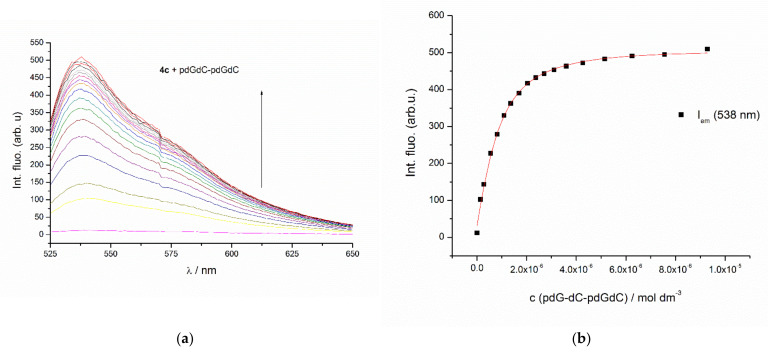
(**a**) Fluorimetric titration (λ_exc_ = 488 nm) of **4c** (c = 1.3 × 10^−7^ mol dm^−3^) with poly dGdC–poly dGdC; (**b**) Experimental (●) and calculated (–) fluorescence intensities of **4c** at λ_em_ = 538 nm.

Cyanine dyes were usually non-fluorescent in water due to the non-radiative torsional angle rotation and vibrational relaxation about the semi-single bond. It was characteristic for cyanines to increase their fluorescence emission significantly upon binding to the protein or polynucleotide, due to restricted internal rotation and conformational mobility of the dye within the binding site [8]. The H-aggregates of the monomeric and homodimeric asymmetric monomethine cyanine dyes also demonstrated insignificant intrinsic fluorescence in water solutions caused by structure rigidity. Surprisingly compounds **4a**–**4e** did not show any intrinsic fluorescence, but their complexes with polynucleotides showed a notable one (Figure 2, Figures S12–S18)—a behavior typical for the acridine orange analogues (EvaGreen for example) [33]. As a result of fluorimetric titrations of **4a**–**4e** with natural (*ct*-DNA) and synthetic double-stranded DNA and RNA, apparent stability constants of dye-DNA (RNA) complexes were calculated using the Scatchard equation [22,23]. All examined compounds showed a strong, micromolar and submicromolar affinities for most examined ds-polynucleotides (Table 2).

**Figure 3:**
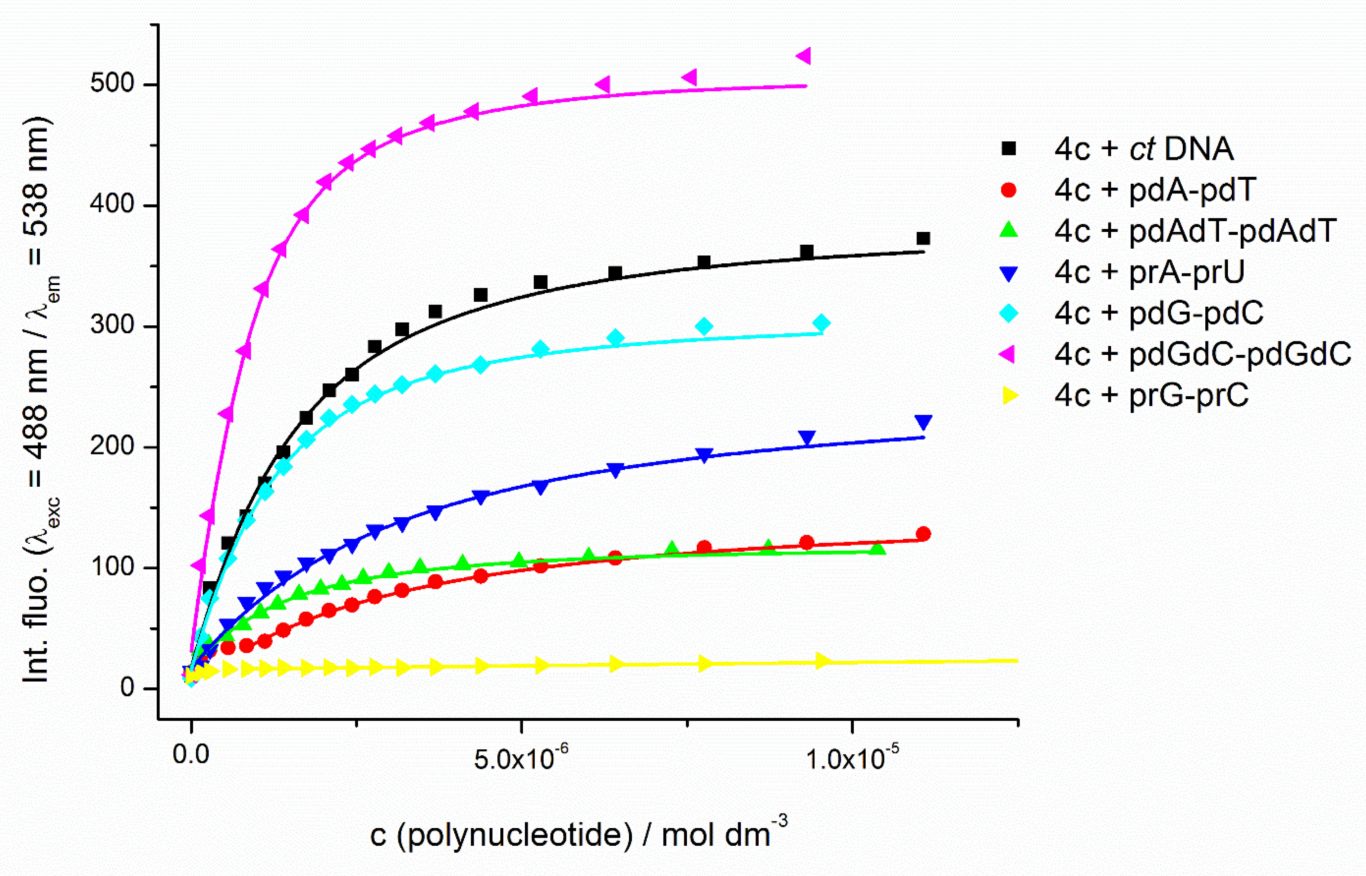
Changes of the fluorescence emission of **4c** (c = 1.48 × 10^−7^ mol dm^−3^) upon addition of *ds*-polynucleotides at pH 7 (Na-cacodylate buffer, pH = 7.0, *I*=0.05 M; λexc = 488 nm/λem = 538 nm; excitation slit of 5 nm and emission slit of 10 nm).

It was interesting to note no significant differences in apparent stability constants for monomer **4a** and dimer–polynucleotide complexes occurred. These similar constants probably excluded bisintercalation of dimer dyes as their dominant binding mode, because bisintercalation would cause stability constants at least one order higher compared to the monomer dye. Additionally, in most cases, affinities for different ds-polynucleotides were very similar. However, there were a few exceptions. All compounds showed a more than one magnitude lower stability constant for poly rG–poly dC than for other polynucleotides, including RNA poly rA–poly rU. Further, **TOTO** structural analogue **4b** revealed one magnitude higher affinity for RNA poly rA–poly rU compared to other dimer dyes. Dimer molecules with the di-pyridinium (**4c**) and di-piperidinium linker between cyanine units (**4d** and **4e**) showed higher stability constants for GC-DNA-polynucleotides over the AT-DNA-polynucleotide (Table 2). All dimers also revealed a significant fluorescence increase upon binding to GC sequences (Figure 3), with a noticeable higher response for GC than AT base pairs, which was probably influenced by interactions of the positively charged linker with GC base pairs positioned within the polynucleotide groove.

#### 3.3.3. Circular Dichroism (CD) Experiments

CD spectroscopy was performed to observe conformational changes of polynucleotide secondary structure induced by the complexation of cyanine dyes. It is important to note that **4a**–**4e** were achiral and without intrinsic CD spectra. It was well known that the binding of a small molecule to chiral polynucleotide could induce the CD signal (ICD) of achiral dye. Induced CD spectra pointed specific, uniform orientation of dye concerning the helical axis and pseudo dyad axis, indicating the mode of interaction (intercalation, groove binding, agglomeration, etc.) [33,34,35,36,37].

**Figure 4:**
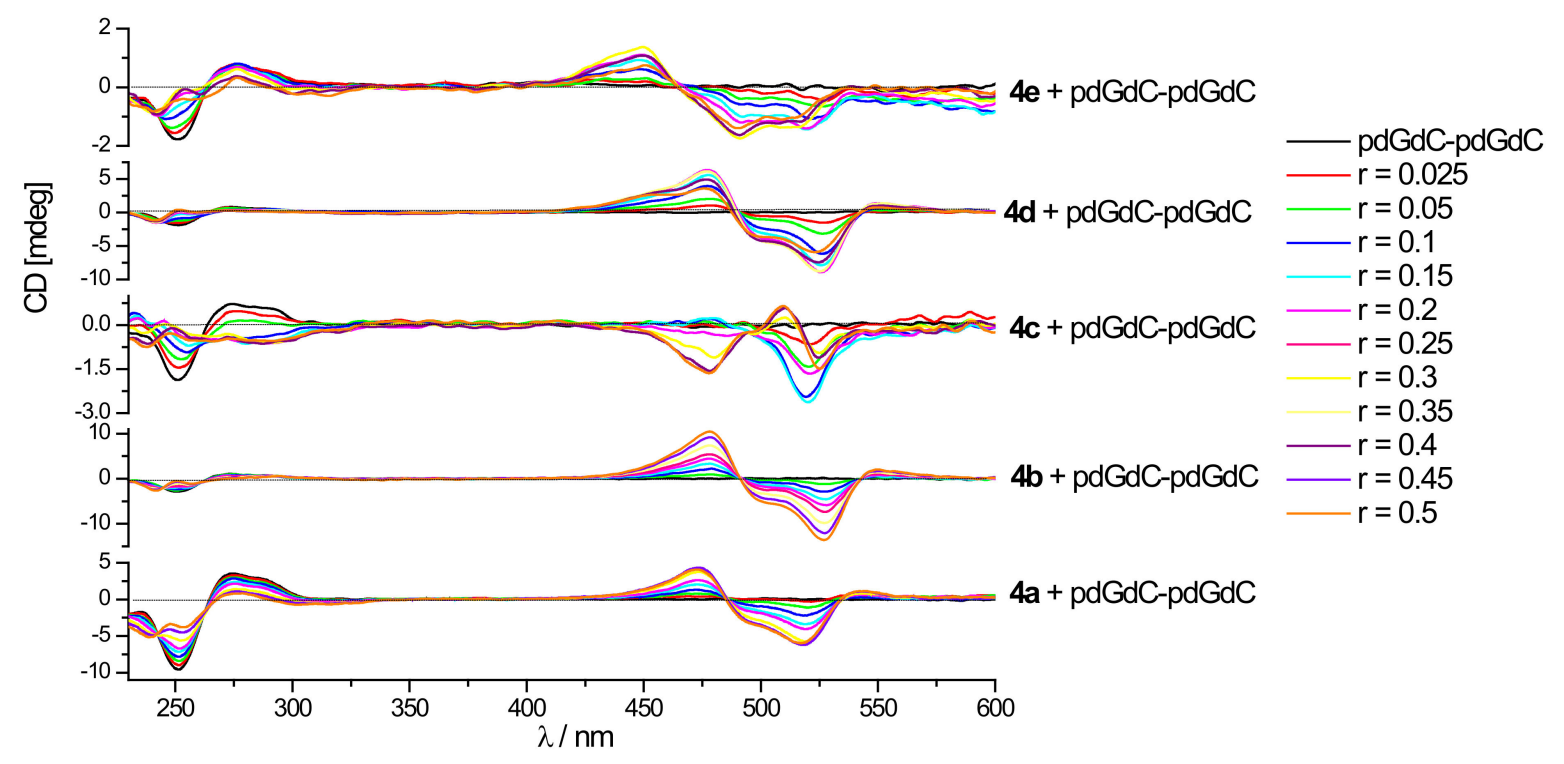
Changes in the CD spectrum of poly dGdC–poly dGdC (*c* = 1–2 × 10^-5^ mol dm^-3^) upon addition of **4a**–**4e** compounds at different molar ratios *r* = [compound] / [polynucleotide], pH = 7.0, sodium cacodylate buffer and *I* = 0.05 mol dm^-3^.

The addition of cyanine dyes to polynucleotide solutions decreased the CD spectra of all examined polynucleotides (240–300 nm range) and produced intense ICD spectra in the region corresponding to the compound’s absorption (400–550 nm range, Figure 4, Figures S19–S23). A significant decrease of polynucleotide CD spectrum usually indicated impairment of helical chirality due to intercalation of the dye or binding of bulky molecules within the polynucleotide groove [38]. Strongly pronounced non-linear dependence of changes in CD spectra on the ratio *r* pointed toward saturation of dominant binding sites at about r = 0.2 in most of the cases. These *r* values were in good agreement with the non-linear dependence of ΔTm values on the ratio r in thermal melting experiments (Table 1).

These different ICD signals obtained for different polynucleotides were the consequence of the adaptation of both dimer dye and structurally different polynucleotides. For example, DNA B-helices had different dimensions of their deep/narrow minor groove (Table S2) and/or different steric restrictions (e.g., guanine amino groups that were interchangeably spaced within the minor groove of poly dGdC–poly dGdC). Further, RNA A-helices had a shallow minor groove, deep/narrow major groove, etc.

The **TO** analogue, monomer **4a**, mostly gave strong bisignate ICD signals at 470 and 520 nm upon binding, indicating aggregation of the dye within the polynucleotide groove, that was used as a template (minor groove for DNA polynucleotides or major groove for poly rA–poly rU, Table S2). Well-defined isodichroic points in ICD bands suggest only one type of aggregate formed, most likely dimeric. The binding of **4a** to GC RNA caused only a negligible change of the polynucleotide and weak negative ICD (Figure S19).

The **TOTO** analogue, dimer **4b** showed a similar pattern upon binding to all examined DNA: a decrease of the polynucleotide CD signal and very strong bisignate signals at 478 and 525 nm (triple or more times stronger than DNA CD signal) combined with an additional small positive ICD band at 552 nm, which supported uniform aggregation of ligand chromophores along the polynucleotide chiral helix with well-defined supramolecular chirality [38]. Binding to RNA poly rA–poly rU resulted in a small negative ICD that suggested intercalation of the dye, while the change of poly rG–poly rC was negligible (Figure S20). The flexible propylene-ammonium linker of **4b** probably enabled the best fitting into polynucleotides grooves among all examined dyes. This resulted in the uniform binding pattern and rarely strong ICD signals.

Dimer **4c** had an aromatic bispyridinium linker between two cyanine units, which was sterically more demanding, more bulky and rigid than the TOTO polypropylene-ammonium linker. This linker also enabled additional hydrophobic π–π interactions with polynucleotide bases within the groove. It was interesting to note a change of DNA CD spectra upon titration with **4c**: homo DNA polynucleotides strongly decreased their spectra, while alternating polynucleotides, including *ct*-DNA, inverted their CD spectra that demonstrated a significant influence of the **4c** linker and very strong perturbation of polynucleotide helicity (Figure S21). This was a unique CD response among other examined compounds. The ICD spectra of **4c** (Figure 4, Figures S19–S23) showed a rather complicated pattern with systematic deviation from isodichroic points, probably because of different binding modes. *ct*-DNA and alternating AT DNA represented the typical B-helix having a deep and narrow minor groove. Binding of **4c** to these DNA polynucleotides induced a small negative CD band at 525 nm for *r* ≤ 0.1 that agreed well with the intercalation or eventual bisintercalation. With an excess of ligand over polynucleotide binding sites (*r* > 0.2), strong bisignate ICD bands at 480 and 535 nm appeared as a result of uniform aggregation/dimerization within the B helix groove. **4c** showed strong negative ICD signals at 480 and 530 nm upon binding to both alternating and homo GC DNA up to *r* ≤ 0.2 probably related to the intercalation. Bisignate bands showed up at *r* ≥ 0.3. CD titration of RNA with **4c** resulted in a decrease of the polynucleotide signal for both AU and GC RNA. Negative ICD signals at *r* < 0.15 related to intercalation and bisignate signals related to aggregation were much more pronounced with AU than GC RNA.

Dimer dyes **4d** and **4e** possessed an aliphatic bulky di-piperidinium linker, while **4e** had additional chloro-substitution on the quinoline unit. Both of these dyes caused a decrease in the CD spectra of all examined ds-polynucleotides (Figures S22–S23). The piperidine linker of **4d** was more voluminous than the polypropylene-ammonium linker of the closest **TOTO** analogue **4b**. However, their complexes with polynucleotides showed quite a similar CD response and very strong ICD signals, indicating aggregation of the ligand within the chiral axis. CD responses and shapes of spectra and ICD signals of chloro-analogue **4e** obtained in titrations with both GC DNA and both RNA were very similar to **4b** and **4d**. Titrations of ct DNA and both AT DNA with **4d** and **4e** showed a different response. Bisignate ICD signals of **4e** had different shape and intensity compared to **4d**. That observation stressed the importance of the electron–donor chloro-substituent. For both compounds, no negative ICD signals at low r values, characteristic for intercalation were obtained, which was in agreement with no thermal stabilization of RNA.

#### 3.3.4. Discussion of the Spectroscopic Results

Cyanine dyes **TO** and **TOTO** were modified by replacing the benzothiazole methyl group with by hydroxypropyl group. Further, the quinoline unit was substituted with chlorine and the piperidine/pyridine linker was introduced. Our goal was to explore the influence of the additional monomethine cyanine unit, chlorine substituent and steric/electronic properties of the linker on DNA/RNA binding affinities and fluorescence/CD response.

UV–Vis titrations of compounds with ct DNA showed a decrease of the absorption band at 480 ± 4 nm and a strong increase of absorption peak at 515 ± 5 nm, combined with the bathochromic shift of both bands. Bathochromic shifts together with bisignate ICD bands observed in CD titrations, indicated assembling of examined dyes to the supramolecular J-aggregate on the polynucleotide template [39]. We call that photophysical phenomenon a bioaggregachromism [40].

Despite the additional cyanine unit, dimer dyes **4b**–**4e** did not show significantly higher stability constants or thermal stabilizations of the double helix than monomer **4a** that excluded bisintercalation of dimers as a dominant binding mode. For binding of all compounds on RNA, a strong preference for AU over GC RNA was observed, which was apparent from fluorimetric and also CD titrations. Based on thermal melting, fluorimetric titrations and ICD signals, **4b** and **4c** bound to pApU by intercalation and possibly by aggregation within a major groove at a higher dye/DNA ratio. **4d** and **4e** showed no intercalation into poly rA–poly rU. A lack of intercalation in most cases could be a consequence of steric hindrance of the hydroxypropyl group. Among all compounds, **4b** showed the strongest affinities over polynucleotides (Table 2) and particularly strong ICD bands, probably because its flexible polypropylene-ammonium linker accommodated well into polynucleotides grooves and enabled an optimal and uniform interaction of the dye and polynucleotide.

All compounds revealed as bisignate ICD signals of different intensity that implied the formation of J-aggregates of dyes in the unique pattern concerning the polynucleotide chiral axis. Among all compounds, only **4c** showed a strong negative ICD band characteristic for intercalation at very low r values (0.15) that corresponded to an excess of binding sites. Although apparent stability constants for all compounds and DNA polynucleotides were similar, there was a noticeable preference for GC over AT DNA. This recognition was revealed also in the stronger fluorescence response in fluorimetric titrations (Figure 3, Figure S18), stressing the importance of additional possibly hydrogen bonding interactions of GC base pairs with examined dyes. A different pattern (intensity, shape and direction of ICD signals and a decrease of the polynucleotide CD signal) of the CD response induced by examined dyes indicated the influence of small structural differences (Figure 4). **4b** revealed the strongest ICD bands upon binding to polynucleotides. **4c** yielded the strongest change and inversion of alternating GC DNA CD spectra related to the interruption of helical chirality. This observation pointed out the significant role of the rigid, aromatic pyridine linker.

### 3.4. Antiproliferative Effect of Compounds In Vitro

The biological experiments aimed to verify the capability of these DNA/RNA binders to penetrate cells estimated their intracellular location and subcellular targets and evaluated their antiproliferative effect. Based on these experiments, we could identify their further prospective application as either theranostic agents [41] (combining fluorescent probing with antiproliferative action) or as non-cytotoxic dyes suitable for intracellular applications [42,43].

Firstly, **4a**–**4e** compounds were tested for their antiproliferative activity against three human cancer cell lines: HCT 116 (colorectal carcinoma), MCF-7 (breast carcinoma) and H460 (large cell lung cancer), while **4c** was additionally tested against the healthy cell line HEK 293. Examined compounds showed micromolar growth-inhibiting activity against cancer cell lines (Table 3, Figure S24).

**Figure 5:**
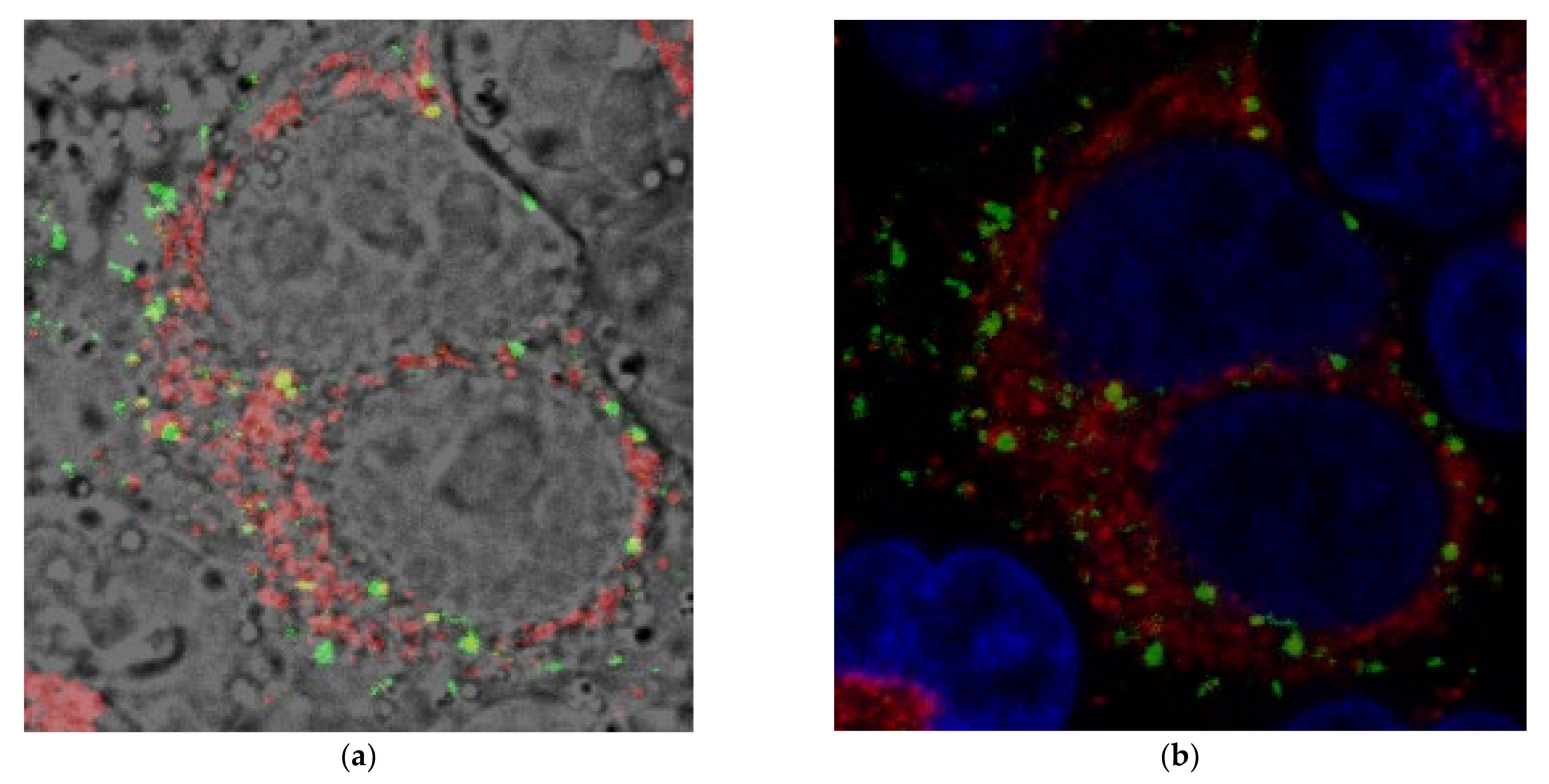
Colocalization of **4c** (green, 1 μM) with MitoTracker^®^ (red) and Hoechst 33342 (blue, nucleus) in H460 cells observed by confocal microscopy (cells were treated with 1 µM of the compound for 60 min at 37 °C and incubated with a 100 nM MitoTracker^®^ and 20 mM Hoechst 33342 solution for 20 min at 37 °C): (**a**) overlay of brightfield with **4c** (green) and MitoTracker^®^ (red); (**b**) overlay of **4c** (green) with Hoechst 33342 (blue, nucleus) and MitoTracker^®^ (red).

Dimer dyes **4b–4e** revealed similar affinities to polynucleotides and also similar antitumor activities compared to monomer **4a**. Generally, compound **4c** (dimer dye with the bipyridine linker) showed the strongest inhibitory potency on a tumor cell growth among all tested compounds, but without selectivity for the certain cell line. Cytotoxic activity of **4c** against the healthy cell line HEK 293 was determined and the GI_50_ value was 20 ±2 μM. This value was approximately 10 times higher compared to tumor cells, revealed lower cytotoxicity for healthy cells and indicated the therapeutic potential of the dye. Since **4c** showed a distinct cytotoxic effect, we studied its cellular uptake and intracellular distribution in H460 cells by confocal microscopy. The **4c** (1 µM) entered live cells within 60 min incubation at 37 °C, 5% CO_2_ (Figure 5). Once in the cells, **4c** accumulated in the cytoplasm, contrary to the expected subcellular localization in DNA-rich organelles, the nucleus or mitochondria, where cytotoxic DNA intercalators usually accumulate. The grain-like distribution of green spots in the vicinity of the nucleus (Figure 5) suggests lysosomal or endosomal vesicles as possible targets. However, detailed biological studies are needed for the accurate determination of the cellular target and the biological mechanism of cytotoxic action.

## 4. Conclusions

New cyanine dyes were synthesized according to previously published procedures and quaternized to afford the monomer and dimer water-soluble dyes. Analogues of the TO and TOTO dye were substituted by the hydroxypropyl on benzothiazole group instead of methyl, while cyanine units were linked by polypropylene-ammonium, dipyridinium or the dipiperidinium bridge. All compounds, dimer dyes and monomers showed a strong fluorescence increase upon binding to polynucleotides and high micromolar and submicromolar affinities toward DNA ds-polynucleotides and poly rA–poly rU.

Higher affinities and a fluorimetric response revealed a preference of all dyes for GC DNA over AT DNA. Unlike TO and TOTO, the additional cyanine unit did not yield higher affinities of dimers compared to the monomer that excluded bisintercalation as the dominant binding mode, probably because of the steric influence of the hydroxypropyl group. All compounds revealed a bathochromic shift of electronic absorption bands and strong bisignate ICD signals upon titrations with DNA ds-polynucleotides that indicated uniform J-aggregation of dye chromophores along the polynucleotide chiral helix with well-defined supramolecular chirality. **4b** showed the strongest ICD signals among all ligands, possibly because its flexible polypropylene-ammonium linker enabled good accommodation of the dye and unique arrangement. The bispyridine linked dimer **4c** showed unique behavior upon thermal stabilization of prA–prU: it stabilized RNA at r values below 0.2 and destabilized it at higher ratios. This indicated possible bisintercalation at a low concentration of dye/RNA ratios. Further, **4c** strongly decreased the CD spectra of DNA polynucleotides and even changed the direction of its CD spectra. This significant perturbation of polynucleotide helicity was probably additionally influenced by a rigid aromatic di-pyridine linker. **4d** and **4e** with the piperidine linker differed the absence and presence of chloro-substitution on quinoline units, respectively. They both showed similar stabilizations of a double helix and similar stability constants, although **4d** revealed somewhat higher affinities for AT DNA compared to **4e.** However, differences in intensity and direction of their ICD signals stressed the importance of chloro-substitution, although detailed explanations were out of the scope of this paper.

In vitro studies of antiproliferative activities toward three cancer cell lines (colorectal carcinoma, breast carcinoma and large cell lung cancer) revealed that novel compounds inhibited cell growth in micromolar concentrations. Additionally, confocal microscopy demonstrated that the novel dye (**4c**) had selective antiproliferative affinity for tumor cells compared to healthy cells and efficiently entered cells, emitting a strong green fluorescence, when accumulated in cytoplasm organelles. Thus, novel dyes combine highly selective fluorescent intracellular probing with strong antiproliferative activity. This result makes them promising theragnostic candidates for further studies of the mechanism of their biological activity.

## Figures and Tables

**Table 1:** The ^a^ ΔT_m_ values (°C) of the studied ds-polynucleotides upon addition of different ratios ^b^ ***r*** of **4a**–**4e** at pH = 7.0 (buffer sodium cacodylate, *I* = 0.05 mol dm^−3^); ^c^ (polynucleotide) = 1–2 × 10^−5^ mol dm^−3^.

		Δ*Tm*/°C		
Compound	*r*	*ct*-DNA	Poly dAdT–Poly dAdT	Poly A–Poly U
**4a**	0.1 0.2 0.3	>20 ^c^	5.5 28.6 31.6	19.2 24.0 26.0
**4b**	0.1 0.2 0.3	2.2 5.3 8.6	2.7 8.9 12.4	1.3 3.0 3.4
**4c**	0.05 0.1 0.2 0.3	3.95 12.3 >15 ^c^ >15 ^c^	2.1 >20 ^c^ >20 ^c^ ^d^	9.1 10.6 −19.1 −16.8
**4d**	0.05 0.1 0.2 0.3	1.0 2.3 4.5 6.4	1.9 5.7 10.7 11.9	0.5 0 0 0
**4e**	0.05 0.1 0.2 0.3	0.7 2.7 5.2^d^	1.5 4.2 7.1 10.0	0 0.5 0.6 0

^a^ Error in Δ*T_m_*: ± 0.5 °C, ^b^ *r **=*** [compound]/[polynucleotide], ^c^ estimated value of ΔT_m_ since the melting point of polynucleotide was higher than 95 °C after addition of the compound, ^d^ not determined.

**Table 2:** Apparent stability constants (log*K_s_*) ^a^ for fixed ratios *n* [bound dye]/[polynucleotide] = 0.2 ^a^ and spectroscopic properties of complexes ^b^ of **4a**–**4e** with ds-polynucleotides calculated according to fluorimetric titrations (Na-cacodylate buffer, c = 0.05 mol dm^−3^, pH = 7.0, λ_exc_ = 510 nm, λ_em_ = 525–650 nm and c(**4a**–**4e**) = 1.3–1.5 × 10^−7^ mol dm^−3^).

		log *Ks*	Δ I_calc_ ^b^
**4a** λ_exc_ 510 nm	ct–DNA	6.57	377
	poly dA–poly dT	6.41	712
	poly dA-dT–poly dA-dT	6.65	358
	poly rA–poly rU	6.11	1573
	poly dG–poly dC	6.79	561
	poly dG-dC–poly dG-dC	6.98	895
	poly rG–poly rC	5.23	799
**4b** λ_exc_ 478 nm	ct–DNA	6.93	157
	poly dA–poly dT	6.19	44
	poly dA-dT–poly dA-dT	7.23	75
	poly rA–poly rU	7.15	158
	poly dG–poly dC	7.55	114
	poly dG-dC–poly dG-dC	7.64	65
	poly rG–poly rC	4.94	618
4c λ_exc_ 488 nm	ct–DNA	6.62	386
	poly dA–poly dT	6.23	151
	poly dA-dT–poly dA-dT	6.68	100
	poly rA–poly rU	6.22	255
	poly dG–poly dC	6.78	298
	poly dG-dC–poly dG-dC	7.04	485
	poly rG–poly rC	4.76	63
**4d** λ_exc_ 478 nm	ct–DNA	6.87	340
	poly dA–poly dT	5.89	143
	poly dA-dT–poly dA-dT	6.98	117
	poly rA–poly rU	6.14	78
	poly dG–poly dC	6.63	120
	poly dG-dC–poly dG-dC	7.20	285
	poly rG–poly rC	4.79	572
**4e** λ_exc_ 452 nm	ct–DNA	5.94	216
	poly dA–poly dT	5.91	329
	poly dA-dT–poly dA-dT	6.05	439
	poly rA–poly rU	6.14	35
	poly dG–poly dC	7.41	155
	poly dG-dC–poly dG-dC	7.40	285
	poly rG–poly rC	4.95	218

^a^ Titration data were processed using the Scatchard equation [22,23], calculations mostly gave values of ratio *n* [bound dye]/[polynucleotide] = 0.1 – 0.3; for comparison, all log *Ks* values were recalculated with fixed value *n* = 0.2; correlation coefficients were > 0.98–0.99 for all calculated *Ks*, ^b^ emission change; ΔI_calc_ = I_lim_ − I_0_; emission intensity change, calculated from titration data processed by the Scatchard equation, where I_0_ is the calculated intensity of the examined compound, while I_lim_ is the calculated intensity of the complex.

**Table 3:** Antiproliferative activity of **4a–4e** compounds against solid tumor cell lines expressed as the **GI_50_^a^** value.

GI_50_^a^ (μM)			
Compound	Cell Lines		
	HCT 116	MCF-7	H460
**4a**	6 ± 3	5 ± 4	10 ± 7
**4b**	4.5 ± 0.7	2 ± 0.6	7 ± 2
**4c**	2.2 ± 0.6	3.2 ± 1.9	1.9 ± 1.0
**4d**	2.9 ± 0.1	3.5 ± 1.2	6.1 ± 4.4
**4e**	3.3 ± 0.2	9.7 ± 2.9	4.6 ± 3.1

^a^ GI_50_; drug concentration that causes 50% cell growth inhibition; data represent the mean ± SD of three independent experiments. Cells in the exponential growth phase were treated with substances for 72 h and cytotoxicity was analyzed by the MTT cell survival assay.

## Supplementary Materials

The following are available online at https://www.mdpi.com/article/10.3390/biom11081075/s1, Scheme S1: Structures of examined **4a**–**4e** compounds, Figure S1: UV/Vis spectra of **4a**–**4e** at pH = 7, sodium cacodylate/HCl buffer, I = 0.05 mol dm^−3^, Table S1: Electronic absorption data of **4a**–**4e** (sodium cacodylate buffer, *I* = 0.05 mol dm^−3^, pH = 7.), Table S2: Groove widths and depths for selected nucleic acid conformations, Figure S2: Normalized melting curves of ct DNA (left) and poly dAdT–poly dAdT (right) upon addition of **4a** (ratios *r* = [compound]/[polynucleotide] indicated in the graph legend, Figure S3: Normalized melting curves of ct DNA (left) and poly dAdT–poly dAdT (right) upon addition of **4b** (ratios r=[compound]/[polynucleotide] indicated in the graph legend, Figure S4: Normalized melting curves of ct DNA (left) and poly dAdT–poly dAdT (right) upon addition of **4c** (ratios *r* = [compound]/[polynucleotide] indicated in the graph legend, Figure S5: Normalized melting curves of ct DNA (left) and poly dAdT–poly dAdT (right) upon addition of **4d** (ratios r = [compound]/[polynucleotide] indicated in the graph legend, Figure S6: Normalized melting curves of ct DNA (left) and poly dAdT–poly dAdT (right) upon addition of **4e** (ratios r = [compound]/[polynucleotide] indicated in the graph legend, Figure S7: Normalized melting curves of poly rA–poly rU upon addition of **4a** (left) and **4b** (right) (ratios r = [compound]/[polynucleotide] indicated in the graph legend, Figure S8: Normalized melting curves of poly rA–poly rU upon addition of **4c** (left) and **4d** (right) (ratios r = [compound]/[polynucleotide] indicated in the graph legend, Figure S9: Left: Normalized melting curves of poly rA–poly rU upon addition of **4e** (ratios r=[compound]/[polynucleotide] indicated in the graph legend. Right: Correlation of ΔT_m_ values and ratios *r*_[compound]/[polynucleotide]_ for complexes of examined compounds with different polynucleotides, Figure S10: Changes in UV–Vis spectra upon addition of *ct*-DNA: (a1) UV/Vis titration of **4a** (*c* = 1.11 × 10^−5^ mol dm^−3^); (a2) changes in UV/Vis spectra of **4a** at λ = 480 nm; (b1) UV/Vis titration of **4b** (*c* = 8.03 × 10^−6^ mol dm^−3^); (b2) changes in UV/Vis spectra of **4b** at λ_max_ = 519 nm; (c1) UV/Vis titration of **4c** (*c*= 7.41 × 10^−6^ mol dm^−3^); (c2) changes in UV/Vis spectra of **4c** at λ_max_ = 484 nm; d1) UV/Vis titration of **4d** (*c* = 8.03 × 10^-6^ mol dm^−3^); (d2) changes in UV/Vis spectra of **4d** at λ_max_ = 478 nm; (e1) UV/Vis titration of **4e** (*c* = 9 × 10^-6^ mol dm^−3^); (e2) changes in UV/Vis spectra of **4e** at λ_max_ = 452 nm, Table S3: Apparent stability constants (log*K_s_*) ^a^, ratios *n* ^a^ *=* [bound compounds]/[polynucleotide] and spectroscopic properties of complexes of **4a**–**4e** with *ct-*DNA calculated according to UV–Vis titrations (Na-cacodylate buffer, c = 0.05 mol dm^−3^, pH = 7.0, λ =400-600 nm, c (**4a**) ≈ 1 × 10^−5^ mol dm^−3^), Figure S11: Fluorimetric titration of **4a**–**4e** compounds (c = 1.3–1.5 × 10^−7^ mol dm^−3^) with ***ct*-DNA**: a1) experimental (●) and calculated (–) fluorescence intensities of **4a** at λ_em_ = 570 nm **4a** (λ_exc_ = 510 nm); (b1) fluorimetric titration of **4b** (λ_exc_ = 478 nm); (b2) experimental (●) and calculated (–) fluorescence intensities of **4b** at λ = 548 nm; (c1) **4c** (λ_exc_ = 484 nm); (c2) experimental (●) and calculated (–) fluorescence intensities of **4c** at λ = 538 nm; d1) **4d** (λ_exc_ = 484 nm); (d2) experimental (●) and calculated (–) fluorescence intensities of **4d** at λ = 550 nm; e1) **4e** (λ_exc_ = 452 nm); (e2) experimental (●) and calculated (–) fluorescence intensities of **4e** at λ = 538 nm, Figure S12: Fluorimetric titration of **4a**–**4e** compounds (c = 1.3–1.5 × 10^−7^ mol dm^−3^) with **poly dA**–**poly dT**: (a1) **4a** (λ_exc_=510 nm); (a2) experimental (●) and calculated (–) fluorescence intensities of **4a** at λ_em_ = 547 nm; (b1) fluorimetric titration of **4b** (λ_exc_ = 478 nm); (b2) experimental (●) and calculated (–) fluorescence intensities of **4b** at λ = 545 nm; (c1) **4c** (λ_exc_ = 484 nm); (c2) experimental (●) and calculated (–) fluorescence intensities of **4c** at λ = 538 nm; (d1) **4d** (λ_exc_ = 484 nm); (d2) experimental (●) and calculated (–) fluorescence intensities of **4d** at λ = 554 nm; (e1) **4e** (λ_exc_ = 452 nm); (e2) experimental (●) and calculated (–) fluorescence intensities of **4e** at λ = 554 nm, Figure S13: Fluorimetric titration of **4a**–**4e** compounds (c = 1.3-1.5 × 10^−7^ mol dm^−3^) with **poly dAdT–poly dAdT**: (a1) **4a** (λ_exc_ = 510 nm); (a2) experimental (●) and calculated (–) fluorescence intensities of **4a** at λ_em_ = 535 nm; (b1) fluorimetric titration of **4b** (λ_exc_ = 478 nm); (b2) experimental (●) and calculated (–) fluorescence intensities of **4b** at λ = 545 nm; (c1) **4c** (λ_exc_=484 nm); (c2) experimental (●) and calculated (–) fluorescence intensities of **4c** at λ = 538 nm; (d1) **4d** (λ_exc_ = 484 nm); (d2) experimental (●) and calculated (–) fluorescence intensities of **4d** at λ = 548 nm; e1) **4e** (λ_exc_ = 452 nm); (e2) experimental (●) and calculated (–) fluorescence intensities of **4e** at λ = 540 nm, Figure S14: Fluorimetric titration of **4a**–**4e** compounds (c = 1.3–1.5 × 10^−7^ mol dm^−3^) with **poly rA**–**poly rU**: (a1) **4a** (λ_exc_ = 510 nm); (a2) experimental (●) and calculated (–) fluorescence intensities of **4a** at λ_em_ = 543 nm; (b1) fluorimetric titration of **4b** (λ_exc_ = 478 nm); (b2) experimental (●) and calculated (–) fluorescence intensities of **4b** at λ = 550 nm; (c1) **4c** (λ_exc_ = 484 nm); (c2) experimental (●) and calculated (–) fluorescence intensities of **4c** at λ = 538 nm; (d1) **4d** (λ_exc_ = 484 nm); (d2) experimental (●) and calculated (–) fluorescence intensities of **4d** at λ = 548 nm; (e1) **4e** (λ_exc_ = 452 nm); (e2) experimental (●) and calculated (–) fluorescence intensities of **4e** at λ = 557 nm, Figure S15: Fluorimetric titration of **4a**–**4e** compounds (c = 1.3–1.5 × 10^-7^ mol dm^-3^) with **poly dG**–**poly dC**: (a1) **4a** (λ_exc_ = 510 nm); (a2) experimental (●) and calculated (–) fluorescence intensities of **4a** at λ_em_ = 538 nm; b1) fluorimetric titration of **4b** (λ_exc_ = 478 nm); (b2) experimental (●) and calculated (–) fluorescence intensities of **4b** at λ = 550 nm; (c1) **4c** (λ_exc_ = 484 nm); (c2) experimental (●) and calculated (–) fluorescence intensities of **4c** at λ = 538 nm; (d1) **4d** (λ_exc_ = 484 nm); (d2) experimental (●) and calculated (–) fluorescence intensities of **4d** at λ = 550 nm; (e1) **4e** (λ_exc_=452 nm); (e2) experimental (●) and calculated (–) fluorescence intensities of **4e** at λ = 575 nm, Figure S16: Fluorimetric titration of **4a**–**4e** compounds (c = 1.3–1.5 × 10^−7^ mol dm^−3^) with **polydGdC**–**poly dGdC**: (a1) **4a** (λ_exc_ = 510 nm); (a2) experimental (●) and calculated (–) fluorescence intensities of **4a** at λ_em_ = 535 nm; (b1) fluorimetric titration of **4b** (λ_exc_ = 478 nm); (b2) experimental (●) and calculated (–) fluorescence intensities of **4b** at λ = 550 nm; (c1) **4c** (λ_exc_ = 484 nm); (c2) experimental (●) and calculated (–) fluorescence intensities of **4c** at λ = 538 nm; (d1) **4d** (λ_exc_ = 484 nm); (d2) experimental (●) and calculated (–) fluorescence intensities of **4d** at λ = 548 nm; (e1) **4e** (λ_exc_ = 452 nm); (e2) experimental (●) and calculated (–) fluorescence intensities of **4e** at λ = 555 nm, Figure S17: Fluorimetric titration of **4a**–**4e** compounds (c = 1.3-1.5 × 10^−7^ mol dm^−3^) with **poly rG**–**poly rC**: (a1) **4a** (λ_exc_ = 510 nm); (a2) experimental (●) and calculated (–) fluorescence intensities of **4a** at λ_em_ = 538 nm; (b1) fluorimetric titration of **4b** (λ_exc_ = 478 nm); (b2) experimental (●) and calculated (–) fluorescence intensities of **4b** at λ = 550 nm; c1) **4c** (λ_exc_=484 nm); (c2) experimental (●) and calculated (–) fluorescence intensities of **4c** at λ = 538 nm; (d1) **4d** (λ_exc_ = 484 nm); (d2) experimental (●) and calculated (–) fluorescence intensities of **4d** at λ = 548 nm; e1) **4e** (λ_exc_ = 452 nm); (e2) experimental (●) and calculated (–) fluorescence intensities of **4e** at λ = 555 nm, Figure S18: Changes of fluorescence emission at emission maxima of (a) **4a** (c = 1.3 × 10^−7^ mol dm^-3^); (b) **4b** (c = 1.3 × 10^−7^ mol dm^−3^); (c) **4c** (c = 1.48 × 10^−7^ mol dm^−3^); (d) **4d** (c = 1.34 × 10^−7^ mol dm^−3^); (e) **4e** (c = 1.34 × 10^−7^ mol dm^−3^) upon addition of *ds*-polynucleotides of at pH 7 (Na-cacodylate buffer, pH = 7.0, *I* = 0.05 M), Figure S19: CD titration of polynucleotides (*c* = 2.0 × 10^−5^ mol dm^−3^) with **4a** at different molar ratios ***r*** = [compound]/[polynucleotide] (pH = 7.0, buffer sodium cacodylate, *I* = 0.05 mol dm^−3^), Figure S20: CD titration of polynucleotides (*c* = 1.0 × 10^−5^ mol dm^−3^) with **4b** at different molar ratios ***r*** = [compound]/[polynucleotide] (pH = 7.0, buffer sodium cacodylate, *I* = 0.05 mol dm^-3^), Figure S21: CD titration of polynucleotides (*c* = 1.0 × 10^−5^ mol dm^−3^) with **4c** at different molar ratios ***r*** = [compound]/[polynucleotide] (pH = 7.0, buffer sodium cacodylate, *I* = 0.05 mol dm^-3^), Figure S22: CD titration of polynucleotides (*c* = 1.0 × 10^−5^ mol dm^−3^) with **4d** at different molar ratios ***r*** = [compound]/[polynucleotide] (pH = 7.0, buffer sodium cacodylate, *I* = 0.05 mol dm^−3^), Figure S23: CD titration of polynucleotides (*c* = 1.0 × 10^−5^ mol dm^−3^) with **4e** at different molar ratios ***r*** = [compound]/[polynucleotide] (pH = 7.0, buffer sodium cacodylate, *I* = 0.05 mol dm^−3^), Figure S24: Dose–response profiles for compound **4a**–**4e** compounds tested in vitro.
