# Supplementary material for: Polycationic Monomeric and Homodimeric Asymmetric Monomethine Cyanine Dyes with Hydroxypropyl Functionality—Strong Affinity Nucleic Acids Binders
Source: Biomolecules. 2021 Jul 21;11(8):1075. doi: 10.3390/biom11081075 (PMC8391988; doi:10.3390/biom11081075)
Supplement: Supplementary file 1 [file biomolecules-11-01075-s001.zip › biomolecules-1279521-supplementary.pdf]

Polycationic Monomeric and Homodimeric Asymmetric Monomethine Cyanine dyes with Hydroxypropyl Functionality – Strong Affinity Nucleic Acids Binders

Ivana Mikulin[a#], Ivana Sorić[a#], Ivo Piantanida[a], Aleksey Vasilev[b]*, Mihail Mondeshki[d], Meglena Kandinska[b], Lidija Uzelac[c], Irena Martin-Kleiner[c], Marijeta Kralj[c], Lidija-Marija Tumir[a]*

[a]Laboratory for Biomolecular Interactions and Spectroscopy, Division of Organic Chemistry and Biochemistry, Ruđer Bošković Institute, HR 10002 Zagreb, P.O.B. 180, Croatia, E-mail: tumir@irb.hr

[b]Department of Pharmaceutical and Applied Organic Chemistry, Faculty of Chemistry and Pharmacy, Sofia University "St. Kliment Ohridski", Sofia, Bulgaria, E-mail: ohtavv@chem.uni-sofia.bg

[c] Laboratory of Experimental Therapy, Division of Molecular Medicine, Ruđer Bošković Institute, HR 10002 Zagreb, P.O.B. 180, Croatia

[d] Department of Chemistry, Johannes Gutenberg Universität Mainz, Duesbergweg 10-14, 55128 Mainz, Germany

#equal contribution

Supporting Information

1. Spectroscopic properties of **4a-4e** compounds
2. Interactions of **4a-4e** compounds with DNA/RNA
3. Biology

**4a**
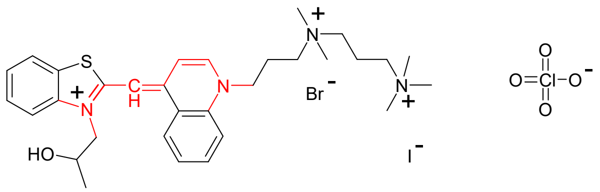
**4b**
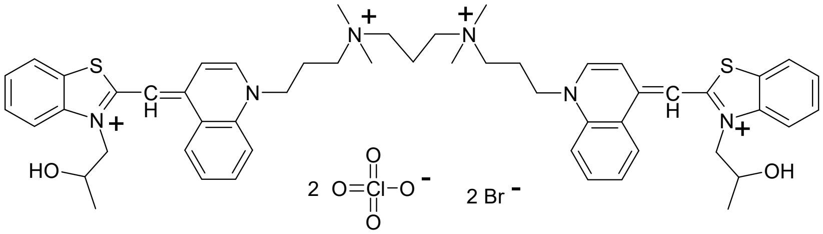
**4c**

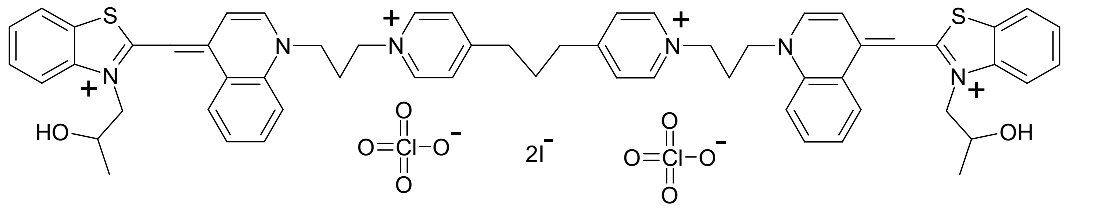

4d

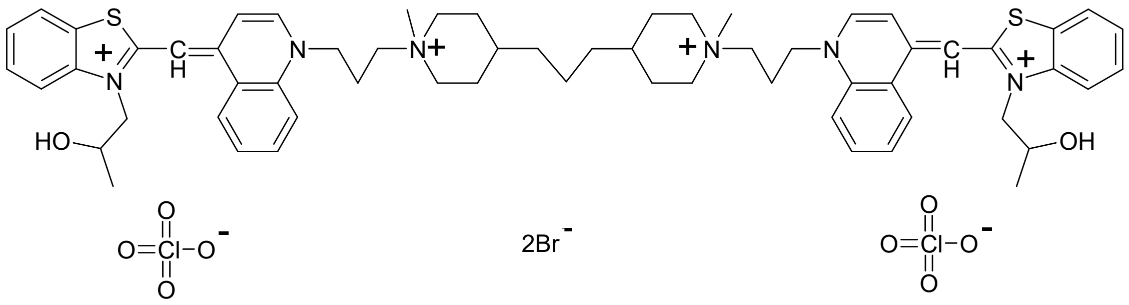

4e

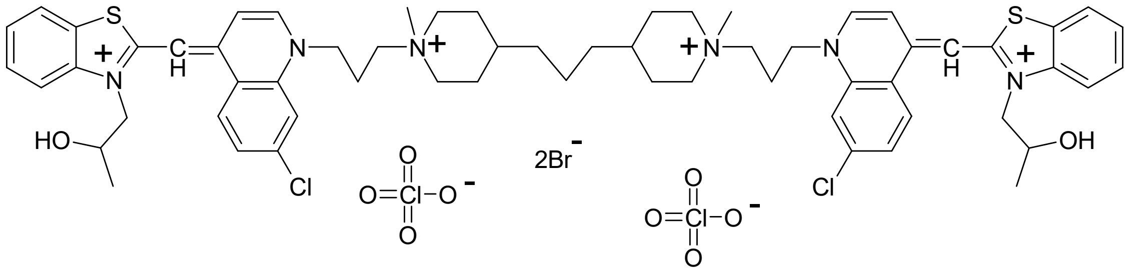

Scheme S1. Structures of examined **4a-4e** compounds

1. Spectroscopic properties of 4a-4e compounds

Absorption maxima and corresponding molar extinction coefficients (ε) are given in Table S1 and Figure S1.

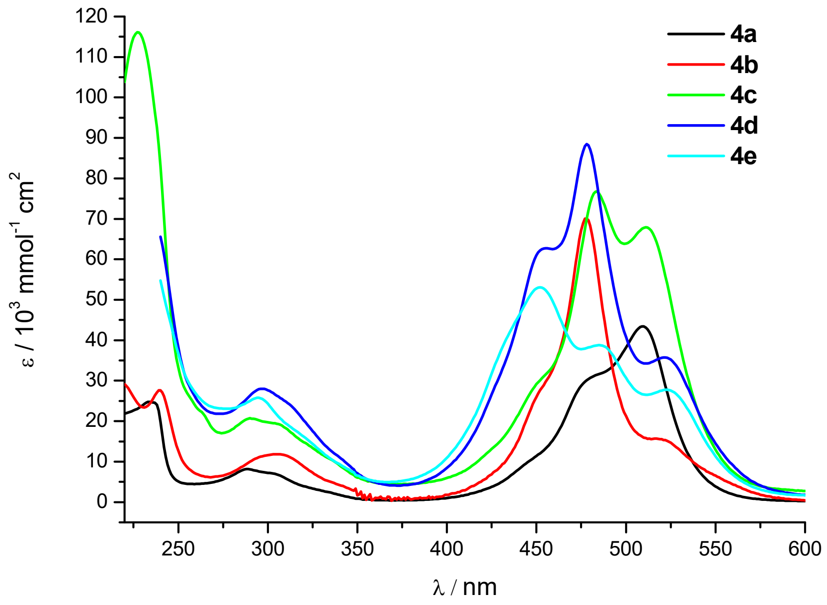

Figure S1. UV/Vis spectra of **4a-4e** at pH=7, sodium cacodylate/HCl buffer, I = 0.05 mol dm^{-3}.

Table S1. Electronic absorption data of **4a-4e** (Sodium cacodylate buffer, *I* = 0.05 mol dm^{-3}, pH = 7.)

	λ_{max} / nm	ε / mmol^{-1} cm^{2}
**4a**	235	25062
	478	31079
	509	44971
**4b**	239	34818
	478	70024
	509	15982
**4c**	227	115617
	484	76515
	511	67971
**4d**	296	28848
	455	63848
	478	84394
	521	36220
**4e**	294	25736
	452	53588
	486	39230
	523	27929

2. Study of interactions of 4a - 4e with ds-DNA and ds-RNA in aqueous media

Table S2. Groove widths and depths for selected nucleic acid conformations [1]

	Groove width [Å]		Groove depth [Å]	
	minor	major	minor	major

[a] poly dAdT – poly dAdT	6.3	11.2	7.5	8.5
[a] poly dA – poly dT	3.3	11.4	7.9	7.5
[b] poly rA- poly rU	10.9	3.8	2.8	13.5
[a] poly dGdC – poly dGdC	9.5	13.5	7.2	10.0

[a] B-helical structure (e.g. B-DNA); [b] A-helical structure (e.g. A-DNA or A-RNA).

2.1. Thermal melting experiments

All thermal melting experiments were performed sodium cacodylate buffer, pH = 7.0, I = 0.05 mol dm^{-3}; c (polynucleotide) = 1-2 $\times$ 10^{-5} mol dm^{-3}

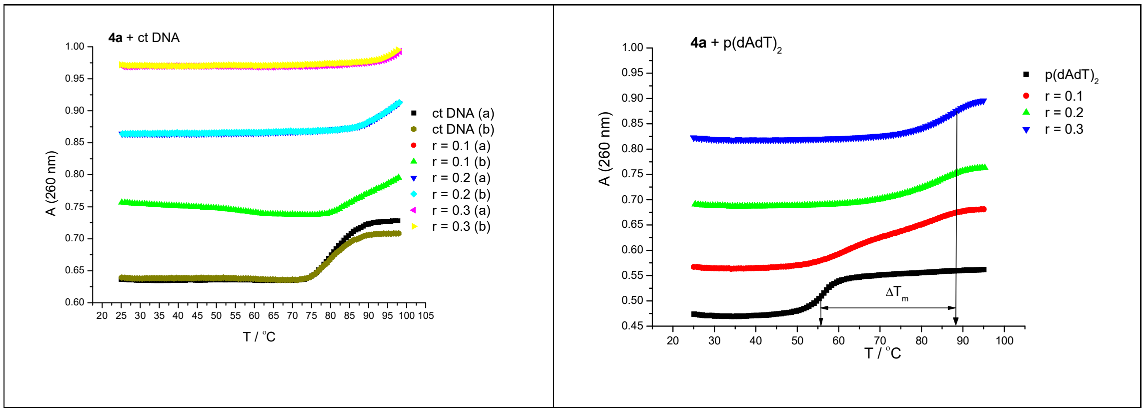

Figure S2**.** Normalized melting curves of ct DNA (left) and poly dAdT – poly dAdT (right) upon addition of **4a** (ratios *r*=[compound] / [polynucleotide] indicated in the graph legend.

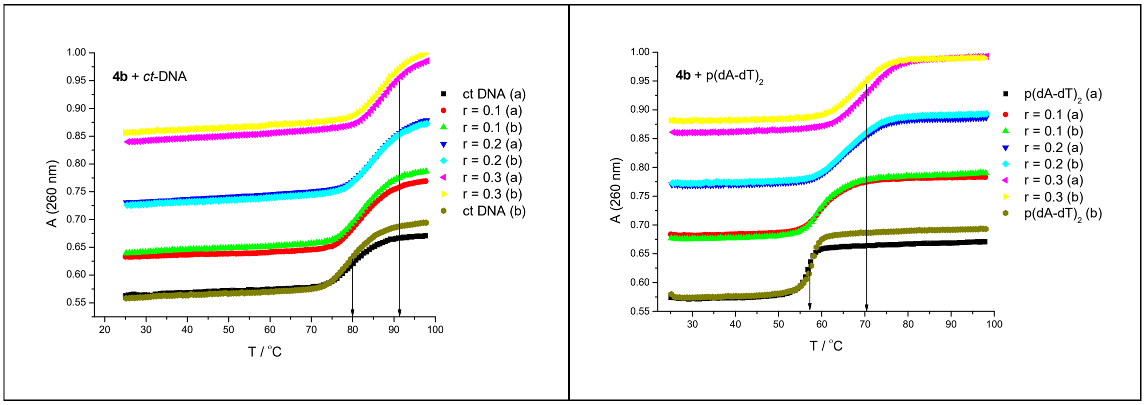

Figure S3. Normalized melting curves of ct DNA (left) and poly dAdT – poly dAdT (right) upon addition of **4b** (ratios r=[compound] / [polynucleotide] indicated in the graph legend.

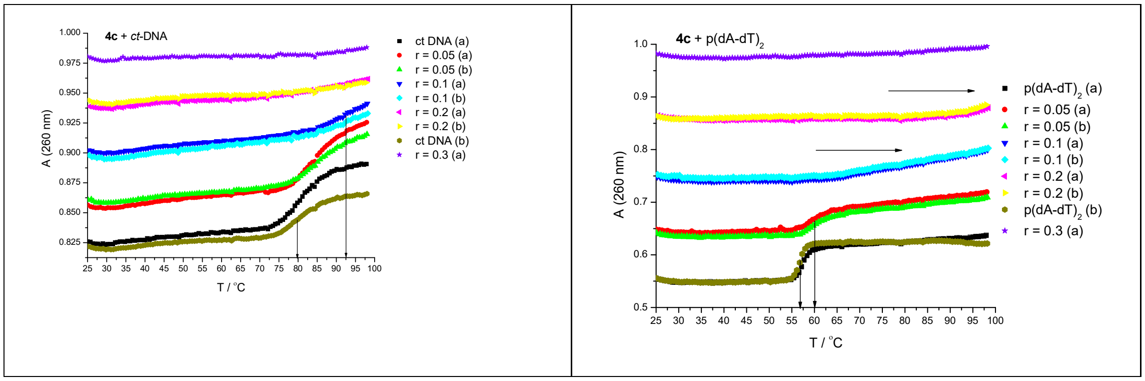

Figure S4. Normalized melting curves of ct DNA (left) and poly dAdT – poly dAdT (right) upon addition of **4c** (ratios *r*=[compound] / [polynucleotide] indicated in the graph legend.

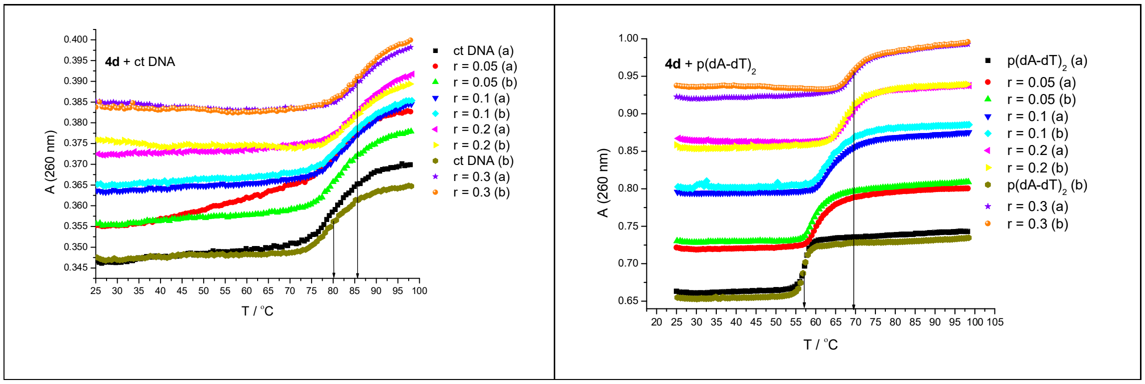

Figure S5. Normalized melting curves of ct DNA (left) and poly dAdT – poly dAdT (right) upon addition of **4d** (ratios r=[compound] / [polynucleotide] indicated in the graph legend.

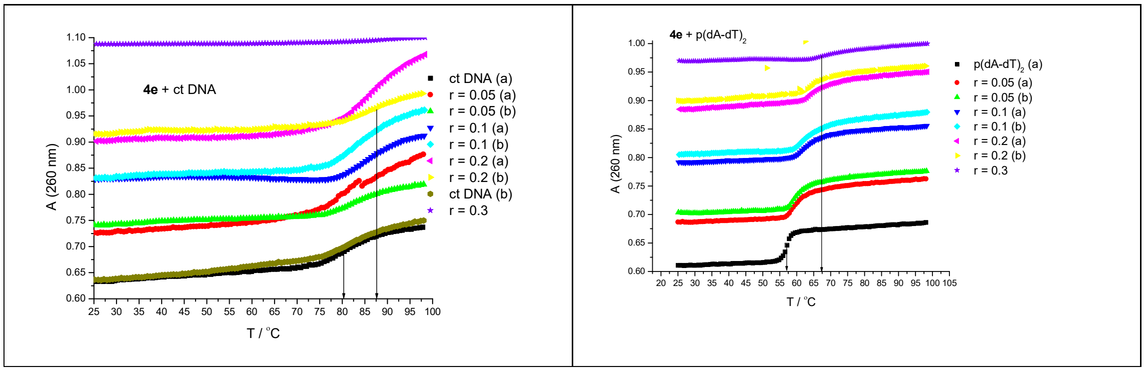

Figure S6. Normalized melting curves of ct DNA (left) and poly dAdT – poly dAdT (right) upon addition of **4e** (ratios r=[compound] / [polynucleotide] indicated in the graph legend.

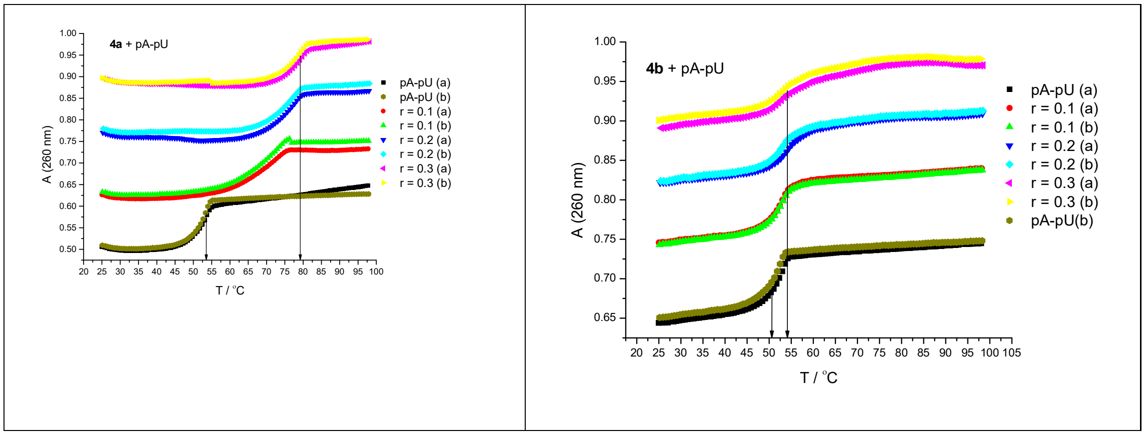

Figure S7**.** Normalized melting curves of poly rA – poly rU upon addition of **4a** (left) and **4b** (right) (ratios r=[compound] / [polynucleotide] indicated in the graph legend.

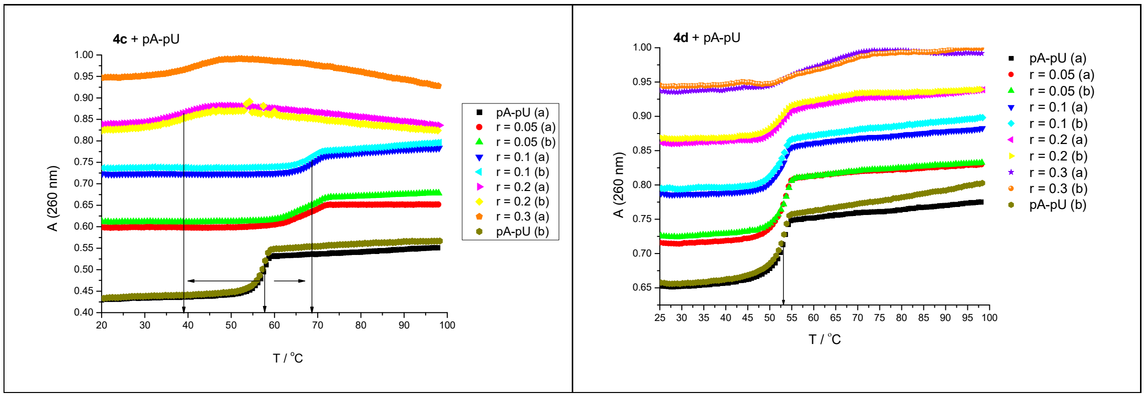

Figure S8**.** Normalized melting curves of poly rA – poly rU upon addition of **4c** (left) and **4d** (right) (ratios r=[compound] / [polynucleotide] indicated in the graph legend.

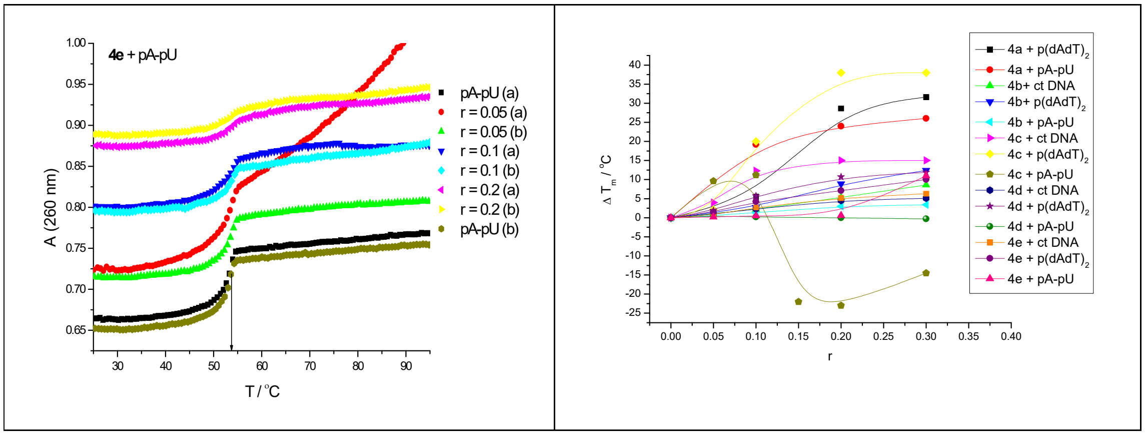

Figure S9. Left: Normalized melting curves of poly rA – poly rU upon addition of **4e** (ratios r=[compound] / [polynucleotide] indicated in the graph legend. Right: Correlation of ΔT_m values and ratios $r_{[compound] / [polynucleotide]}$ for complexes of examined compounds with different polynucleotides.

2.2. Spectrophotometric titrations

Addition of *ct*-DNA resulted in strong bathochromic and hypochromic effects of UV/Vis spectra of studied compounds (Figure S10, spectral changes observed in UV/Vis titrations for all compounds are summarized in Table S3). All UV-Vis titrations were performed at pH=7, sodium cacodylate buffer, I = 0.05 mol dm^{-3}.

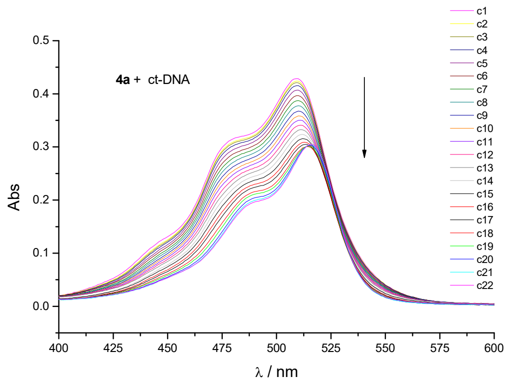

a1)

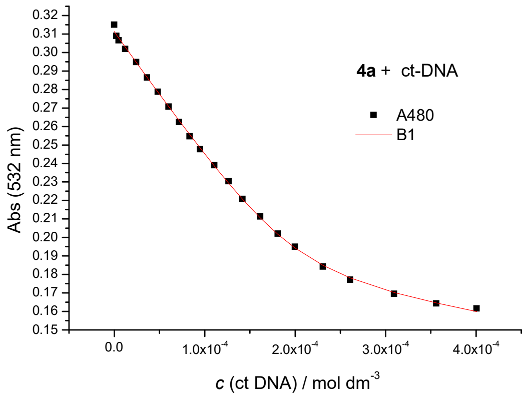

a2)

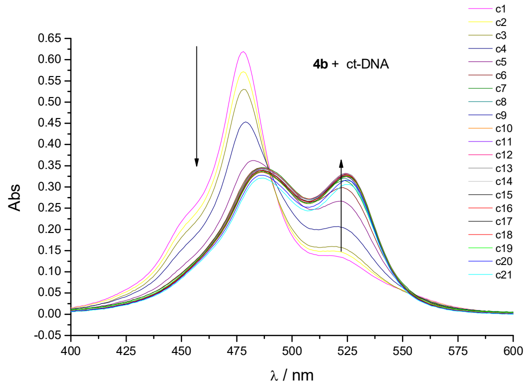

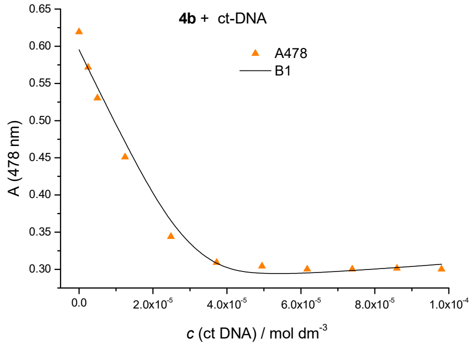

b2)

b1)

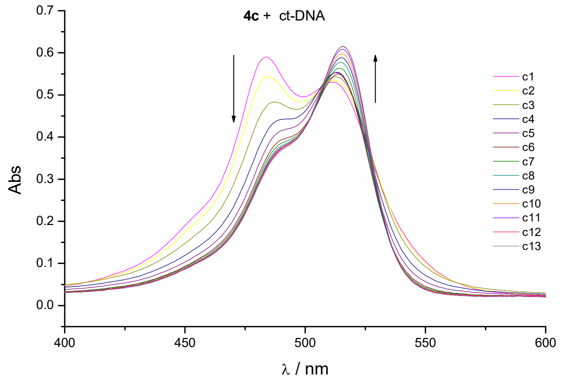

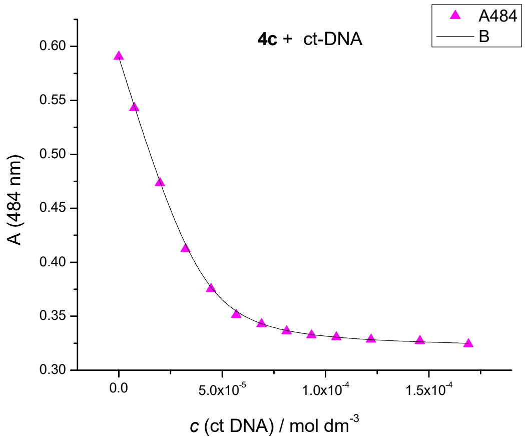

c1)

c2)

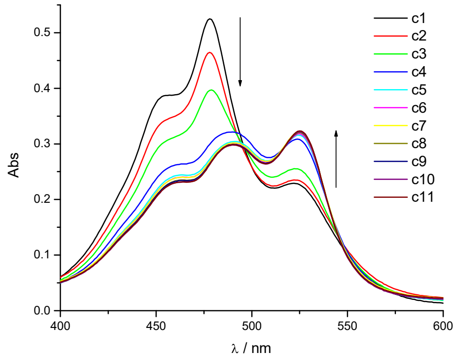

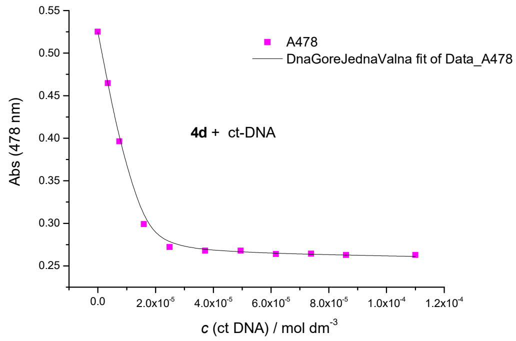

d1)

d2)

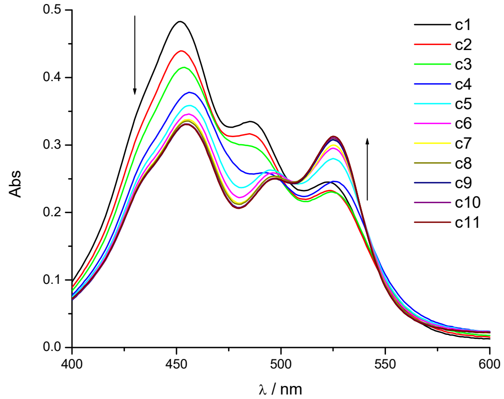

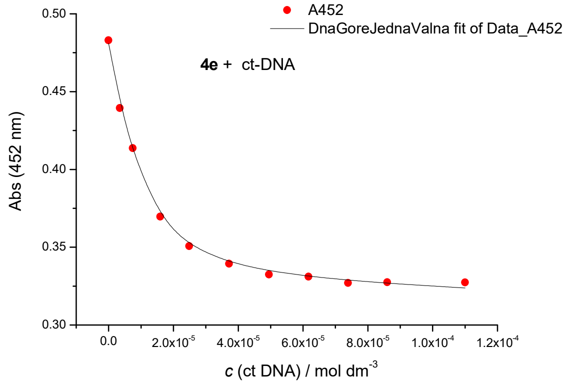

e1)

e2)

Figure S10. Changes in UV-Vis spectra upon addition of *ct*-DNA: a1) UV/Vis titration of **4a** (c= 1.11 × 10^{-5} mol dm^{-3}); a2) changes in UV/Vis spectra of **4a** at λ = 480 nm b1) UV/Vis titration of **4b** (c= 8.03 × 10^{-6} mol dm^{-3}); b2) Changes in UV/Vis spectra of **4b** at λ_{max} = 519 nm; c1) UV/Vis titration of **4c** (c= 7.41 × 10^{-6} mol dm^{-3}); c2) Changes in UV/Vis spectra of **4c** at λ_{max} = 484 nm, d1) UV/Vis titration of **4d** (c= 8.03 × 10^{-6} mol dm^{-3}); d2) Changes in UV/Vis spectra of **4d** at λ_{max} = 478 nm; e1) UV/Vis titration of **4e** (c= 9 × 10^{-6} mol dm^{-3}); e2) Changes in UV/Vis spectra of **4e** at λ_{max} = 452 nm.

Table S3. Apparent stability constants (logK_s)[a], ratios n^a = [bound compounds] / [polynucleotide] and spectroscopic properties of complexes of **4a** - **4e** with *ct*-DNA calculated according to UV-Vis titrations (Na-cacodylate buffer, c = 0.05 mol dm^{-3}, pH = 7.0, λ =400-600 nm, c(**4a**) ≈ 1 × 10^{-5} mol dm^{-3}).

	log *Ks*	n	Δ A (%)[b]	Δλ / nm[c]
**4a**	6.28	0.05	-40%	+7
**4b**	5.99	0.26	-50%	+10
**4c**	6.37	0.18	-44%	+10
**4d**	6.65	0.42	-50%	+12
**4e**	6.02	0.28	-50%	+4

[a]Titration data were processed using Scatchard equation[2]; coefficients of correlation were >0.989-0.999 for all calculated *Ks;* [b]Changes of absorbance of compounds **4a** - **4e** induced by complex formation ($\Delta A = 100 \times (A_{lim} - A_0) / A_0$; A_0 is calculated absorbance of examined compound, while A_{lim} is calculated absorbance of complex); [c]Changes of wavelength of absorbance maxima $\Delta\lambda = \lambda_{max}$(complex) - λ_{max} (pure compound); referring to absorption maxima 478-485 nm.

All fluorimetric titrations (Figure S11-Figure S17) were performed at pH=7, sodium cacodylate buffer, I = 0.05 mol dm^{-3} at the wavelength range (λ_{exc}=480-510 nm, λ_{em}=525-650 nm) significantly distanced from the absorbance range of the DNA/RNA (λ=220-300 nm).Stability constants (manuscript, Table 2) and calculated intensities were obtained by means of Scatchard equation[2].

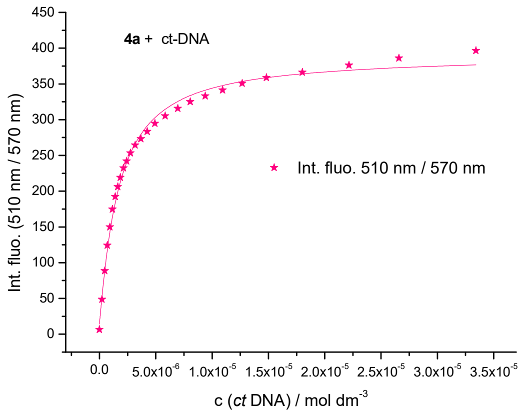

a1)

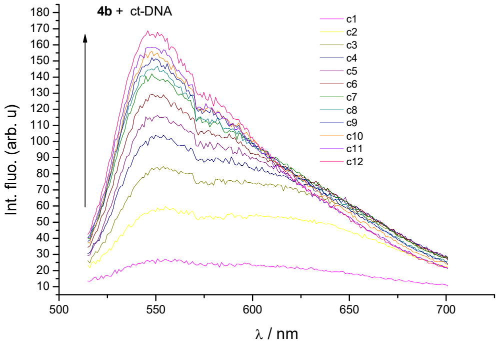

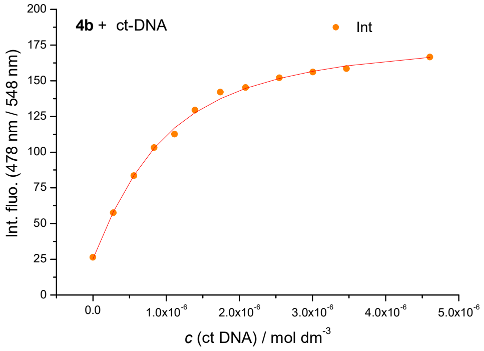

b1)

b2)

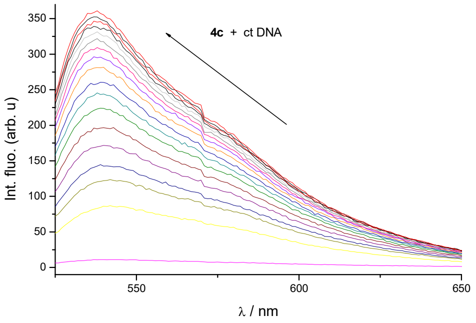

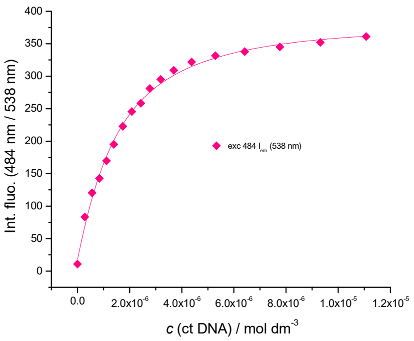

c1)

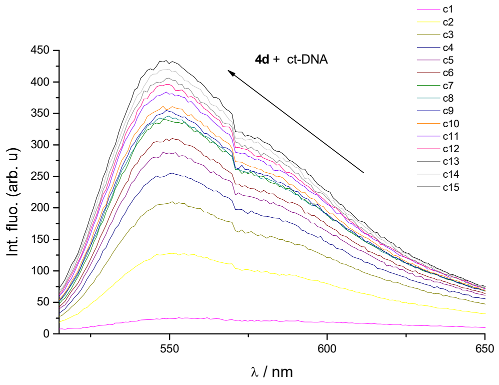

c2)

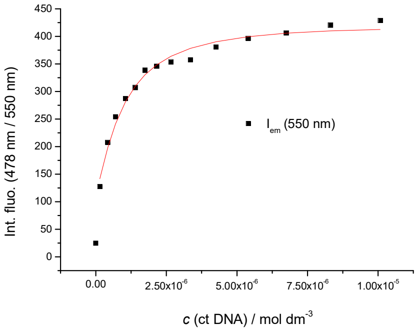

d2)

d1)

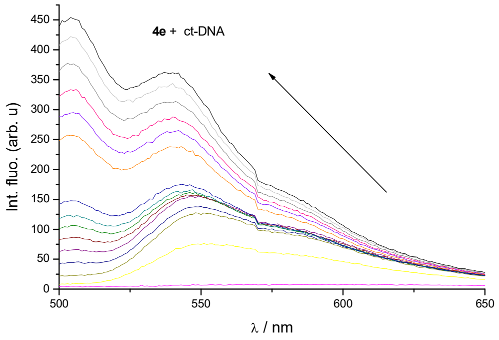

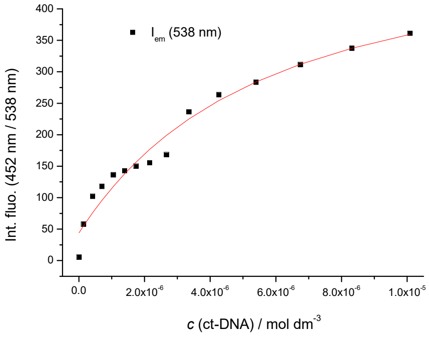

e1)

e1)

Figure S11. Fluorimetric titration of **4a-4e** compounds (c = 1.3-1.5 × 10^{-7} mol dm^{-3}) with ***ct*-DNA**: a1) Experimental (●) and calculated (–) fluorescence intensities of **4a** at λ_{em} = 570 nm **4a** (λ_{exc}=510 nm); b1) Fluorimetric titration of **4b** (λ_{exc}=478 nm); b2) Experimental (●) and calculated (–) fluorescence intensities of **4b** at λ = 548 nm; c1) **4c** (λ_{exc}=484 nm); c2) Experimental (●) and calculated (–) fluorescence intensities of **4c** at λ = 538 nm; d1) **4d** (λ_{exc}=484 nm); d2) Experimental (●) and calculated (–) fluorescence intensities of **4d** at λ = 550 nm; e1) **4e** (λ_{exc}=452 nm) e2) Experimental (●) and calculated (–) fluorescence intensities of **4e** at λ = 538 nm

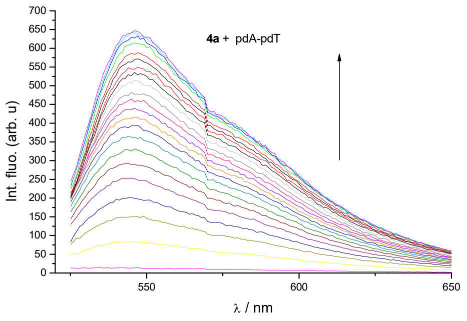

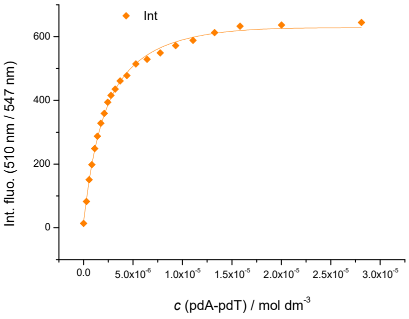

a1)

a2)

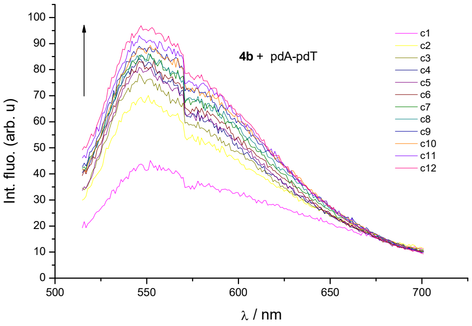

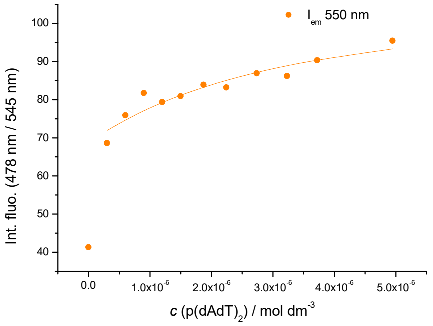

b1) b2)

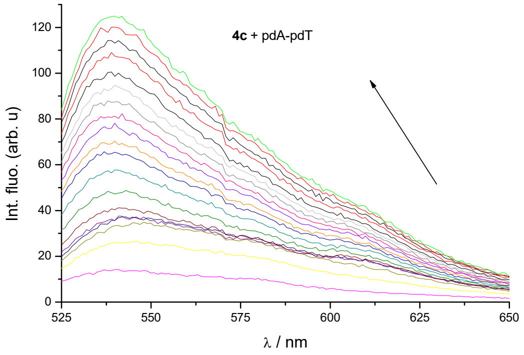

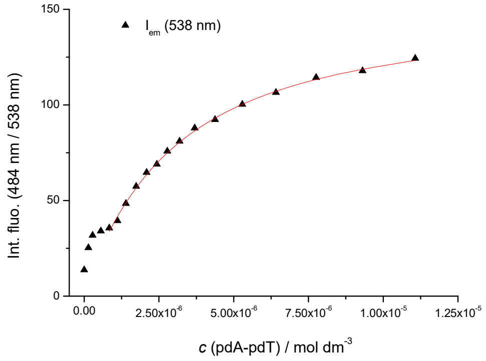

c1) c2)

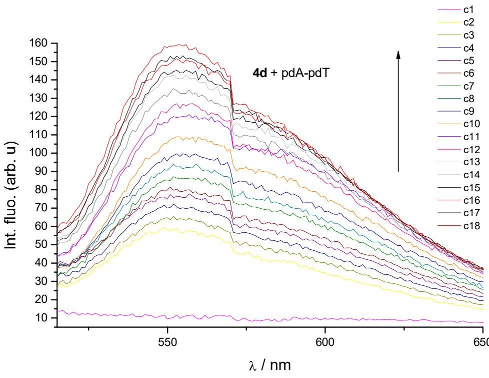

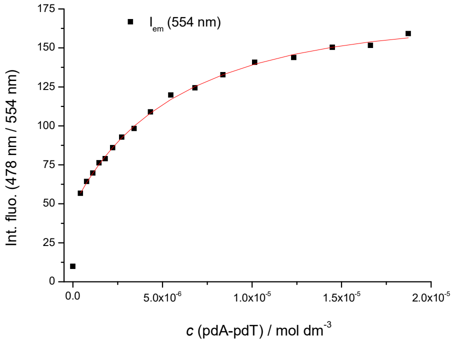

d1) d2)

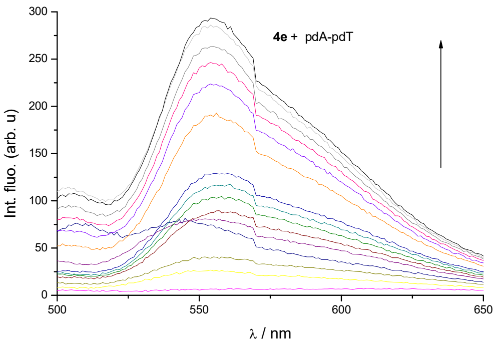

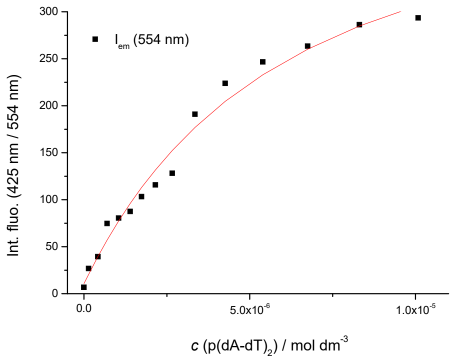

e1)

e1)

Figure S12. Fluorimetric titration of **4a-4e** compounds (c = 1.3-1.5 × 10^{-7} mol dm^{-3}) with **poly dA – poly dT**: a1) **4a** (λ_{exc}=510 nm); a2) Experimental (●) and calculated (–) fluorescence intensities of **4a** at λ_{em} = 547 nm: b1) Fluorimetric titration of **4b** (λ_{exc}=478 nm); b2) Experimental (●) and calculated (–) fluorescence intensities of **4b** at λ = 545 nm; c1) **4c** (λ_{exc}=484 nm); c2) Experimental (●) and calculated (–) fluorescence intensities of **4c** at λ = 538 nm; d1) **4d** (λ_{exc}=484 nm); d2) Experimental (●) and calculated (–) fluorescence intensities of **4d** at λ = 554 nm; e1) **4e** (λ_{exc}=452 nm) e2) Experimental (●) and calculated (–) fluorescence intensities of **4e** at λ = 554 nm

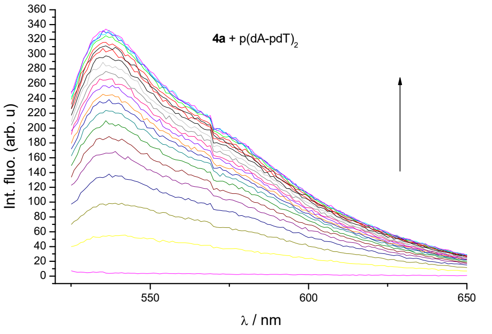

a1)

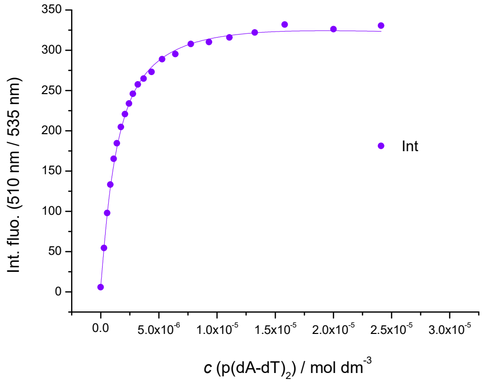

a2)

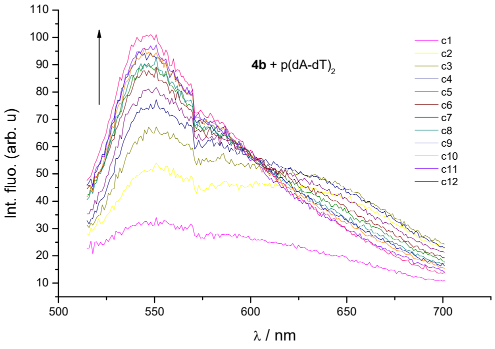

b1)

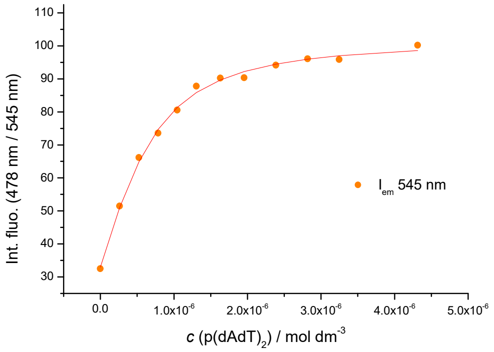

b2)

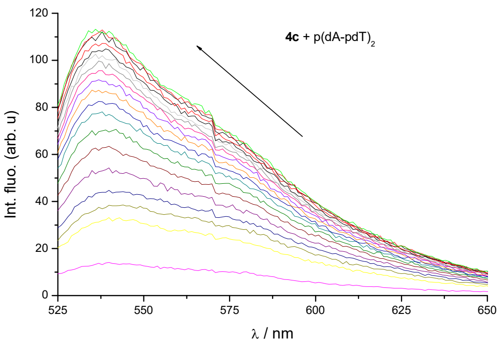

c1)

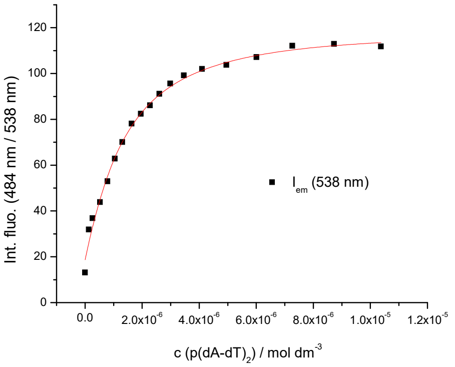

c2)

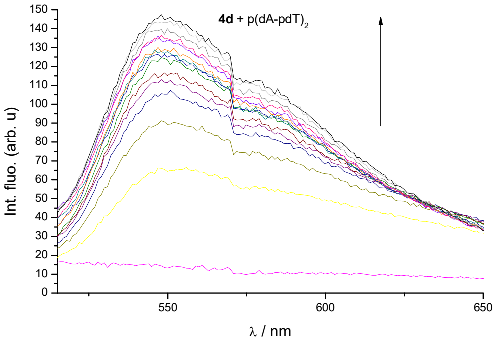

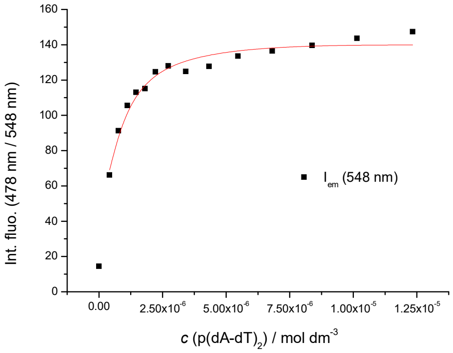

d1)

d2)

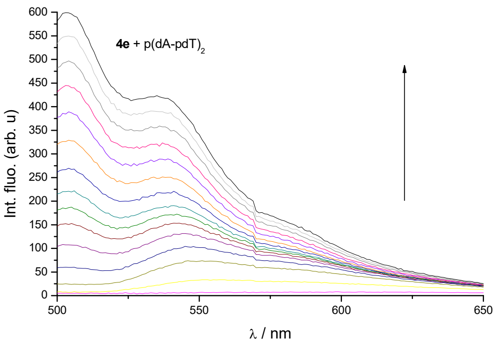

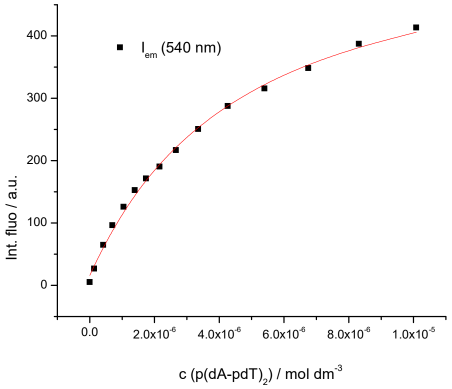

e1)

e1)

Figure S13. Fluorimetric titration of **4a-4e** compounds (c = 1.3-1.5 × 10^{-7} mol dm^{-3}) with **poly dAdT – poly dAdT**: a1) **4a** (λ_{exc}=510 nm); a2) Experimental (●) and calculated (–) fluorescence intensities of **4a** at λ_{em} = 535 nm: b1) Fluorimetric titration of **4b** (λ_{exc}=478 nm); b2) Experimental (●) and calculated (–) fluorescence intensities of **4b** at λ = 545 nm; c1) **4c** (λ_{exc}=484 nm); c2) Experimental (●) and calculated (–) fluorescence intensities of **4c** at λ = 538 nm; d1) **4d** (λ_{exc}=484 nm); d2) Experimental (●) and calculated (–) fluorescence intensities of **4d** at λ = 548 nm; e1) **4e** (λ_{exc}=452 nm) e2) Experimental (●) and calculated (–) fluorescence intensities of **4e** at λ = 540 nm

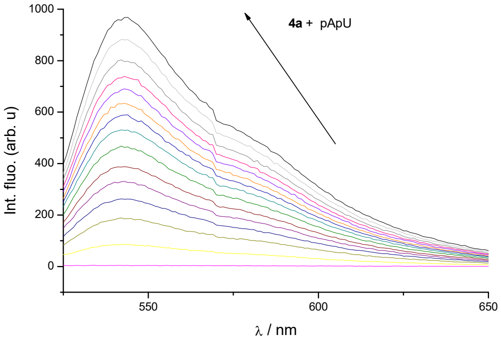

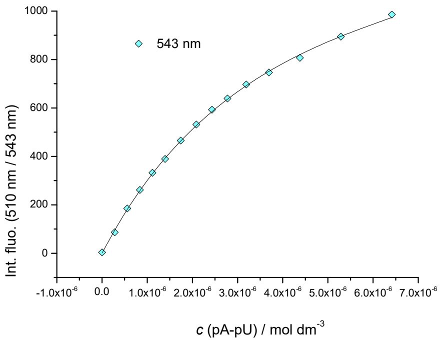

a1)

a2)

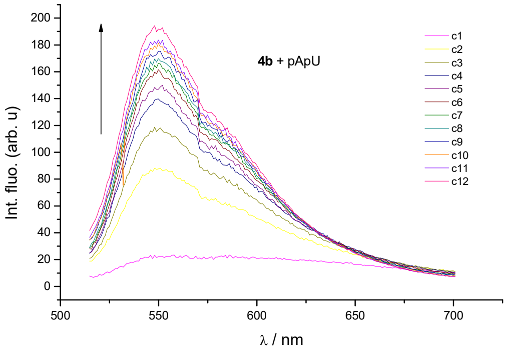

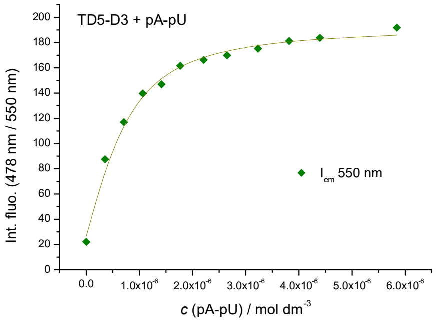

b2)

b1)

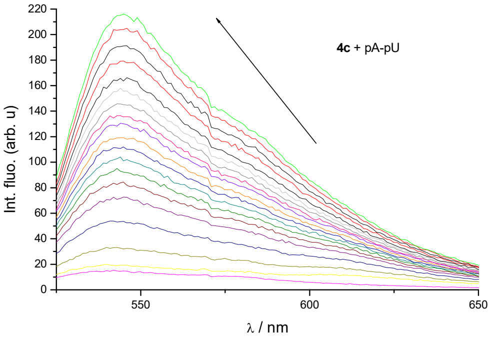

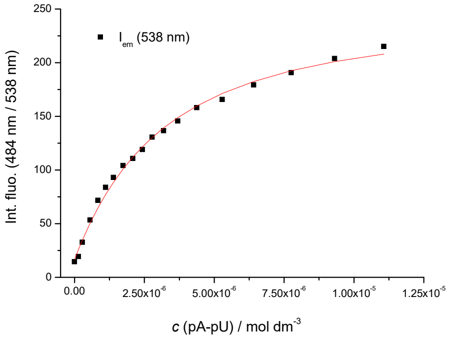

c2)

c1)

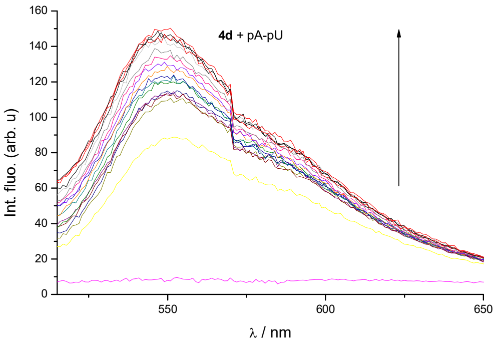

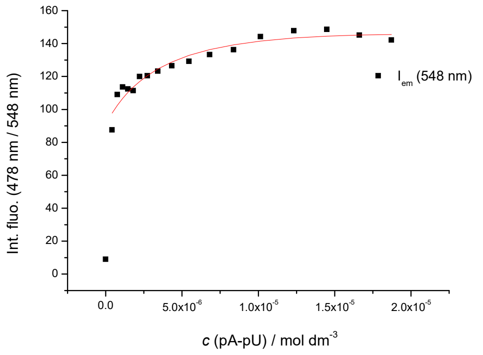

d1)

d2)

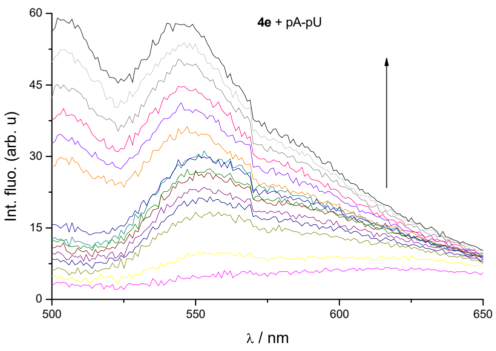

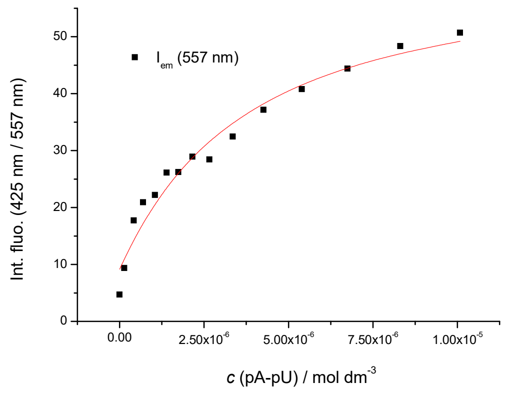

e1)

e1)

Figure S14. Fluorimetric titration of **4a-4e** compounds (c = 1.3-1.5 × 10^{-7} mol dm^{-3}) with **poly rA – poly rU**: a1) **4a** (λ_{exc}=510 nm); a2) Experimental (●) and calculated (–) fluorescence intensities of **4a** at λ_{em} = 543 nm: b1) Fluorimetric titration of **4b** (λ_{exc}=478 nm); b2) Experimental (●) and calculated (–) fluorescence intensities of **4b** at λ = 550 nm; c1) **4c** (λ_{exc}=484 nm); c2) Experimental (●) and calculated (–) fluorescence intensities of **4c** at λ = 538 nm; d1) **4d** (λ_{exc}=484 nm); d2) Experimental (●) and calculated (–) fluorescence intensities of **4d** at λ = 548 nm; e1) **4e** (λ_{exc}=452 nm) e2) Experimental (●) and calculated (–) fluorescence intensities of **4e** at λ = 557 nm

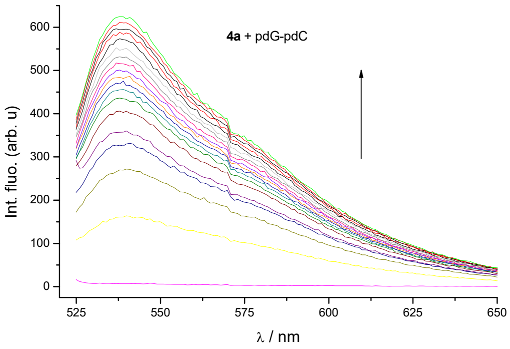

a1)

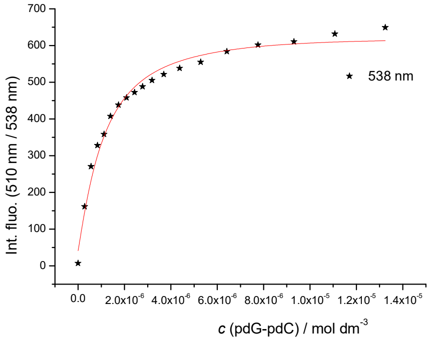

a2)

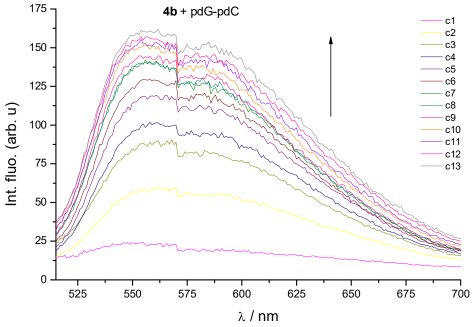

b1)

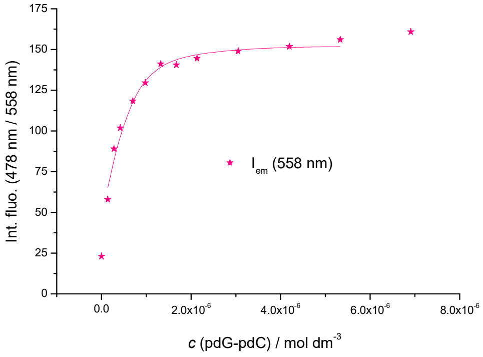

b2)

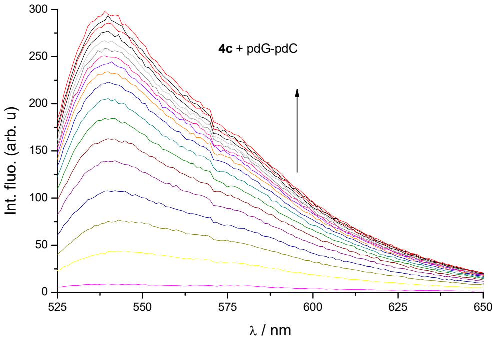

c1)

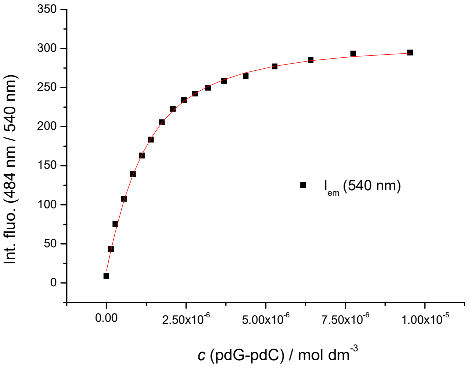

c2)

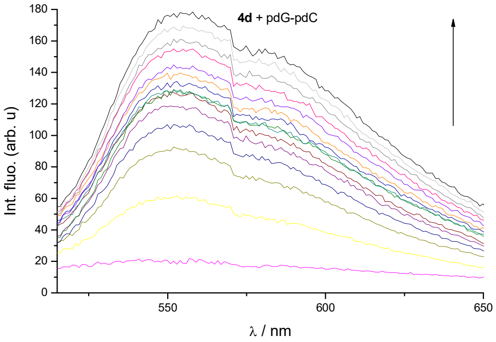

d1)

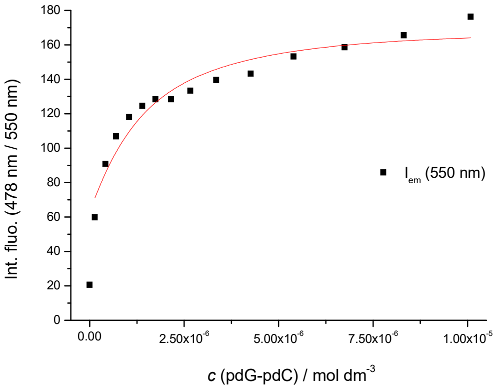

d2)

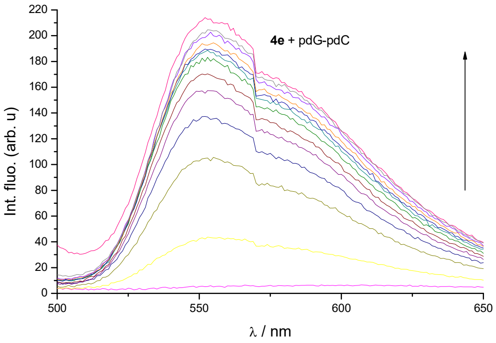

e1)

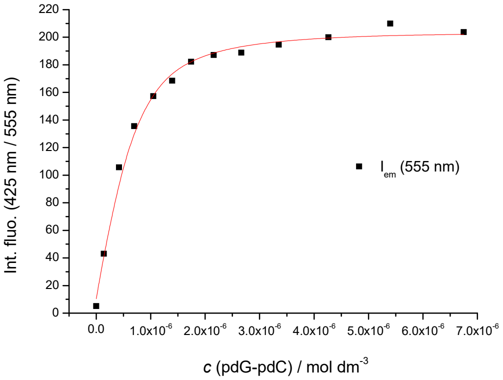

e1)

Figure S15. Fluorimetric titration of **4a-4e** compounds (c = 1.3-1.5 $\times$ 10^{-7} mol dm^{-3}) with **poly dG – poly dC**: a1) **4a** (λ_{exc}=510 nm); a2) Experimental (●) and calculated (–) fluorescence intensities of **4a** at λ_{em} = 538 nm: b1) Fluorimetric titration of **4b** (λ_{exc}=478 nm); b2) Experimental (●) and calculated (–) fluorescence intensities of **4b** at λ = 550 nm; c1) **4c** (λ_{exc}=484 nm); c2) Experimental (●) and calculated (–) fluorescence intensities of **4c** at λ = 538 nm; d1) **4d** (λ_{exc}=484 nm); d2) Experimental (●) and calculated (–) fluorescence intensities of **4d** at λ = 550 nm; e1) **4e** (λ_{exc}=452 nm) e2) Experimental (●) and calculated (–) fluorescence intensities of **4e** at λ = 575 nm

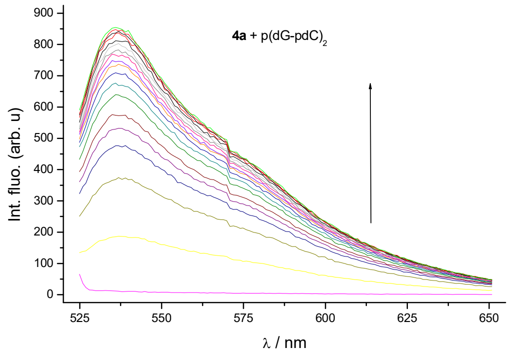

a1)

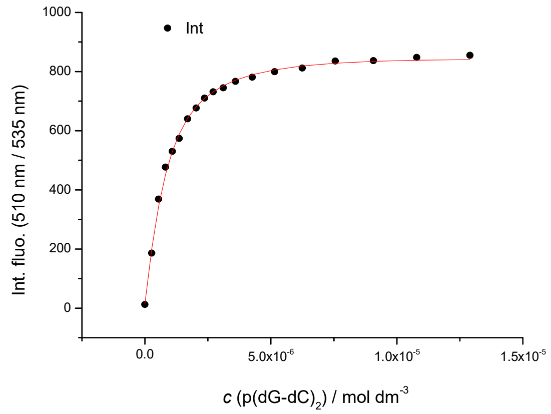

a2)

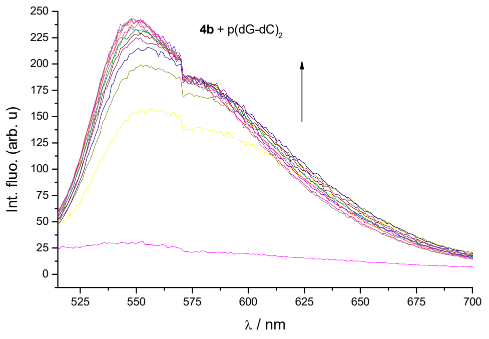

b1)

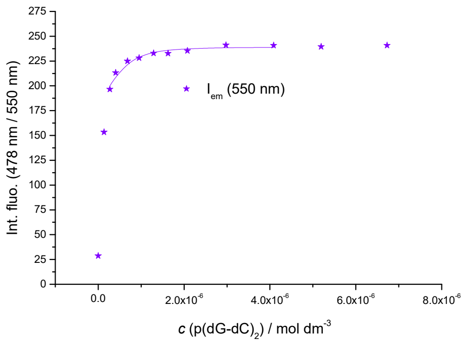

b2)

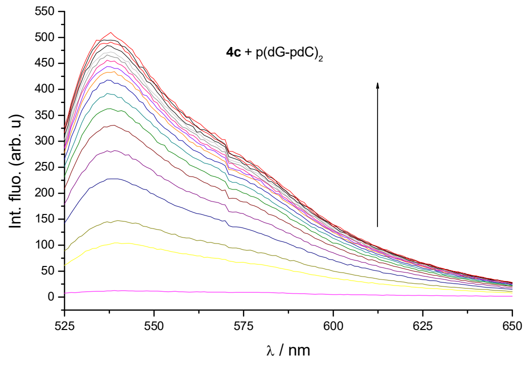

c1)

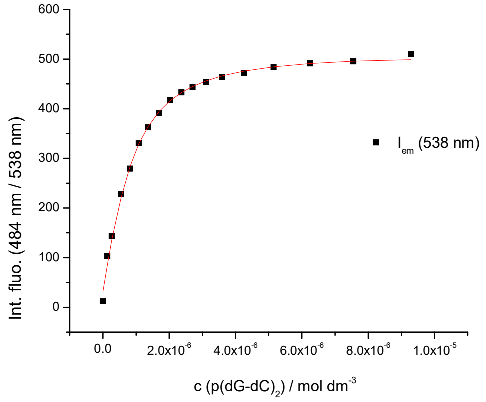

c2)

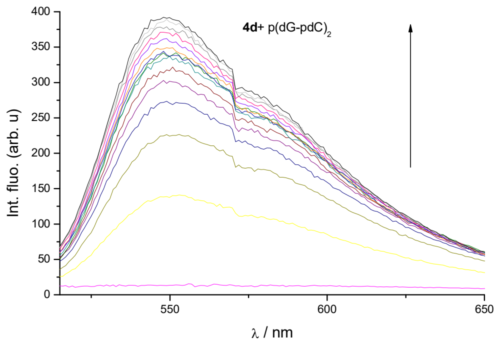

d1)

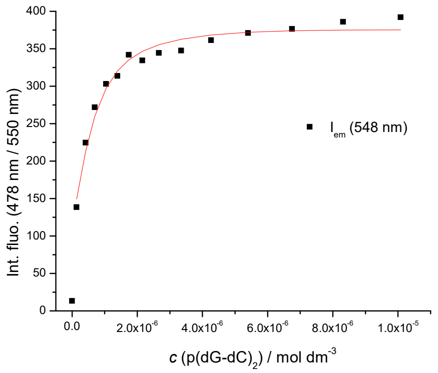

d2)

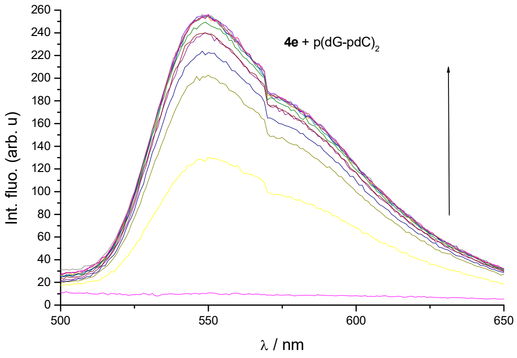

e1)

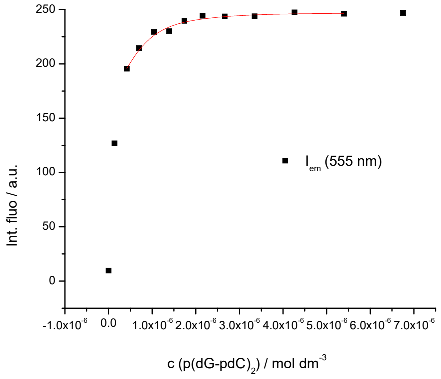

e1)

Figure S16. Fluorimetric titration of **4a-4e** compounds (c = 1.3-1.5 × 10^{-7} mol dm^{-3}) with **polydGdC – poly dGdC**: a1) **4a** (λ_{exc}=510 nm); a2) Experimental (●) and calculated (–) fluorescence intensities of **4a** at λ_{em} = 535 nm: b1) Fluorimetric titration of **4b** (λ_{exc}=478 nm); b2) Experimental (●) and calculated (–) fluorescence intensities of **4b** at λ = 550 nm; c1) **4c** (λ_{exc}=484 nm); c2) Experimental (●) and calculated (–) fluorescence intensities of **4c** at λ = 538 nm; d1) **4d** (λ_{exc}=484 nm); d2) Experimental (●) and calculated (–) fluorescence intensities of **4d** at λ = 548 nm; e1) **4e** (λ_{exc}=452 nm) e2) Experimental (●) and calculated (–) fluorescence intensities of **4e** at λ = 555 nm

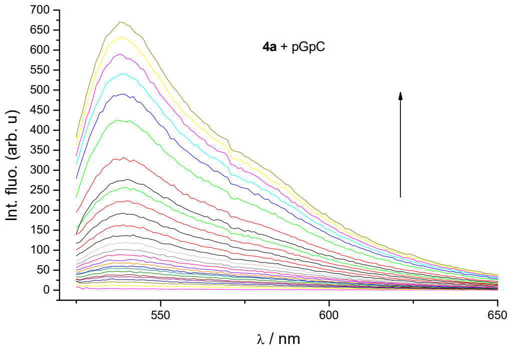

a1)

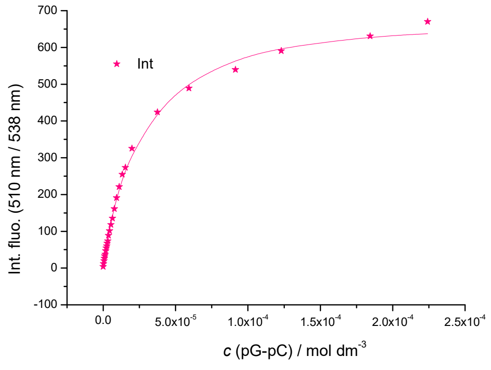

a2)

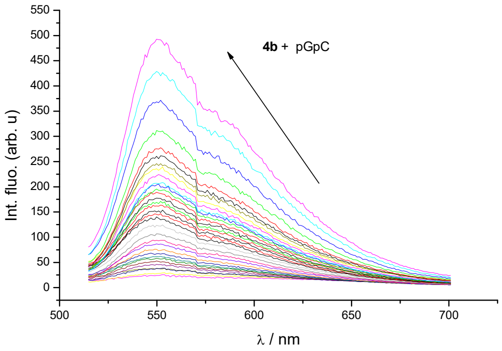

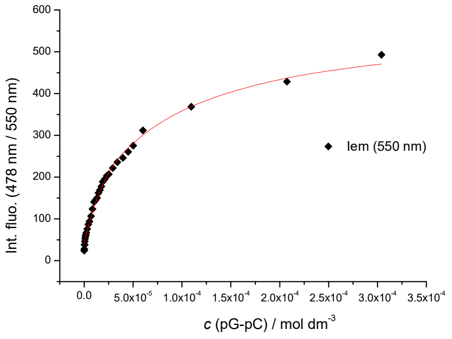

b1)

b2)

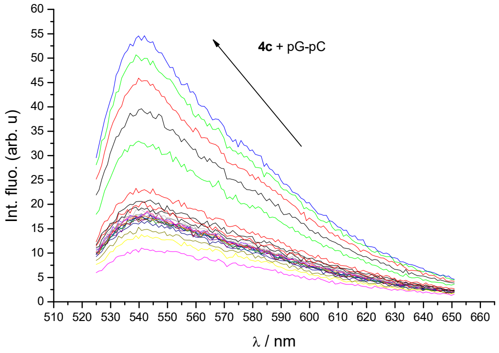

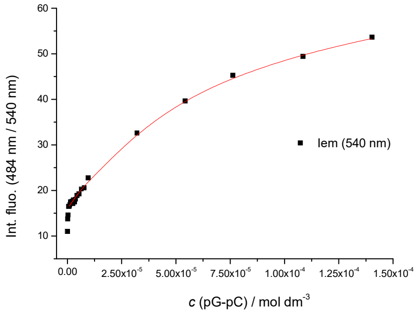

c2)

c1)

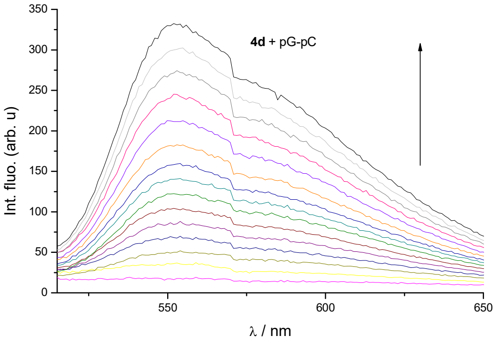

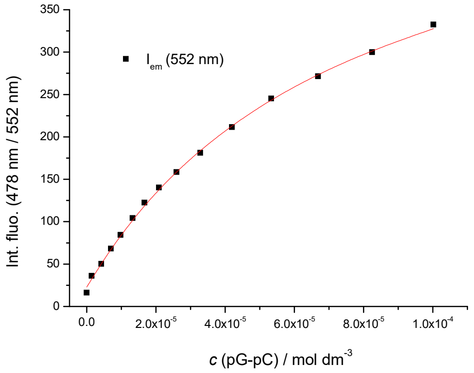

d1)

d2)

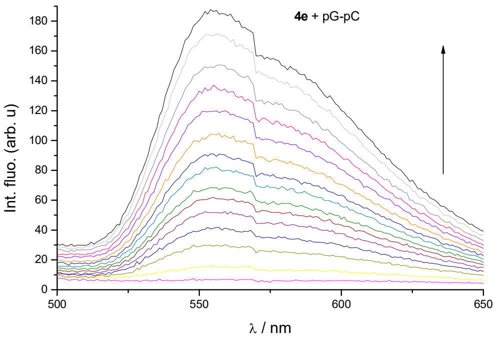

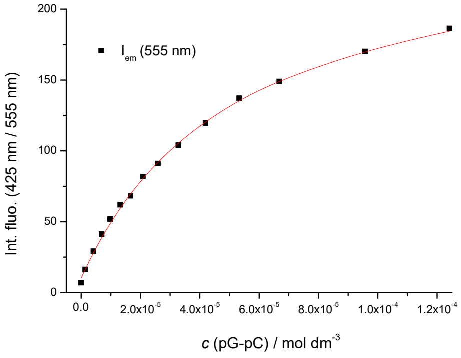

e1) e1)

Figure S17. Fluorimetric titration of **4a-4e** compounds (c = 1.3-1.5 × 10^{-7} mol dm^{-3}) with **poly rG – poly rC**: a1) **4a** (λ_{exc}=510 nm); a2) Experimental (●) and calculated (–) fluorescence intensities of **4a** at λ_{em} = 538 nm: b1) Fluorimetric titration of **4b** (λ_{exc}=478 nm); b2) Experimental (●) and calculated (–) fluorescence intensities of **4b** at λ = 550 nm; c1) **4c** (λ_{exc}=484 nm); c2) Experimental (●) and calculated (–) fluorescence intensities of **4c** at λ = 538 nm; d1) **4d** (λ_{exc}=484 nm); d2) Experimental (●) and calculated (–) fluorescence intensities of **4d** at λ = 548 nm; e1) **4e** (λ_{exc}=452 nm) e2) Experimental (●) and calculated (–) fluorescence intensities of **4e** at λ = 555 nm

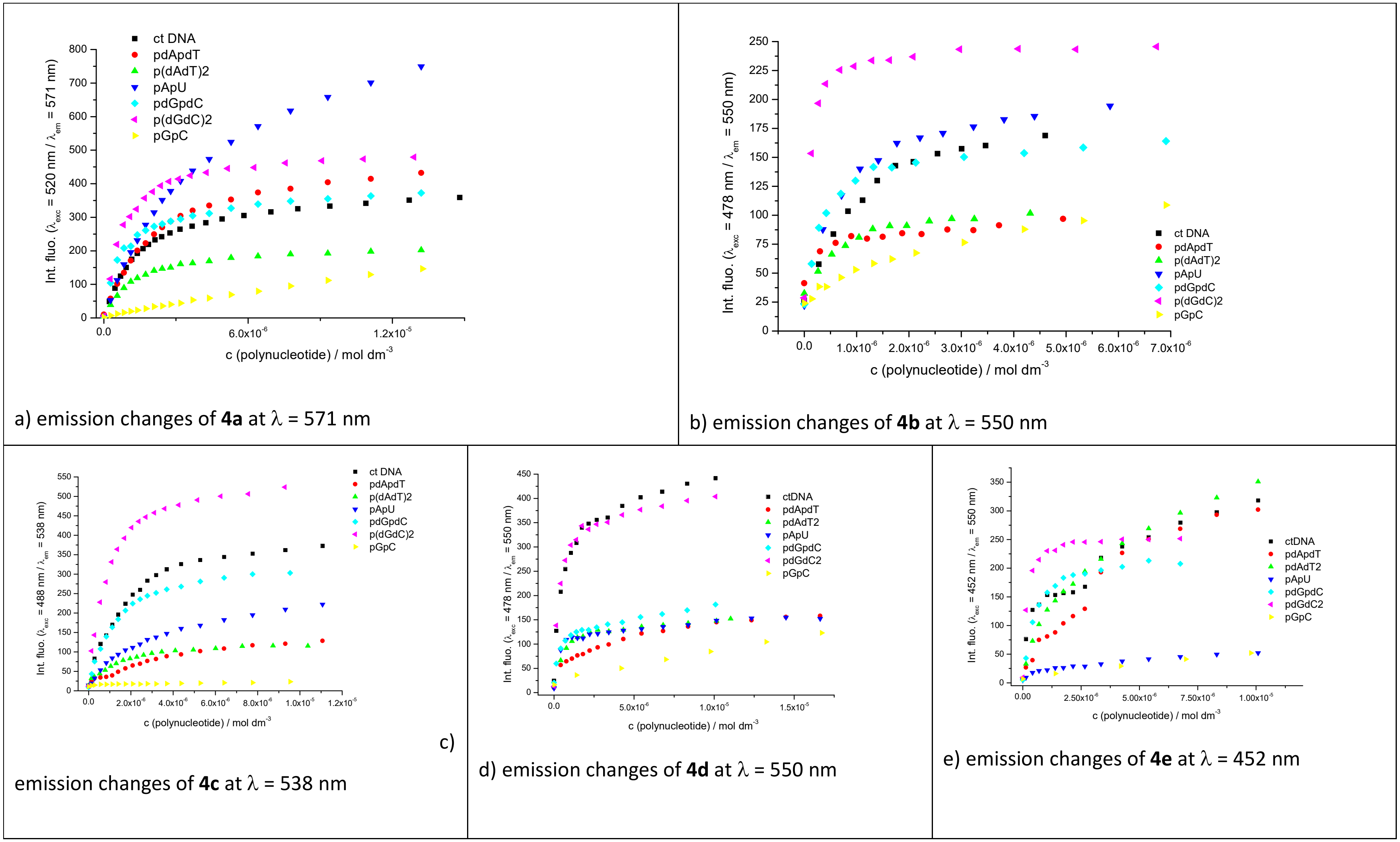

a) emission changes of **4a** at λ = 571 nm

b) emission changes of **4b** at λ = 550 nm

c) emission changes of **4c** at λ = 538 nm

d) emission changes of **4d** at λ = 550 nm

e) emission changes of **4e** at λ = 452 nm

Figure S18. Changes of fluorescence emission at emission maxima of a) **4a** (c = 1.3 × 10^{-7} mol dm^{-3}); b) **4b** u(c = 1.3 × 10^{-7} mol dm^{-3}); c) **4c** (c = 1.48 × 10^{-7} mol dm^{-3}); d) **4d** (c = 1.34 × 10^{-7} mol dm^{-3}); e) **4e** (c = 1.34 × 10^{-7} mol dm^{-3}) upon addition of *ds*-polynucleotides of at pH 7 (Na-cacodylate buffer, pH = 7.0, *I*=0.05 M)

2.3. Circular dichroism (CD) experiments

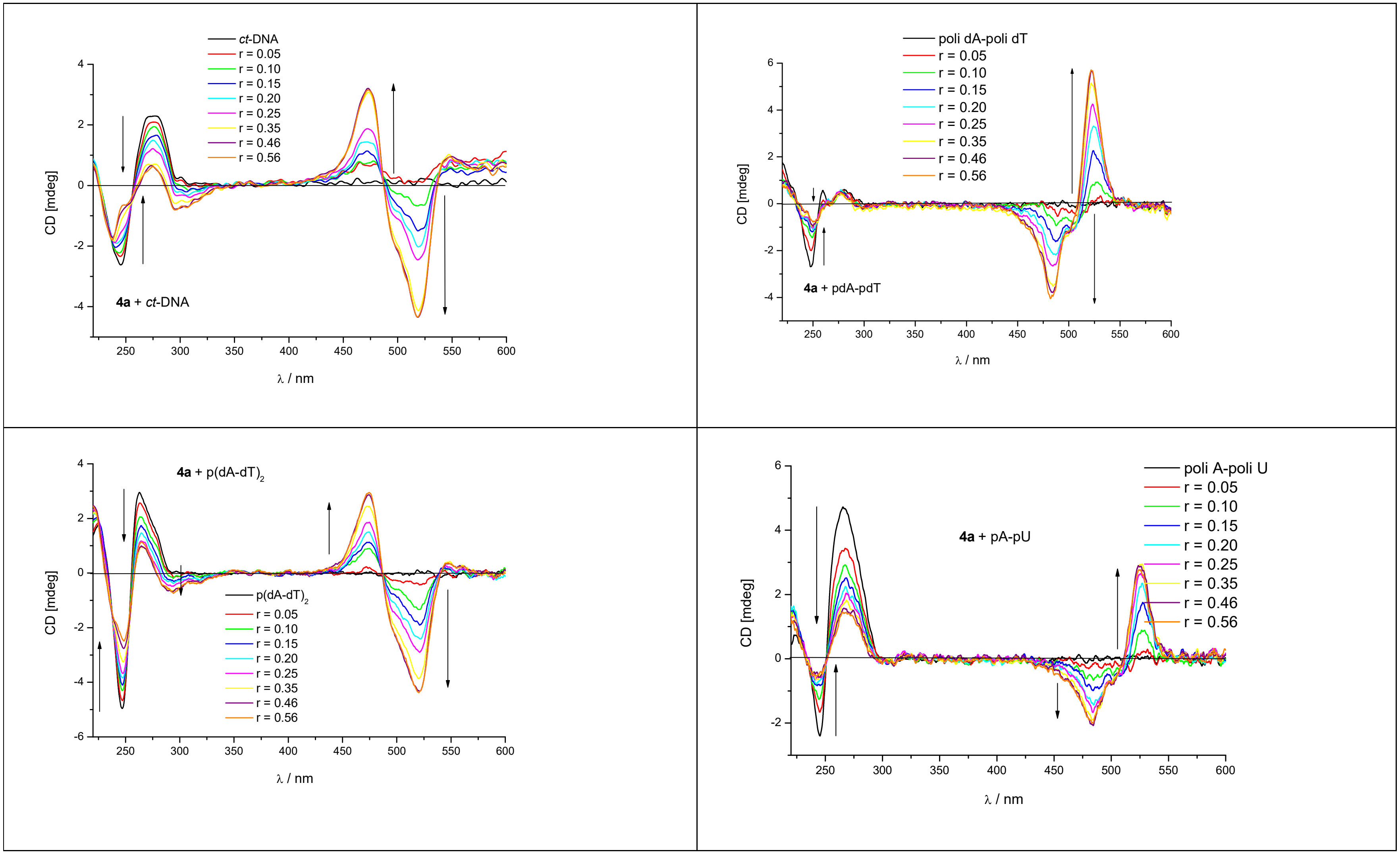

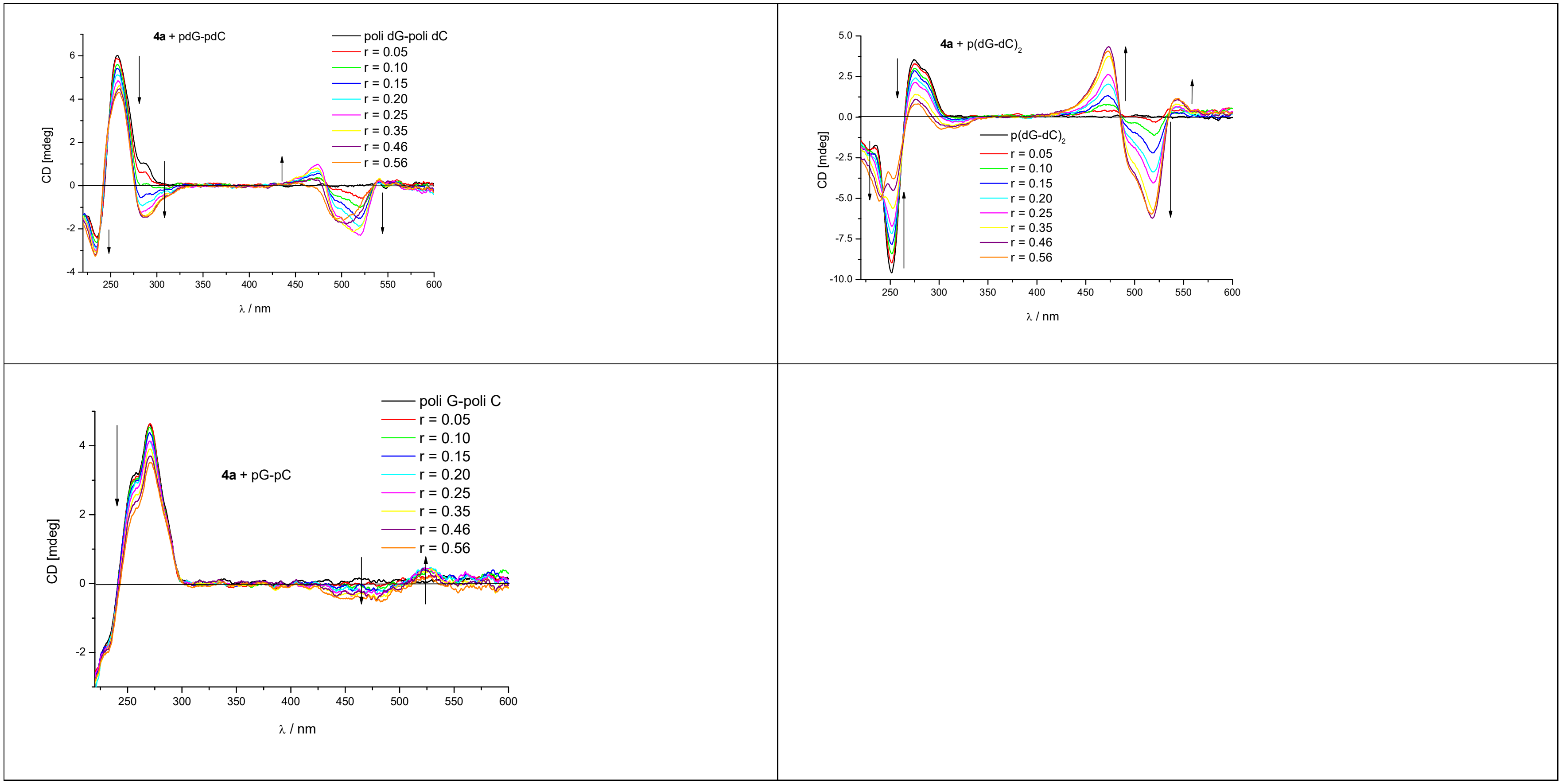

Figure S19. CD titration of polynucleotides (c = 2.0 × 10^{-5} mol dm^{-3}) with **4a** at different molar ratios ***r*** = [compound] / [polynucleotide] (pH = 7.0, buffer sodium cacodylate, I = 0.05 mol dm^{-3}).

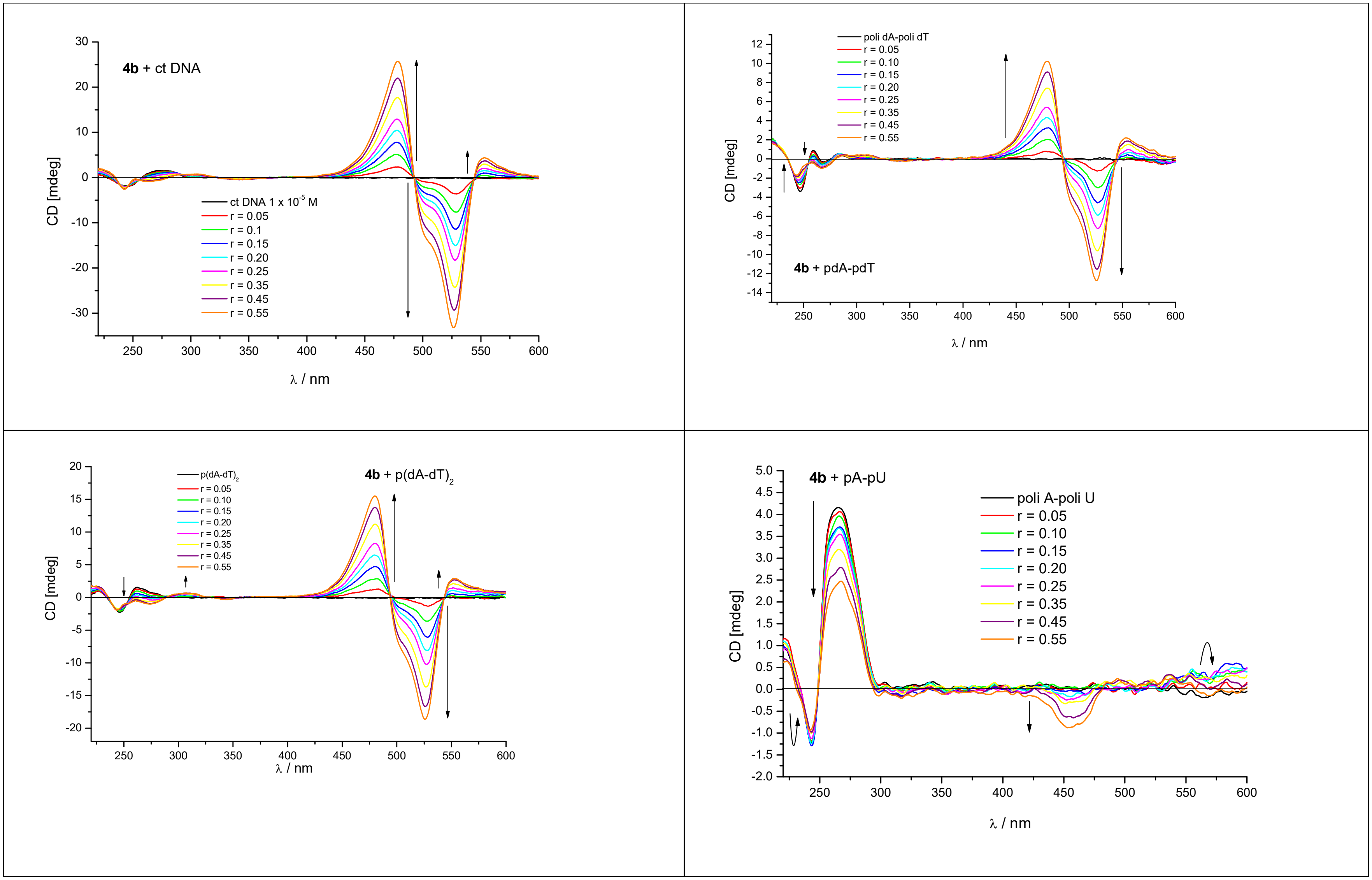

4b + ct DNA
CD [mdeg]
λ / nm
ct DNA 1 x 10-5 M
r = 0.05
r = 0.1
r = 0.15
r = 0.20
r = 0.25
r = 0.35
r = 0.45
r = 0.55
poli dA-poli dT
r = 0.05
r = 0.10
r = 0.15
r = 0.20
r = 0.25
r = 0.35
r = 0.45
r = 0.55
4b + pdA-pdT
CD [mdeg]
λ / nm
p(dA-dT)2
r = 0.05
r = 0.10
r = 0.15
r = 0.20
r = 0.25
r = 0.35
r = 0.45
r = 0.55
4b + p(dA-dT)2
CD [mdeg]
λ / nm
4b + pA-pU
poli A-poli U
r = 0.05
r = 0.10
r = 0.15
r = 0.20
r = 0.25
r = 0.35
r = 0.45
r = 0.55
CD [mdeg]
λ / nm

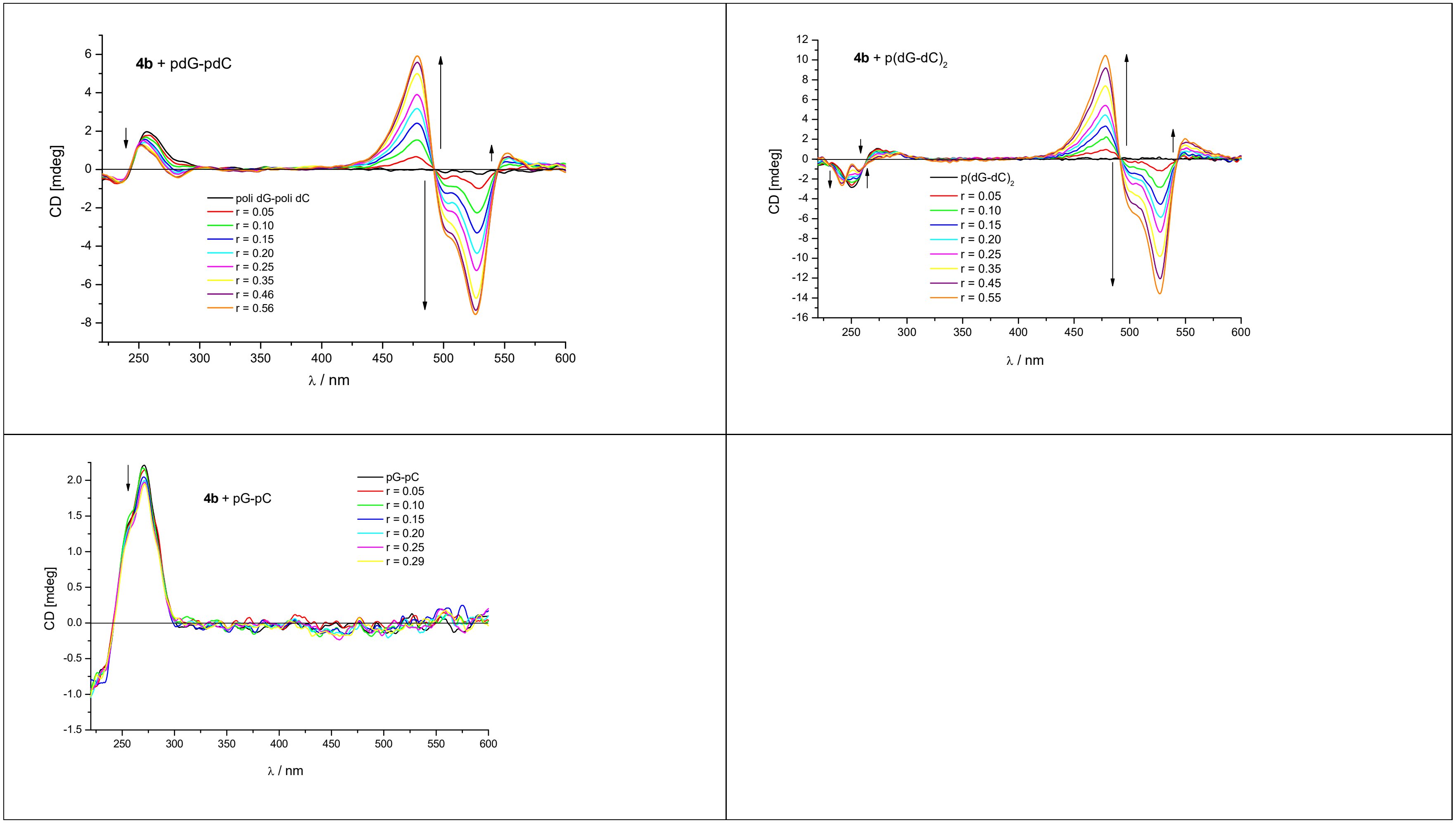

Figure S20. CD titration of polynucleotides (c = 1.0 × 10^{-5} mol dm^{-3}) with **4b** at different molar ratios ***r*** = [compound] / [polynucleotide] (pH = 7.0, buffer sodium cacodylate, I = 0.05 mol dm^{-3}).

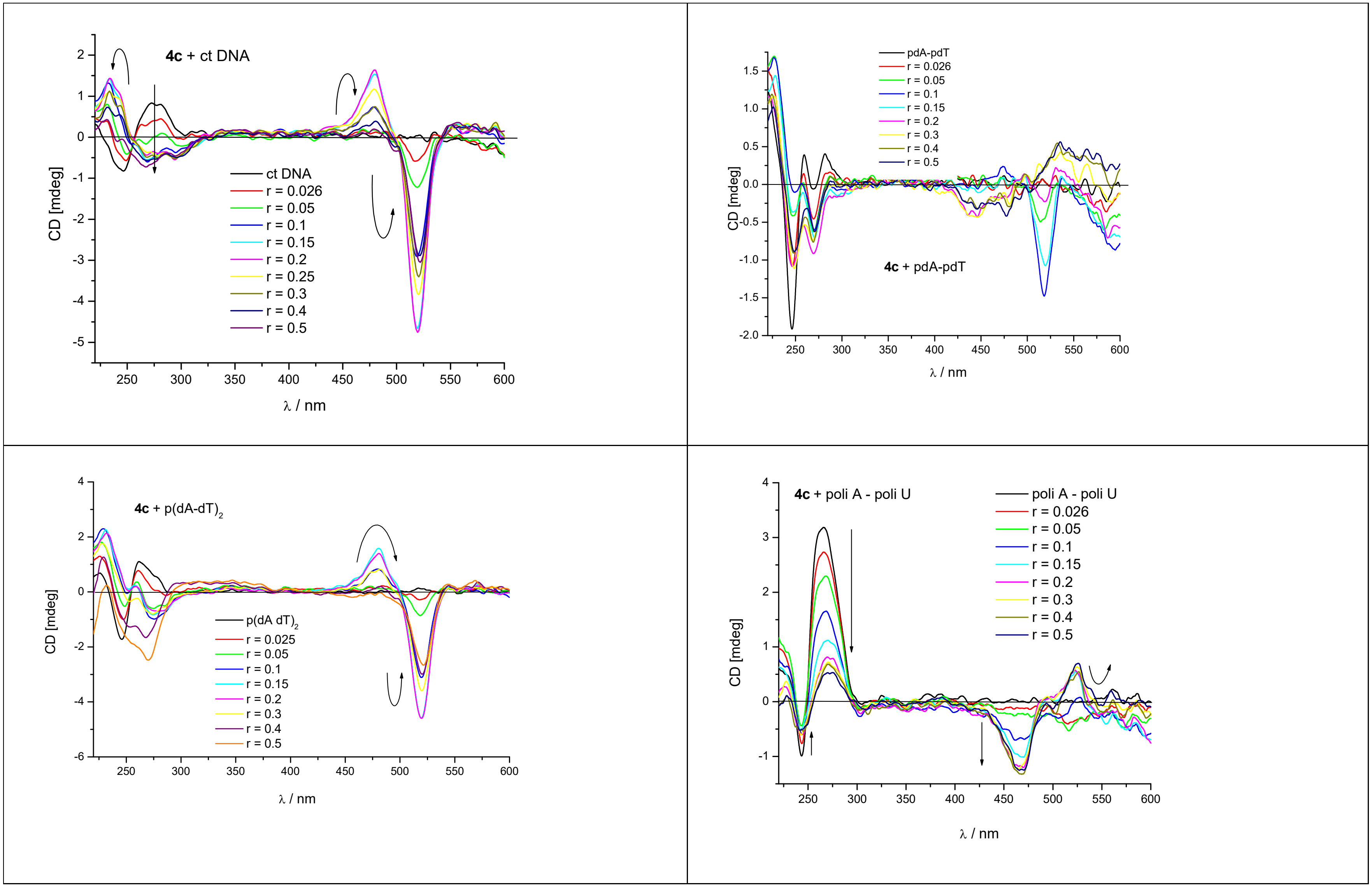
4c + ct DNA
CD [mdeg]
λ / nm
ct DNA
r = 0.026
r = 0.05
r = 0.1
r = 0.15
r = 0.2
r = 0.25
r = 0.3
r = 0.4
r = 0.5
4c + pdA-pdT
pdA-pdT
r = 0.026
r = 0.05
r = 0.1
r = 0.15
r = 0.2
r = 0.3
r = 0.4
r = 0.5
4c + p(dA-dT)2
p(dA dT)2
r = 0.025
r = 0.05
r = 0.1
r = 0.15
r = 0.2
r = 0.3
r = 0.4
r = 0.5
4c + poli A - poli U
poli A - poli U
r = 0.026
r = 0.05
r = 0.1
r = 0.15
r = 0.2
r = 0.3
r = 0.4
r = 0.5

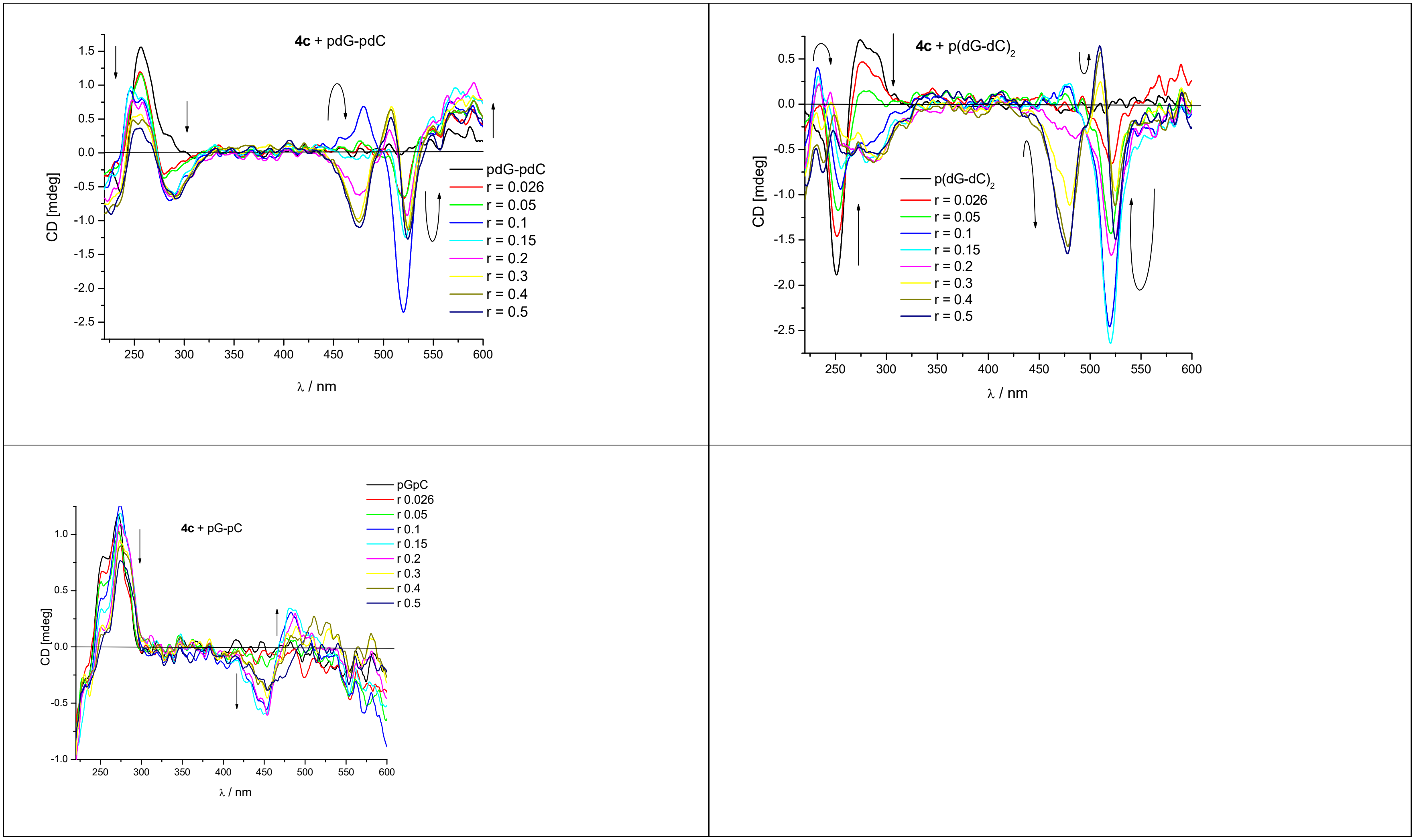

Figure S21. CD titration of polynucleotides ($c = 1.0 \times 10^{-5}$ mol dm^{-3}) with **4c** at different molar ratios ***r*** = [compound] / [polynucleotide] (pH = 7.0, buffer sodium cacodylate, $I = 0.05$ mol dm^{-3}).

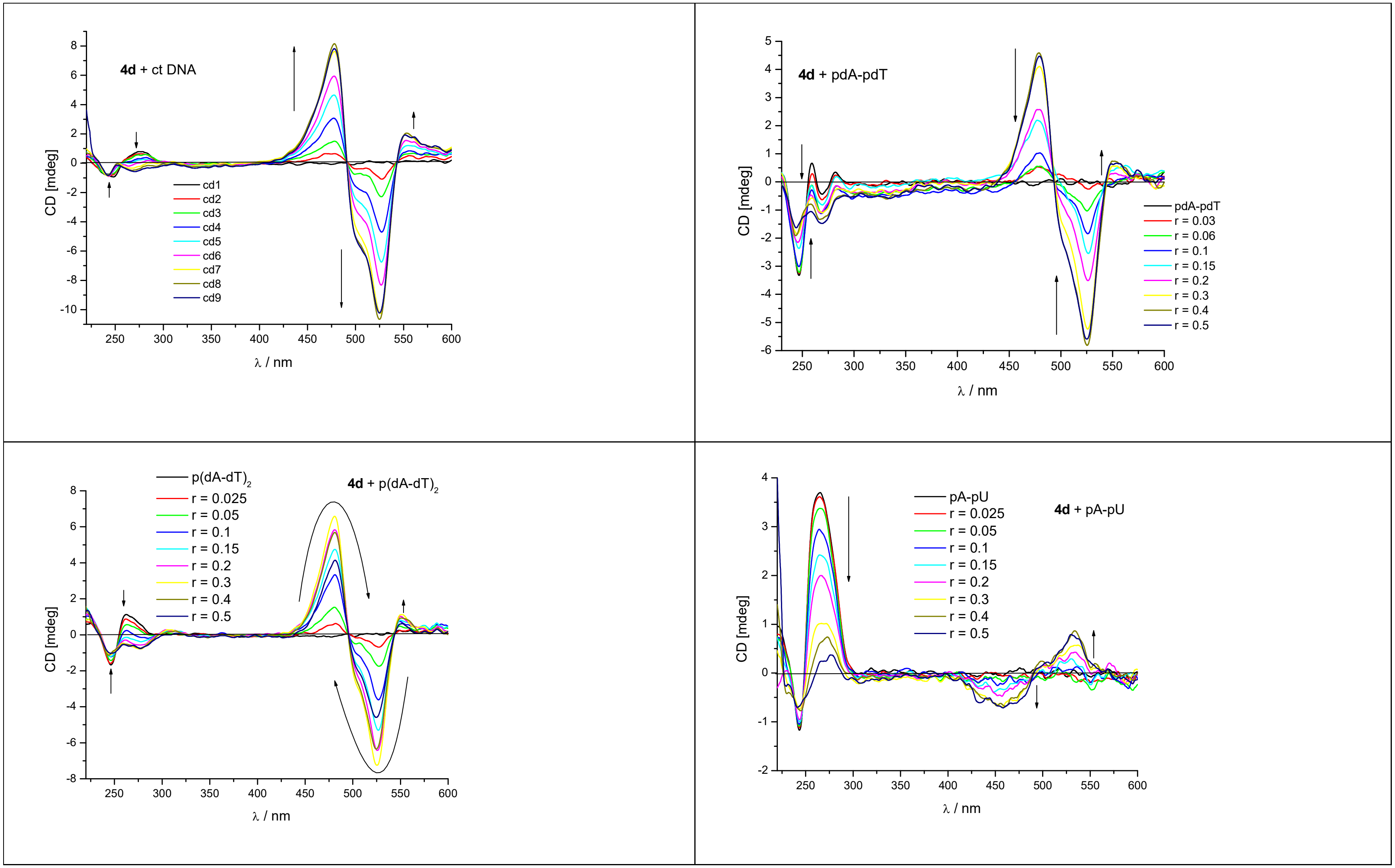
4d + ct DNA
CD [mdeg]
λ / nm
cd1
cd2
cd3
cd4
cd5
cd6
cd7
cd8
cd9
4d + pdA-pdT
pdA-pdT
r = 0.03
r = 0.06
r = 0.1
r = 0.15
r = 0.2
r = 0.3
r = 0.4
r = 0.5
4d + p(dA-dT)2
p(dA-dT)2
r = 0.025
r = 0.05
r = 0.1
r = 0.15
r = 0.2
r = 0.3
r = 0.4
r = 0.5
4d + pA-pU
pA-pU
r = 0.025
r = 0.05
r = 0.1
r = 0.15
r = 0.2
r = 0.3
r = 0.4
r = 0.5

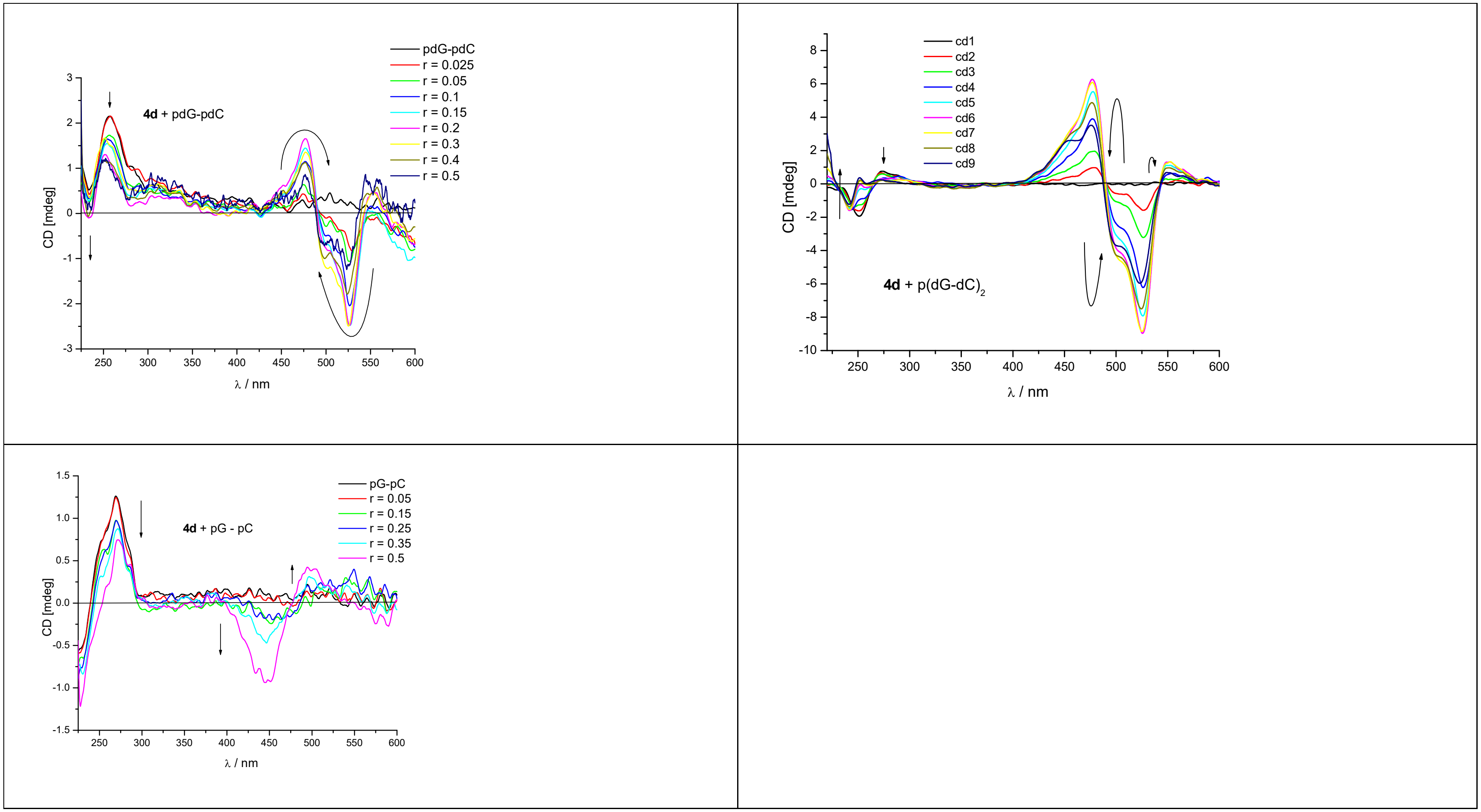

Figure S22. CD titration of polynucleotides ($c = 1.0 \times 10^{-5}$ mol dm^{-3}) with **4d** at different molar ratios ***r*** = [compound] / [polynucleotide] (pH = 7.0, buffer sodium cacodylate, $I = 0.05$ mol dm^{-3}).

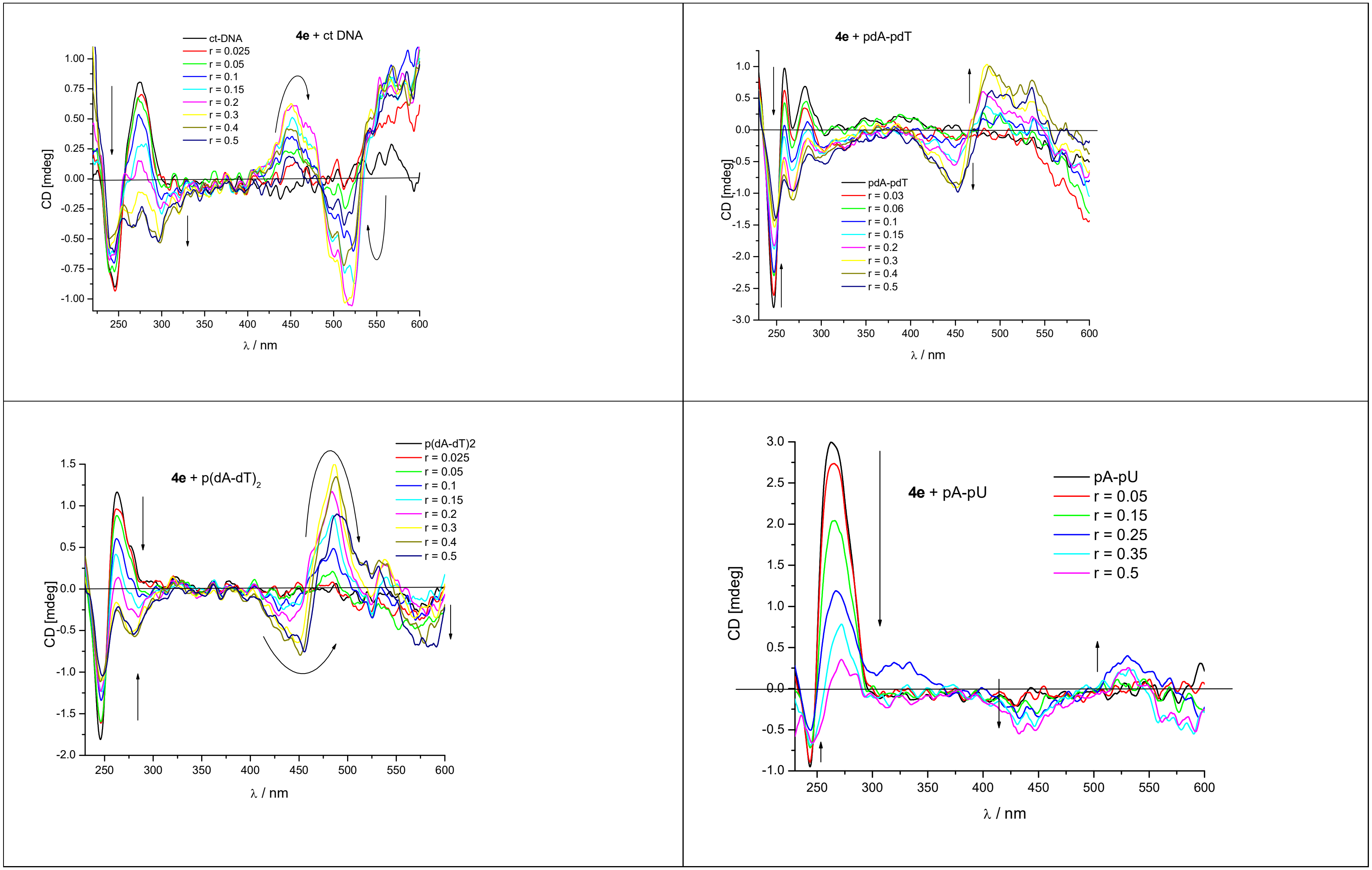

4e + ct DNA
ct-DNA
r = 0.025
r = 0.05
r = 0.1
r = 0.15
r = 0.2
r = 0.3
r = 0.4
r = 0.5
CD [mdeg]
λ / nm
4e + pdA-pdT
pdA-pdT
r = 0.03
r = 0.06
r = 0.1
r = 0.15
r = 0.2
r = 0.3
r = 0.4
r = 0.5
CD [mdeg]
λ / nm
4e + p(dA-dT)2
p(dA-dT)2
r = 0.025
r = 0.05
r = 0.1
r = 0.15
r = 0.2
r = 0.3
r = 0.4
r = 0.5
CD [mdeg]
λ / nm
4e + pA-pU
pA-pU
r = 0.05
r = 0.15
r = 0.25
r = 0.35
r = 0.5
CD [mdeg]
λ / nm

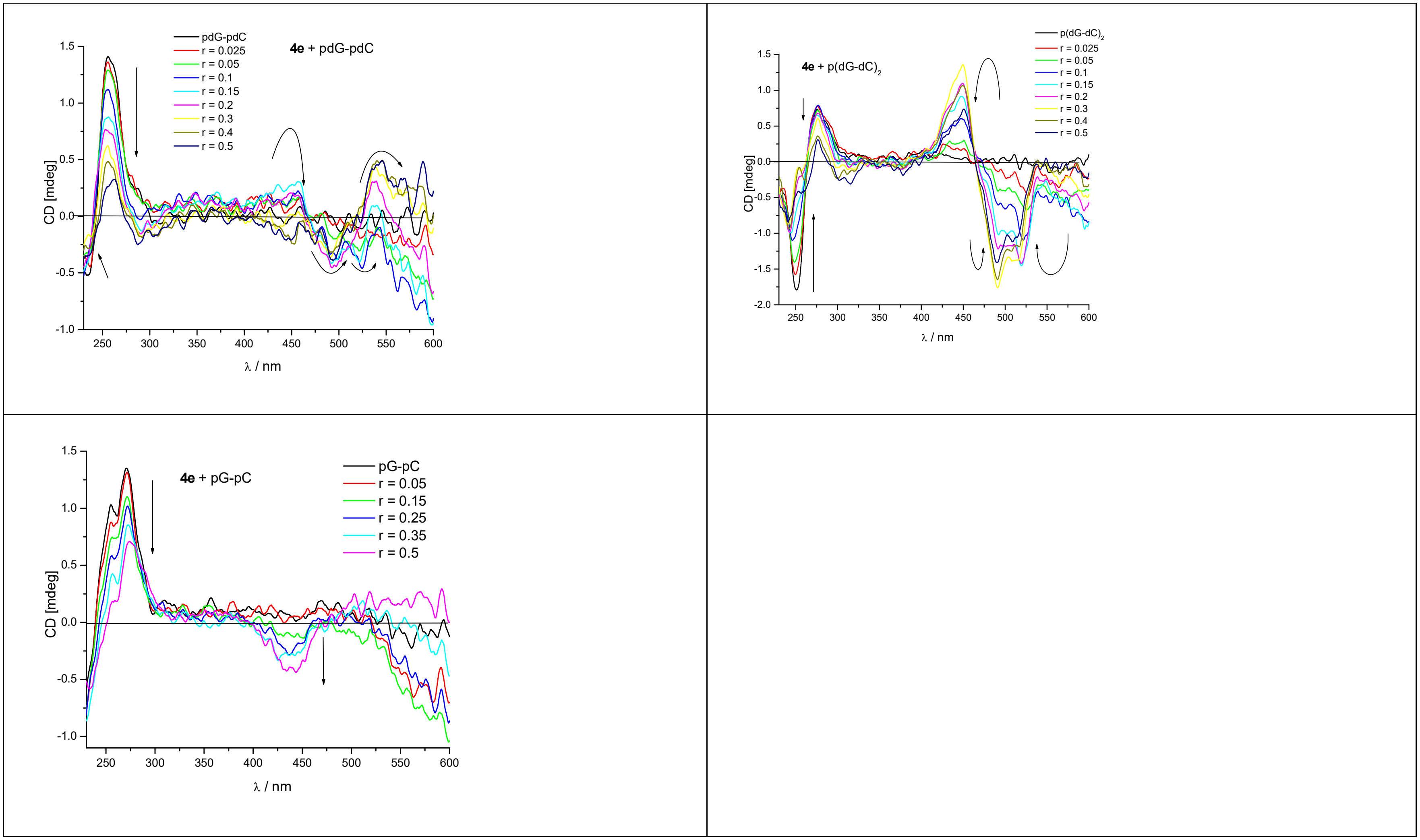

Figure S23. CD titration of polynucleotides (c = 1.0 × 10^{-5} mol dm^{-3}) with **4e** at different molar ratios ***r*** = [compound] / [polynucleotide] (pH = 7.0, buffer sodium cacodylate, I = 0.05 mol dm^{-3}).

3. Biological assays

The purpose of this study was to investigate the effects of compound**s** on the proliferation of different human tumor cell lines.

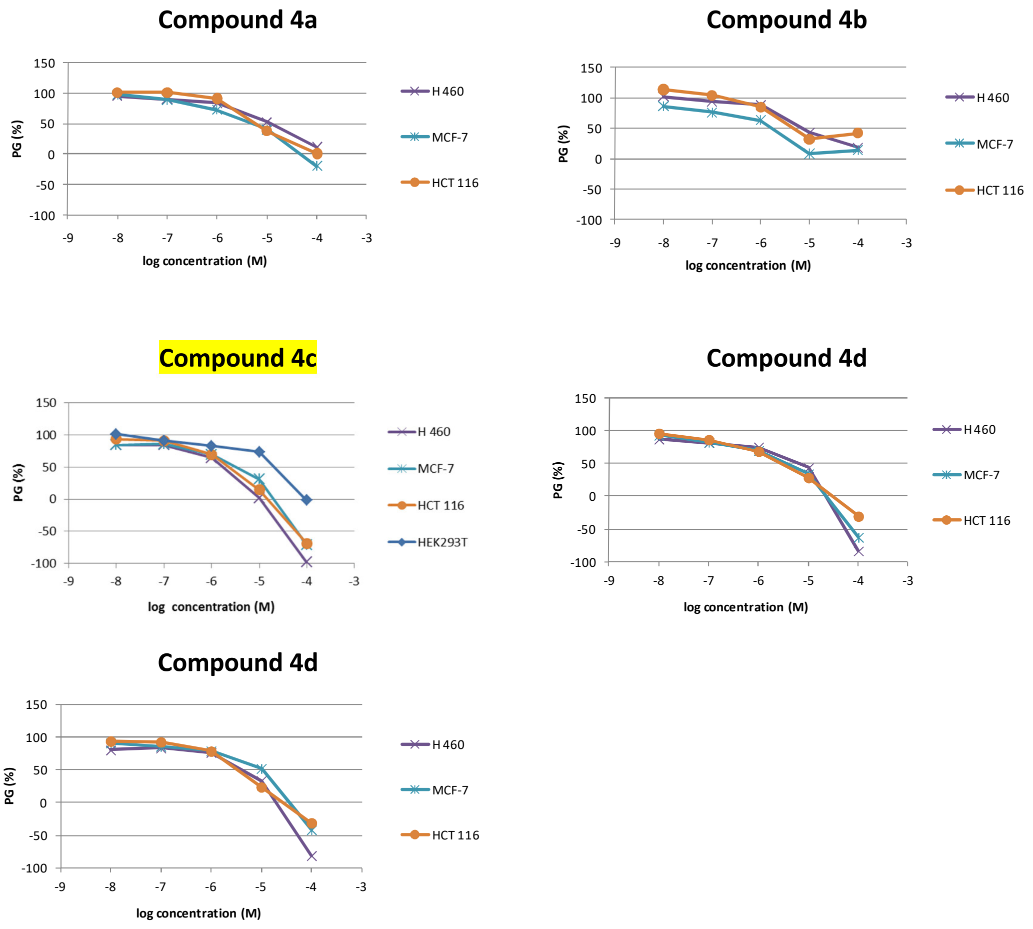

Figure S24. Dose-response profiles for compound **4a-4e** compounds tested *in vitro*.

GI_{50} and LC_{50} calculations

The GI_{50} measures the growth inhibitory power of the test agent and represents the concentration that causes 50% growth inhibition, while the LC_{50} signifies a cytotoxic effect and represents the concentration that kills 50% of initial cell number. The GI_{50} and LC_{50} values for each compound are calculated from dose-response curves using linear regression analysis by fitting the test concentrations that give PG values above and below the respective reference value (e.g. 50 for GI_{50}). Therefore, a "real" value for any of the response parameters is obtained only if at least one of the tested drug concentrations falls above, and likewise at least one falls below the respective reference value. If however, for a given cell line all of the tested concentrations produce PGs exceeding the respective

reference level of effect (e.g. PG value of 50), then the highest tested concentration is assigned as the default value. In the screening data report, that default value is preceded by a ">" sign.

---

[1] W. Saenger, *Principles of Nucleic Acid Structure,* Springer-Verlag: New York, 1983; p. 226.

[2] G. Scatchard, *Ann. N.Y. Acad. Sci.*, 1949, **51**, 660-672.; J.D. McGhee, P.H. von Hippel, *J. Mol. Biol.,* 1976, **103,** 679-684.
